# Molecular Signatures of Human Chronic Atrial Fibrillation in Primary Mitral Regurgitation

**DOI:** 10.1155/2021/5516185

**Published:** 2021-10-15

**Authors:** Günseli Çubukçuoğlu Deniz, Serkan Durdu, Yeşim Doğan, Esra Erdemli, Hilal Özdağ, Ahmet Ruchan Akar

**Affiliations:** ^1^Stem Cell Institute, Ankara University, Ankara, Turkey; ^2^Biotechnology Institute, Ankara University, Ankara, Turkey; ^3^Department of Cardiovascular Surgery, Heart Center, Ankara University School of Medicine, Ankara, Turkey; ^4^Department of Histology and Embryology, Ankara University School of Medicine, Ankara, Turkey

## Abstract

**Objectives:**

Transcriptomics of atrial fibrillation (AFib) in the setting of chronic primary mitral regurgitation (MR) remains to be characterized. We aimed to compare the gene expression profiles of patients with degenerative MR in AFib and sinus rhythm (SR) for a clearer picture of AFib pathophysiology.

**Methods:**

After transcriptomic analysis and bioinformatics (*n* = 59), differentially expressed genes were defined using 1.5-fold change as the threshold. Additionally, independent datasets from GEO were included as meta-analyses.

**Results:**

QRT-PCR analysis confirmed that AFib persistence was associated with increased expression molecular changes underlying a transition to heart failure (*NPPB*, *P* = 0.002; *ANGPTL2*, *P* = 0.002; *IGFBP2*, *P* = 0.010), structural remodeling including changes in the extracellular matrix and cellular stress response (*COLQ*, *P* = 0.003; *COMP*, *P* = 0.028; *DHRS9*, *P* = 0.038; *CHGB*, *P* = 0.038), and cellular stress response (*DNAJA4*, *P* = 0.038). Furthermore, AFib persistence was associated with decreased expression of the targets of structural remodeling (*BMP7*, *P* = 0.021) and electrical remodeling (*CACNB2*, *P* = 0.035; *MCOLN3*, *P* = 0.035) in both left and right atrial samples. The transmission electron microscopic analysis confirmed ultrastructural atrial remodeling and autophagy in human AFib atrial samples.

**Conclusions:**

Atrial cardiomyocyte remodeling in persistent AFib is closely linked to alterations in gene expression profiles compared to SR in patients with primary MR. Study findings may lead to novel therapeutic targets. This trial is registered with ClinicalTrials.gov identifier: NCT00970034.

## 1. Introduction

Atrial fibrillation (AFib) is the most widespread acquired cardiac rhythm disorder seen in clinical practice and is recognized as a global epidemic and a significant healthcare burden [1, 2]. The prevalence of AFib is 1% to 4% in the adult population and currently affects more than 33 million individuals worldwide [1, 3]. Advanced age, hypertension, obesity, diabetes, ischemic and valvular heart diseases, heart failure, smoking, heavy alcohol consumption, hyperthyroidism, and genetic polymorphisms are related to an increased risk of progressing AFib. A recent meta-analysis of genome-wide association studies reported 57 AFib-associated genes involving cardiac developmental, electrophysiological, contractile, and structural pathways [4]. The onset of paroxysmal, persistent, or permanent AFib is associated with increased mortality and morbidity from embolic stroke, heart failure, myocardial infarction, and dementia [1, 2]. Patients with chronic primary mitral regurgitation (MR) also have an increased risk of developing AFib, representing complex transcriptomic and molecular mechanisms.

Calcium handling abnormalities, electrical, structural, and autonomic nerve remodeling may all contribute to atrial arrhythmogenic remodeling in the persistence of AFib [3, 5, 6]. Improved understanding of the molecular mechanisms involved in AFib pathogenesis is essential for developing novel biomarkers, genetic and pharmacological therapeutic approaches, and optimal ablation strategies [3, 7]. Over the last decade, the genetic architecture of AFib has been intensely investigated. Studies that are aimed at clarifying the molecular pathophysiology of AFib demonstrated changes in gene expressions, ion channel expression profiling, inflammation, oxidation, and cellular stress responses during AFib [8, 9]. Inflammation and immune response have significant roles in the initiation and maintenance of AFib. Through all complex molecular mechanisms of AFib, extracellular matrix alterations and atrial fibrosis also have essential roles in the pathogenesis of AFib [10].

Additionally, left and right atrial tissue gene expression profiles are not the same in sinus rhythm (SR) and during AFib [11]. Until recently, no study has focused on gene expression profile difference between left and right atrial tissues in AFib and primary MR [12]. In fact, currently available studies investigating the role of transcriptomics in AFib are diverse. However, the main issue that overshadows most of these studies is the noise that arises in high-throughput analyses, which is a consequence of utilizing heterogeneous groups in investigating complex diseases such as AFib.

We identified severe chronic primary MR as a homogenous target population for atrial arrhythmogenic remodeling. We aimed to investigate the human left and right atrial gene expression profiles in patients with severe chronic primary MR and compare persistent AFib with SR.

## 2. Materials and Methods

This study is registered to ClinicalTrials.gov database with the accession number of “ClinicalTrials.gov Identifier: NCT00970034”.

### 2.1. Patients and Atrial Samples

**Figure 1:**
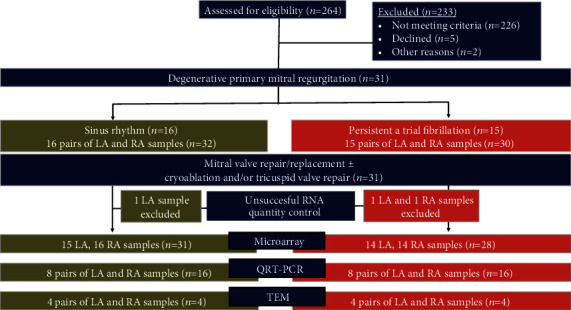
Flow diagram of experimental design.

Patients who were scheduled to undergo mitral valve repair/replacement for chronic primary severe MR were eligible to participate in the study (Figure 1). The exclusion criteria were rheumatic heart disease, secondary MR, infective endocarditis, connective tissue disease, cleft mitral valve, radiation-induced heart disease, hyperthyroidism, obstructive sleep apnea, history of alcohol abuse, prior sternotomy, and major concomitant procedures other than tricuspid valve repair and AFib ablation. Patient demographics and clinical characteristics are presented in Table 1. All participating patients signed informed consent before surgery. Human left atrial (LA) and right atrial (RA) tissue samples were obtained from patients with persistent AFib (*n* = 15) and SR (*n* = 16) undergoing mitral valve surgery. Persistent AFib was defined as AFib that lasts more than seven days, including episodes terminated by cardioversion, either with drugs or by direct current cardioversion, after ≥ seven days. The Local Ethics Committee approved the study protocol of Ankara University (14 Aug 2008/136-3994), and the study conformed to the 1975 Declaration of Helsinki principles. We compared AFib vs. SR in only RA, only LA, and RA + LA tissues, so we had 3 main comparisons in our study.

Transcriptional profiling studies have been done in different organisms and tissues of AFib patients or models (Supp. Table-1). Independent datasets from GEO were included in the analysis to confirm our findings. The GEO database includes 11 human AFib microarray studies (Supp. Table-1). Among them, only two studies conducted on the Affymetrix platform, (GSE2240 [13] and GSE41177 [14]) containing human atrial tissue AFib and SR (*n*_AFib_ = 10; *n*_SR_ = 20 from GSE2240 study and *n*_AFib_ = 32; *n*_SR_ = 6 from GSE41177 study); samples were chosen for final meta-analysis (Supp. Table-2).

### 2.2. Atrial Sample Preparation

Right and left atrial appendage biopsies were obtained during RA cannulation before cardiopulmonary bypass (CPB) and during LA appendage linear closure on CPB. We performed a novel microdissection technique for thin sections of atrial tissue samples under sterile conditions (Supp. Figure-1). Then, samples were immediately snap-frozen in liquid nitrogen intraoperatively and stored at −80°C until assayed.

### 2.3. Gene Expression Profiling

Total RNA was extracted from LA and RA with MELT™ Total Nucleic Acid Isolation System (P/N AM1983 Ambion, Life Technologies, Thermo Fisher Scientific). The quality and quantity of total RNA were assessed by Agilent 2100 Bioanalyzer RNA 6000 PicoChip (Agilent Technologies Inc., USA), NanoDrop® ND-1000 (NanoDrop Technologies, Wilmington, DE, USA) spectrophotometer and agarose gel electrophoresis. The absorbance ratio (260/280 nm) between 1.8 and 2.2 was accepted on a spectrophotometer, and RIN (R.N.A. integrity number) of all total R.N.A. samples extracted from fibrotic human atrial tissues were 5.95 ± 1.4.

500 ng of total RNA extracted from each human atrium sample was utilized to generate amplified and biotinylated cRNA with GeneChip® 3′ IVT Express Kit (P/N 901229 Affymetrix Inc., Santa Clara, CA, USA). 15 *μ*g of fragmented biotin-labeled cRNA was hybridized to Human Genome U133 Plus 2.0 Affymetrix GeneChips (P/N 900467 Affymetrix Inc., Santa Clara, CA, USA). Arrays were washed, stained using the GeneChip® Fluidics Station 450, and finally scanned using the GeneChip® Scanner 3000 7G in conformity with the Affymetrix procedure.

### 2.4. Microarray Data Analysis

**Figure 2:**
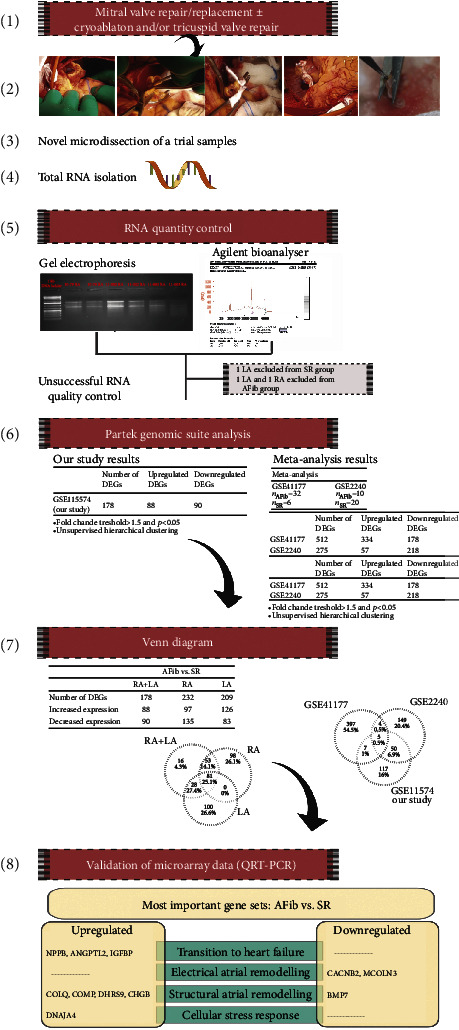
Overview of study steps and schematic representation of the study findings.

The raw array data in CEL format files were uploaded to Partek Genomic Suite (PGS, V6.6, St. Louis, MO). Preprocessing of the probe level data was compiled and transformed using the Robust Multiarray Analysis algorithm. Fold change threshold of >1.5 and *P* < 0.05 limits were applied to filter the differentially expressed transcript list. Adjusted *P* values called *q* values were calculated according to optimized false discovery rate (FDR) [15]. FDR threshold was *q* < 0.05, considered as significant. Unsupervised hierarchical clustering was used to evaluate the relationships between AFib and SR groups.  Figure 2 summarizes all microarray data analyses, including meta-analysis. We also submitted the dataset of gene expression microarrays at GEO (Gene Expression Omnibus) database with accession number GSE115574.

We performed functional enrichment analysis using DEGs. Differentially regulated transcripts (Affy IDs) between persistent AFib and sinus rhythm groups were uploaded to the WebGestalt (WEB-based Gene SeT AnaLysis Toolkit, http://www.webgestalt.org/) software for annotation, enrichment, and visualization of these coding genes. Gene Ontology (GO) and pathway analysis were performed on the WebGestalt toolkit [16]. Functional enrichment analysis runs through overrepresentation analysis (ORA) method and multiple test adjustment with FDR threshold 0.05.

### 2.5. Meta-Analysis

We intended to analyze GSE41177 and GSE2240 datasets together with our data. However, data obtained from different studies were stemmed from different tissue types. Gene expression data from GSE41177 study were from left atrium-pulmonary vein junction (LA-PV) and left atrial appendages (LAA) of patient's undergoing open valvular heart disease surgery, and right atrial tissues were used in GSE2240 study. In GSE2240 study, they collected 30 patient's RAAs undergoing mitral valve repair or coronary artery bypass grafting either with permanent AFib or SR.

First, we analyzed GSE41177 and GSE2240 cel files separately and using all samples without exclusion of any tissue type in each dataset, according to fold change ± 1.5 and adjusted *P* value = *q* value < 0.05 criteria based on the comparison between AFib versus SR (Supp. Table-2).

The aim of the GSE41177 study was to investigate regional gene expression differences between LA-PV and LAA of patient's either with AFib or with SR. Thus, they did not compare AFib to SR with their raw data. We compared AFib vs. SR using GSE41177 raw data and got differentially expressed genes (DEGs) of AFib vs. SR (Supp. Table-3). We also compared AFib vs. SR using GSE2240 raw data in similar manner (Supp. Table-4). We used Venn diagrams to check common elements in DEG sets of our study, GSE2240, and GSE41177 (Supp. Table-5).

Additionally, we performed functional enrichment and pathway analysis through ORA and FDR cutoff 0.05 on GSE41177 and GSE2240 datasets on WebGestalt web tool.

### 2.6. Validation of Microarray Data

We chose genes for validation of microarray from DEG lists (Supp. Tables: 3, 4, 6, 7, 8). First, the intersection of DEGs obtained from AFib vs. SR comparisons of tissues RA + LA, only RA, and only LA evaluated. Secondly, we overlapped those intersection genes with GSE2240 and GSE41177 DEGs using Venn diagrams. We chose common DEGs to validate our microarray data (Supp. Table-10).

Real-time qRT-PCR (qPCR) was performed to validate microarray gene expression data. 1 *μ*g of total RNA was reverse-transcribed using random hexamers provided with Roche Transcriptor First Strand cDNA Synthesis Kit (F. Hoffmann-La Roche Ltd., Switzerland) conforming with the manufacturer's protocol. 1 *μ*g of total RNA was reverse-transcribed in a total of 20 *μ*l volume using random hexamer. cDNA samples were diluted five times, and 2 *μ*l of the diluted cDNA sample was used for each qPCR triplicate reaction. The RealTime Ready Single Assays designed and LightCycler® 480 Probes Master mix used to perform qPCR experiments. Reactions set up in 384-well plates and run in the Roche LightCycler® 480 Instrument. The results were exported as text files from the Roche LightCycler® 480 Software. Expression values of target genes were normalized to *GAPDH* endogenous control [17]. Relative gene expression of *NPPB*, *ANGPTL2*, *IGFBP2*, *ATP1B4*, *MCOLN3*, *CACNB2*, and *BMP7* genes between AFib and SR groups was calculated using 2^-∆∆Ct^ method [18]. The ratio of AFib/SR tissues was calculated as the fold-change in expression.

### 2.7. Transmission Electron Microscopy (TEM)

Patients included in the TEM analysis for SR and AFib groups were matched for age and gender. Atrial tissues were fixed in 2% (*w*/*v*) paraformaldehyde and 2.5% (*v*/*v*) glutaraldehyde including 0.2 M phosphate buffer for 3 hours. All samples washed three times with 0.1 M phosphate buffer and then postfixed with 0.1 M phosphate buffer containing 1% osmium tetroxide for 1 hour under room temperature. After fixation, samples were dehydrated using a graded series of ethanol. Following dehydration, samples were embedded in Araldite CY 212. After 1 *μ*m semithin sections of specimens were stained with 1% toluidine blue, investigation areas on specimens were selected. Using Leica Ultracut R microtome 60-80, nanometer ultrathin sections were cut from selected areas. All ultrathin sections were stained with uranyl acetate and lead citrate. Ultrathin sections were investigated and photographed using LEO 906 E (80 kV—Oberkohen, Germany).

### 2.8. Statistical Analysis

Continuous variables were presented as mean ± standard deviation or median (interquartile range (IQR)) and categorical variables as percentages. Statistical analysis was carried out using paired student *t*-test for comparison between two groups. Pearson's Chi^2^ and Fisher's exact tests were used for categorical data. *P* values less than 0.05 were considered to be statistically significant. SPSS 21.0 for Mac (IBM SPSS Statistics, Chicago, Illinois) was used for statistical analyses.

We identified differentially expressed genes concerning the 1.5-fold change threshold. Mann–Whitney *U* test was applied to compare the qPCR gene expression values between AFib and SR groups. *P* values are adjusted using the Benjamini-Hochberg procedure. QPCR analysis was conducted using the TURCOSA (Turcosa Analytics Ltd. Co., Turkey, http://www.turcosa.com.tr) statistical software. False discovery rates (FDR) less than 10% were considered as statistically significant.

## 3. Results

### 3.1. Clinical Data

We isolated RNA from 62 atrial tissues. Among 62 tissue samples, RNA quality control was unsuccessful in 3 atrial samples (one LA sample from the SR group and one RA and one LA samples from the AFib group) to continue microarray analysis (Figure 1). Among 59 human atrial samples analyzed, 8 pairs of LA and RA tissues were evaluated by final qPCR analysis. We did microarray analysis on 59 samples (*n* = 59) and qPCR validation experiments on 8 paired samples (*n* = 16).

Patient demographics are shown in Table 1. The groups were matched for age, sex, body surface area, smoking history, the incidence of diabetes mellitus, and left ventricular ejection fraction. At baseline, clinically significant differences between the AFib and SR groups were LA size and severe tricuspid regurgitation. The mean duration of AFib was 720.3 ± 223.3 days (range: 370-1080 days) in the AFib group.

### 3.2. Transcriptomic Signatures of Atrial Fibrillation

The results of this study expose that several genes are involved in persistent AFib. Stepwise comparisons were performed using LA only, RA only, and RA + LA tissues together. Numbers of differentially expressed genes between AFib and SR groups (adj *P* < 0.05 and minimum fold change ± 1.5) were listed in Table 2. Of those 178 genes, 88 were upregulated, and 90 were downregulated in both RA and LA tissues with AFib compared to the SR group (Supp. Table-6). Because AFib is a disease that starts from the left atrium and then affects both atrial chambers as AFib progresses towards the permanent state, AFib influences atrial tissues, both temporarily and permanently.

There may be different molecular mechanisms involved in AFib in LA and RA tissues. We focused on gene expression changes in only RA (Supp. Table-7), only LA (Supp. Table-8), and RA + LA to evaluate how AFib develops in the molecular stage [19]. In our study, molecular aspects of transcriptomics give a foresight about the influences of persistent AFib on human atrial tissues. The most significant differentially expressed genes with their *P* values, *q* values, and fold changes are listed in Table 3.

Functional enrichment analysis of DEGs between AFib vs. SR from samples of only RA, only LA, and RA + LA tissues showed that several GO terms and KEGG pathways were commonly enriched. GO:0043062 extracellular structure organization, GO:0009612 response to mechanical stimulus, GO:0007178 transmembrane receptor protein serine/threonine kinase signaling pathway, GO:0060537 muscle tissue development, GO:0003012 muscle system process, and GO:0001505 regulation of trans-synaptic signaling were enriched GO terms of DEGs between AFib vs. SR in all 3 atrial tissues (RA/LA/RA + LA). Similarly, hsa04974 protein digestion and absorption, hsa04512 ECM-receptor interaction, hsa04510 focal adhesion, and hsa04151 PI3K-Akt signaling pathways were come into prominence in KEGG pathway analysis. Additionally, hsa04010 MAPK signaling pathway and hsa04933 AGE-RAGE signaling pathway in diabetic complications in KEGG pathways were upregulated between AFib vs. SR groups of only LA and only RA atrial tissues.

**Figure 3:**
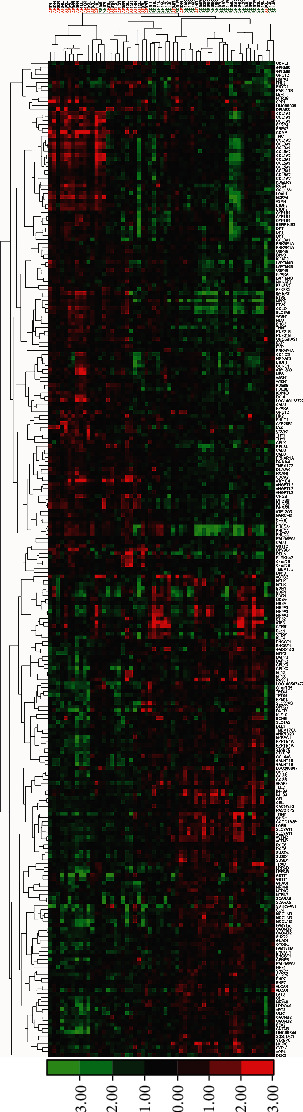
Hierarchical cluster analysis of gene expression microarrays of right and left atrial biopsies from AFib patients with primary mitral regurgitation compared to controls (S.R.). Heatmap shows the clusters how 178 differentially expressed genes separate all samples using 1.5-fold change as the threshold. Consequential 178 differentially expressed genes outcome from comparing gene expression profiles between AFib vs. S.R. of all atria tissues and separate AFib samples from S.R. samples with 85% success. Columns represent samples, and rows represent differentially expressed genes. As can be seen on the color bar, red indicates increased gene expression, and green indicates decreased gene expression. Heat and intensity of red or green color appear according to the fold-change.

Heatmap of gene expression between AFib and SR groups uses differentially expressed genes in the intersection of only RA, only LA, and RA + LA tissues. In the unsupervised hierarchical cluster analysis, DEGs distinguish 85% of AFib from SR samples and 74% of SR from AF samples (Figure 3).

### 3.3. Meta-Analysis

We have performed bioinformatics on Partek Genomics Suite of GSE41177 and GSE2240 raw data using all cel files according to the comparison of AFib versus SR with the same analysis algorithm we applied for our raw data (GSE115574). We used Venn diagrams to compare the DEGs of GSE41177 (Supp. Table-3) and GSE2240 (Supp. Table-4) studies and DEGs of our study (Supp. Table-5). We noticed that microarray results of GSE2240 were more similar to our data than GSE441177 in terms of the DEGs. According to functional enrichment analysis, GSE2240 data showed upregulation in “Focal adhesion” and “PI3K-Akt signaling pathway” KEGG pathways common with our results common in AFib vs. SR comparisons in on LA, only RA, and LA + RA tissues. “MAPK signaling pathway” is another enriched pathway of GSE2240 data which is common with our AFib vs. SR data revealed from only RA tissues. “ECM-receptor interaction” pathway is also a common enriched pathway overlapped with GSE2240 and our data from RA and RA + LA tissues.

Pathway analysis of GSE41177 data was not match up with both our dataset and GSE2240. DEGs were enriched in “hsa05150: Staphylococcus aureus infection” (FDR = 2.19*E* − 04), “hsa05140: Leishmaniasis” (FDR = 2.43*E* − 04), and hsa04145: Phagosome (FDR = 1.12*E* − 06) utmost.

For microarray analysis validation, genes were chosen from among the most differentially expressed genes of our data and convergent DEGs in meta-analysis and our data. Several genes were not on the same direction as up- or downregulation. Through genes of interest, nine genes (NPPB, ANGPTL2, COLQ, IGFBP2, BMP7, COMP, DNAJA4, DHRS9, CHGB) were parallel with microarray and statistically significant (Supp. Table-9).

### 3.4. TEM

**Figure 4:**
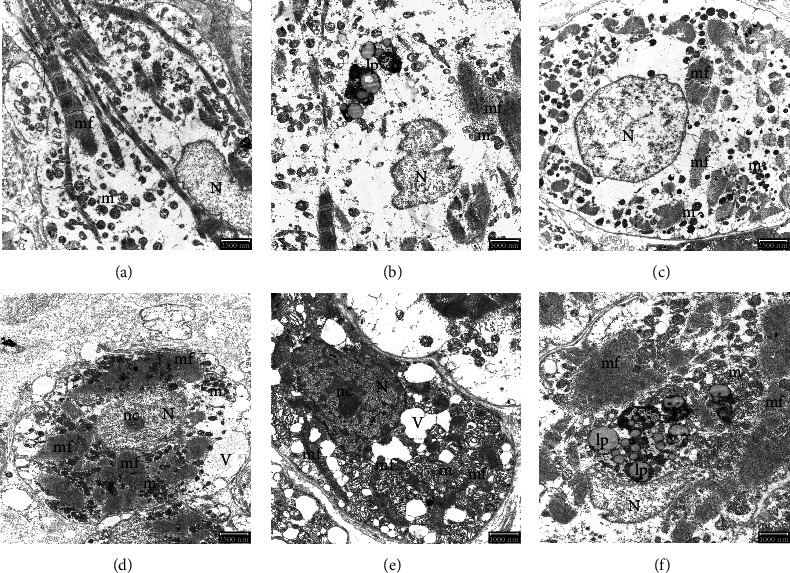
Electron micrograph of human atrial tissues. (a) Normal nucleus, typical striated myofilaments, and scattered mitochondria in a longitudinal section of the SR atrial sample. Scale bar: 2500 nm. (b) Lipofuscin accumulation, striated myofibrils, and mitochondria in the longitudinal section of the AFib atrial sample. Scale bar: 1000 nm. (c) Cross-section of the cardiac cell of SR atrial sample mitochondria and myofilaments is arranged around the nucleus. Scale bar: 2500 nm. (d) At the same magnification of the cardiac cell of AFib atrial sample in cross-section, shrinkage of the cell. Scale bar: 2500 nm. (e) Large vacuoles, myofilaments, and mitochondria clusters in condensed cytoplasm in AFib atrial sample. Scale bar: 1000 nm. (f) Lipofuscin accumulations gathered mitochondria and myofilaments in shrinkage cells in AFib atrial sample. Scale bar: 1000 nm. N: nucleus; nc: nucleolus; V: vacuole; lp: lipofuscin accumulations; m: mitochondria; mf: myofilaments.

The centrally located nucleus has convoluted membranes, nucleolus, and a typical periodic striated microfilament pattern, and evenly distributed mitochondria can be seen in SR atrial tissues (Figures 4(a) and 4(c)). On the contrary, we observed shrinkage in the size of some atrial cardiomyocytes. The accumulation of mitochondria, myofilaments, and membrane-bound large vacuoles was also prominent in AFib atrial tissues. However, the most striking feature was the excessive lipofuscin deposits in AFib samples compared to SR samples (Figures 4(b) and 4(d)–4(f)).

## 4. Discussion

**Figure 5:**
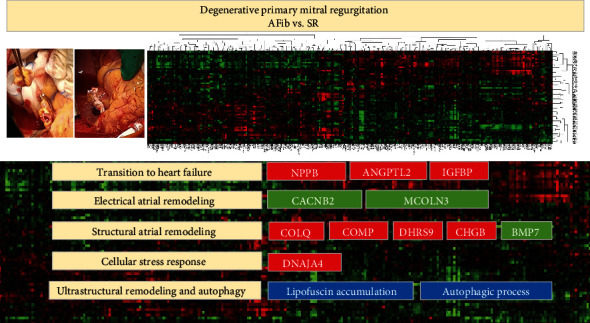
Differentially expressed genes in LAA tissues of AFib vs. SR (fold change > 1.5; *P* < 0.05) and ultrastructural changes. As can be seen on the color bar, red indicates increased gene expression, and green indicates decreased gene expression.

Over the last decade, the field of AFib genomics has been extensively investigated through animal models and subsequent human population studies. Previous proteomic analysis from LA and RA samples comparing changes in the expression levels of proteins between SR and AFib and mitral valve disease showed different levels of proteins associated with the cytoskeleton, energetic metabolism, and cardiac cytoprotection [20]. This study presents several novel characteristics. First, we evaluated the transcriptional changes in both human left and right atrial tissues in a homogenous target population involving chronic degenerative primary mitral regurgitation. Second, our comparative analysis revealed differentially regulated genes and pathways in persistent AFib compared to SR in both LA and RA samples. These mechanisms and pathways imply cardiac remodeling overall, including molecular changes of transition to heart failure, electrical and structural remodeling, and cellular stress response. In brief, AFib persistence was associated with the upregulation of *NPPB*, *ANGPTL2*, *IGFBP2*, *COLQ*, *COMP*, *DNAJA4*, *DHRS9*, and *CHGB*. Furthermore, AFib persistence was associated with the downregulation of *CACNB2*, *MCOLN3*, and *BMP7* (Figure 5).

Our results showed that the most relevant gene expression change was the NPPB gene between AFib and SR groups in both LA and RA tissue. NPPB elevation reflects atrial pressure and mechanical stretching of the atria. Natriuretic peptides function as cardiac neurohormones, which are secreted mainly in ventricles and function to reduce circulating volume, cardiac output, and systemic blood pressure and increases the risk of AFib. The plasma NPPB level is widely used in clinical practice to predict heart failure and AFib. Cardiomyocytes secrete natriuretic peptides in response to a few stimuli; one of them is increased wall stress [21]. Studies demonstrated that B-type natriuretic peptide concentration in plasma is significantly reduced after catheter ablation procedures in patients with AFib and plasma NPPB level elevation after ablation are associated with AFib recurrence [22].

Furthermore, angiopoietin-like protein 2 (*ANGPTL2*) is a secreted glycoprotein with homology to the angiopoietins and may exert a function on endothelial cells through autocrine or paracrine action. Circulating *ANGPTL2* levels positively correlate with cardiac dysfunction in patients with dilated cardiomyopathy.

Previous proteomics studies have indicated a negative correlation between *IGFBP2* levels and LVEF and proposed *IGFBP2* as a candidate diagnostic biomarker for heart failure [23] and a strong prognostic factor for cardiovascular mortality. *IGFBP2* upregulation observed in our study confirms these findings.

Alterations in the regulation of genes encoding ion channels, pumps, exchangers, gap junction proteins, and signaling molecules may lead to electrical remodeling in atrial cardiomyocytes. They can contribute to AFib initiation and persistence [5]. L-type Ca^2+^ current and inward rectifier K^+^ currents and gap junction connexin hemichannels have been confirmed for electrical remodeling in AFib [5, 8]. Calcium voltage-gated channel auxiliary subunit beta 2 (*CACNB2*) is one of the four homologous genes, which are essential modulators of the L-type calcium channel activity. We found that *CACNB2* was significantly downregulated in atrial tissues from patients with persistent AFib, which confirms Ca^2+^ handling impairment during electrical atrial remodeling as previously reported in chronic Afib [24, 25] and dilated cardiomyopathy [26]. However, others indicated that *CACNB2* expression is upregulated in the transgenic mouse model of failing myocardium [27].

Transient receptor potential channels, also known as mucolipin subfamily, are highly expressed in cardiac fibroblasts. They consist of several nonselective cation channels. *MCOLN3* (TRPML3) gene runs as an inward rectifying Ca^2+^ cation channel and intervenes in releasing Ca^2+^ from endosomes to the cytoplasm. *MCOLN3* activity downregulation, as observed in our study, correlates with clear congestion of luminal Ca^2+^ in endosomes, and this congestion may lead to rough defects caused by endosomal acidification [28]. *MCOLN3* also plays an essential role in the regulation of Ca^2+^ trafficking along with the autophagosome maturation.

Previous studies confirmed the presence of structural remodeling, notably atrial enlargement and fibrosis in persistent AFib [6]. Fibroblast-cardiomyocyte interactions increased collagen content, and altered compositions of extracellular matrix proteins are the hallmarks of structural remodeling in AFib [5]. Bone morphogenetic proteins (BMPs) are members of the transforming growth factor *β* superfamily. They play a critical role in the cardiac formation and the inducers of cardiac differentiation. In a persistent AFib setting, we identified the downregulation of *BMP7*, which may alter conduction patterns and increase vulnerability to reentry in atria.

Furthermore, the upregulation of *COLQ* (collagen like tail subunit of asymmetric acetylcholinesterase), *COMP* (cartilage oligomeric matrix protein), and *CHGB* (cartilage oligomeric matrix protein, chromogranin B, secretogranin B) observed in this study may contribute to atrial fibrosis and structural remodeling but require further investigation.

Autophagy is a dynamic, physiological cellular process that dysfunctional intracellular components are self-digested by lysosomes [29, 30]. Excessive activation of autophagy can lead to cell death [29, 30]. Previous electron microscopic study by Garcia et al. [29] showed impaired autophagy in the atria of patients who developed postoperative AFib in patients in sinus rhythm who had undergone elective coronary artery bypass grafting. This novel evidence suggests that ultrastructural atrial remodeling characterized by impaired cardiac autophagy is associated with the establishment of a proarrhythmic substrate [29]. Our TEM results in AFib atrial samples revealed cardiomyocyte vacuolization and nuclear derangement of myocytes. As known, residual bodies like lipofuscin are the oldest lysosomes and are thought to be the remnant of lysosomal activity. They show the completed digestive functions of long-lived cells such as cardiac muscle cells and are packed with debris and indigestible material. These findings and lipofuscin accumulation suggest an excessive autophagic process in response to fibrillation stimuli [31]. They have not during AFib progression, autophagy may also contribute to electrical remodeling in the atrium, together with ubiquitination and degradation of Cav1.2 (L-type calcium channel, voltage-dependent, a 1-C subunit). The NPPB, COMP, RELN, TNC, RCAN1, AQP4, and GADD45G genes which all were seemed to be expressed differently in the study are related with autophagic pathways directly or indirectly, so we believe that further studies are required to evaluate these pathways in detail in the future.

The expression of heat shock proteins (HSPs) results from cellular stress that enables the heat shock transcription factor-1 (HSF-1) by perceiving. HSPs are aimed at protecting cells from metabolic and thermal stresses. *DNAJA4* upregulation, a member of the HSP family detected in this study, is consistent with *in vitro* and *in vivo* AFib experimental models [32]. HSP induction against AFib persistence warrants further investigations.

## 5. Limitations

Analysis of the differentially expressed genes between AFib and SR groups and data interpretation was challenging. Firstly, we are aware that we have not worked with the entire target population due to financial strains, and our results can be affected by random error (or sampling error). Secondly, gene expression profiling is a perspective of genome-wide mRNA expression. Altered translation, functional protein structures, and protein-protein interactions, regulatory processes cannot be anticipated in gene expression studies. Thirdly, modern imaging methods to detect and quantify atrial fibrosis are missing in this study. Fourthly, studying human tissue is one of the significant limitations. The atrial pressure/volume stressors of chronic mitral insufficiency could have been the stimulus for the gene expression profiles and molecular level changes. However, we tried to homogenize the study group by selecting the patients with degenerative mitral valve disease and compared AFib and SR groups to diminish other variables' possible effects. Indeed, both groups represent different stages of the same disease. The loss of atrial contraction also leads to blood stasis in the atria in the AFib group and may lead to higher pulmonary artery pressures, larger-sized LA, and advanced TR than the controls. Moreover, there is no possibility of finding a similar study in the literature; having the homogenized human groups may lead to a false comparison of our results with different studies. Finally, our study population consists of only Turkish ancestry from a single-center, which may not reflect the ethnic diversity of AFib. Thus, this data should be handled with caution until confirmed with other patient populations.

Another limitation of the study was limited sample size of groups because of clinical conditions that had potential effects on molecular results that were excluded.

## 6. Conclusion

The growing number of solved molecular signatures of AFib provides a framework to now interpret persistent AFib mechanisms. Understanding these complex mechanisms will allow for personalized management of AFib. Furthermore, differential proteins and metabolites may lead to identifying biomarkers to predict AFib persistence postoperatively [33]. Our comparative analysis reveals that atrial cardiomyocyte remodeling in persistent AFib was closely linked to alterations in gene expression profiles compared to SR in patients with primary MR. Our findings may provide novel therapeutic strategies targeted to cardiac remodeling in persistent AFib. Presented different expression patterns of genes in AFib may lead to novel biomarkers, which could also serve as potential therapeutic targets for treating Afib. Furthermore, the utilization of the proteins predicting the AFib in the blood warrants further intensive research.

## Figures and Tables

**Table 1:** Baseline patient demographics and hemodynamics.

Characteristic	Afib (*n* = 15)	SR (*n* = 16)	*P* value
Age, years, mean ± SD (min-max)	67.0 ± 13.0 (28-80)	59.3 ± 11.6 (33-82)	0.098
Sex, male, *n* (%)	4 (26.6)	5 (33.3)	0.8
BSA, m^2^, mean ± SD (min-max)	1.81 ± 0.17 (1.39-2.05)	1.87 ± 0.17 (1.66-2.31)	0.341
Medical history			
Smoking, *n* (%)	7 (46.6)	8 (53.3)	0.9
COPD, *n* (%)	5 (33.3)	4 (26.6)	0.8
Diabetes mellitus, *n* (%)	3 (20)	4 (26.6)	0.20
CVD, *n* (%)	4 (26.6)	1 (6.6)	0.134
Hypertension, *n* (%)	11 (73.3)	4 (26.6)	0.015^★^
Hyperlipidemia, *n* (%)	8 (53.3)	5 (33.3)	0.148
Total cholesterol, mg/dl, mean ± SD	185.8 ± 7.1	185.8 ± 7.6	0.999
LDL cholesterol, mg/dl, mean ± SD	116.5 ± 5.6	115.7 ± 6.3	0.93
HDL cholesterol, mg/dl, mean ± SD	44.4 ± 4.2	39.5 ± 1.9	0.35
LVEDD, mm, mean ± SD	54.6 ± 6.9	54.3 ± 12	0.927
LVESD, mm, mean ± SD	34.7 ± 6.2	35.5 ± 9.4	0.748
DBP, mmHg, mean ± SD	86.8 ± 11.4	89 ± 9.6	0.07
LVEF, %, mean ± SD	52.9 ± 8.9	53.0 ± 7.1	0.964
Systolic PAP, mmHg, mean ± SD	54.7 ± 9	43 ± 16	0.008^★^
LA diameter, mm, mean ± SD	55.9 ± 6.2	47.7 ± 6.3	<0.0001^★^
NYHA, > class 2, *n* (%)	10 (66.6)	9 (60)	0.3
Tricuspid regurgitation, > 2, *n* (%)	6 (40)	2 (13.3)	<0.0001^★^
Euroscore, mean ± SD	5.8 ± 3.5	3.8 ± 2.4	0.051
Logistic Euroscore, %, mean ± SD	8.2 ± 8.1	4.1 ± 4.8	0.07
Medications			
Antihyperlipidemic, *n* (%)	7 (46.6)	7 (46.6)	1
Digitalis, *n* (%)	5 (33.3)	2 (13.3)	0.184
Calcium channel blocker, *n* (%)	5 (33.3)	0 (0)	0.014^★^
Beta blocker, *n* (%)	11 (73.3)	10 (66.6)	0.621
ACE/ARB inhibitors, *n* (%)	9 (60)	8 (53.3)	0.224

Data presented as mean ± SD. ^∗^*P* < 0.05 Student's *t*-test for paired comparisons. ^★^ indicates significantly different *P* values. AFib: atrial fibrillation; SR: sinus rhythm; BSA: body surface area; COPD: chronic obstructive pulmonary disease; CVD: cerebrovascular disease; LVEDD: left ventricular end-diastolic diameter; LVESD: left ventricular end-systolic diameter; DBP: diastolic blood pressure; LVEF: left ventricular ejection fraction; PAP: pulmonary artery pressure; LA: left atrium; NYHA: New York Heart Association; ACE: angiotensin-converting enzyme; ARB: angiotensin II receptor blockers.

**Table 2:** Number of differentially expressed genes; fold change ± 1.5; *P* < 0.05.

Genes	Atrial fibrillation versus sinus rhythm		
	RA + LA AFib vs. SR	RA AFib vs. SR	LA AFib vs. SR
Number of DEGs	178	232	209
Increased expression	88	97	126
Decreased expression	90	135	83

RA: right atrium; LA: left atrium; DEGs: differentially expressed genes.

**Table 3:** The most significant differentially expressed genes with their fold changes, *P* values, and *q* values.

Probeset ID	Gene symbol	FC RA + LA AFib vs. SR	*P* value	*q* value (adjusted *P* value)	FC RA AFib vs. SR	*P* value	*q* value (adjusted *P* value)	FC LA AFib vs. SR	*P* value	*q* value (adjusted *P* value)
206801_at	NPPB	3.46	4.16*E*-04	8.65*E*-04	4.45	0.002	0.005	2.68	0.037	0.042
243737_at	ATP1B4	2.81	2.82*E*-05	1.05*E*-04	2.90	7.25*E*-04	0.003	2.87	0.006	0.01
206073_at	COLQ	2.64	1.15*E*-05	5.53*E*-05	3.27	8.75*E*-05	0.001	2.08	0.009	0.02
202718_at	IGFBP2	2.61	2.07*E*-04	4.87*E*-04	2.56	0.015	0.018	2.64	0.001	0.0072
205713_s_at	COMP	2.47	7.52*E*-05	2.30*E*-04	2.12	0.013	0.016	2.64	0.005	0.015
228754_at	SLC6A6	2.4	1.18*E*-06	1.25*E*-05	2.86	5.48*E*-05	7.70*E*-04	2.01	0.003	0.012
204260_at	CHGB	2.37	1.62*E*-08	1.71*E*-06	2.34	2.51*E*-05	5.66*E*-04	2.38	2.67*E*-04	0.005
224009_x_at	DHRS9	2.24	1.72*E*-06	1.56*E*-05	2.44	5.29*E*-05	7.70*E*-04	1.99	0.011	0.021
205923_at	RELN	2.19	0.001	0.002	2.29	0.018	0.021	2.10	0.036	0.042
213001_at	ANGPTL2	2.09	6.74*E*-08	2.13*E*-06	2.07	3.83*E*-06	2.00*E*-04	2.12	0.001	0.008
201645_at	TNC	1.84	0.002	0.003	1.69	0.0284	0.031	1.94	0.046	0.046
206768_at	RPL3L	1.93	1.16*E*-04	3.14*E*-04	2.24	3.74*E*-04	0.002	1.63	0.003	0.0122
205493_s_at	DPYSL4	1.9	6.23*E*-04	0.001	1.77	0.029291	0.0314	2.04	0.008	0.018
208370_s_at	RCAN1	1.86	7.78*E*-07	8.60*E*-06	2.05	3.93*E*-05	7.66*E*-04	1.66	0.006	0.0169
225061_at	DNAJA4	1.75	6.75*E*-07	7.85*E*-06	2.09	3.40*E*-07	9.95*E*-05	^∗^	^∗^	^∗^
229797_at	MCOLN3	-2.81	2.42*E*-08	1.07*E*-06	-2.43	4.71*E*-05	7.70*E*-04	-3.14	2.42*E*-05	9.98*E*-04
222927_s_at	CPLX3	-2.43	4.15*E*-05	1.45*E*-04	-2.94	0.002	0.005	-1.98	0.005	0.0167
205177_at	TNNI1	-2.43	3.94*E*-04	8.28*E*-04	-3.21	0.002	0.005	-1.86	0.027	0.0366
207344_at	AKAP3	-2.39	1.76*E*-06	1.56*E*-05	-1.99	0.008	0.01	-2.80	7.41*E*-05	0.002
222919_at	TRDN	-2.12	6.09*E*-08	2.13*E*-06	-1.91	5.62*E*-05	7.70*E*-04	-2.28	5.71*E*-04	0.0055
209693_at	ASTN2	-1.97	1.45*E*-04	3.56*E*-04	-2.23	0.001	0.004	-1.50	0.009	0.02
226228_at	AQP4	-1.85	6.82*E*-05	2.18*E*-04	-2.15	0.00180054	0.0050	-1.5	0.007	0.018
205910_s_at	CEL	-1.81	8.54*E*-05	2.48*E*-04	-1.99	0.003	0.006	-1.62	0.005	0.015
221288_at	GPR22	-1.78	2.56*E*-06	2.02*E*-05	-1.87	9.17*E*-05	0.001	-1.66	0.007	0.018
204121_at	GADD45G	-1.75	6.56*E*-04	0.001	-1.71	0.027	0.029	-1.82	0.009	0.0195
1559419_at	CACNB2	-1.70	6.83*E*-04	0.001	-1.72	0.004	0.007	-1.60	0.035	0.0416
214369_s_at	RASGRP2	-1.60	1.11*E*-07	2.87*E*-06	-1.65	1.97*E*-04	0.0016	-1.55	2.31*E*-04	0.0032
209590_at	BMP7	-1.57	6.39*E*-06	3.82*E*-05	-1.58	7.51*E*-04	0.0033	-1.54	8.67*E*-04	0.0066
221796_at	NTRK2	-1.57	1.32*E*-04	3.37*E*-04	-2.05	9.54*E*-07	9.95*E*-05	^∗^	^∗^	^∗^
213992_at	COL4A6	-1.51	0.005	0.006	-1.53	0.012	0.015	-1.53	0.022	0.032

The most significant 30 genes are chosen according to fold changes. Coexistent transcript Affy IDs in three groups of tissue types (RA + LA, RA, LA) are considered the utmost. Genes in the table were selected, the top 15 of the lists that comparison between AFib and SR (FC ± 1.5) in RA + LA, RA, and LA tissues. Some Affy IDs mentioned with ^∗^ are not found in all three lists. However, a connection found between that gene and AFib in other studies. RA: right atrium; LA: left atrium; AFib: atrial fibrillation; SR: sinus rhythm; FC: fold change. Presented genes in alphabetical order: AKAP3: A-kinase anchoring protein 3; ANGPTL2: angiopoietin like 2; AQP4: aquaporin 4; ASTN2: astrotactin 2; ATP1B4: ATPase Na+/K+ transporting family member beta 4; BMP7: bone morphogenetic protein 7; CACNB2: calcium voltage-gated channel auxiliary subunit beta 2; CEL: carboxyl ester lipase; CHGB: chromogranin B; COL4A6: collagen type IV alpha 6 chain; COLQ: collagen like tail subunit of asymmetric acetylcholinesterase; COMP: cartilage oligomeric matrix protein; CPLX3: complexin 3; DHRS9: dehydrogenase/reductase 9; DNAJA4: DnaJ heat shock protein family (Hsp40) member A4; DPYSL4: dihydropyrimidinase like 4; GADD45G: growth arrest and DNA damage inducible gamma; GPR22: G protein-coupled receptor 22; IGFBP2: insulin like growth factor binding protein 2; MCOLN3: mucolipin TRP cation channel 3; NPPB: natriuretic peptide B; NTRK2: neurotrophic receptor tyrosine kinase 2; RASGRP2: RAS guanyl releasing protein 2; RCAN1: regulator of calcineurin 1; RELN: reelin; RPL3L: ribosomal protein L3 like; SLC6A6: solute carrier family 6 member 6; TNC: tenascin C; TNNI1: troponin I1, slow skeletal type; TRDN: triadin.

## Data Availability

This trial is registered with ClinicalTrials.gov identifier: NCT00970034. We also submitted the dataset of gene expression microarrays at GEO (Gene Expression Omnibus) with accession number GSE115574 (https://www.ncbi.nlm.nih.gov/geo/query/acc.cgi?acc=GSE115574).

## Abbreviations

AFib: Atrial fibrillation
ANGPTL2: Angiopoietin like 2, angiopoietin-related protein 2
BMP7: Bone morphogenetic protein 7
CACNB2: Calcium voltage-gated channel auxiliary subunit beta 2
CHGB: Cartilage oligomeric matrix protein, chromogranin B, secretogranin B
COLQ: Collagen like tail subunit of asymmetric acetylcholinesterase
COMP: Cartilage oligomeric matrix protein
DHRS9: Dehydrogenase/reductase 9
DNAJA4: DnaJ heat shock protein family (Hsp40) member A4
FDR: False discovery rate
FC: Fold change
GEO: Gene expression omnibus
IGFBP2: Insulin-like growth factor binding protein 2
KEGG: Kyoto Encyclopedia of Genes and Genomes
LA: Left atrial
LVEF: Left ventricular ejection fraction
MCOLN3: Mucolipin 3, transient receptor potential channel mucolipin 3
MR: Mitral regurgitation
NPPB: Natriuretic peptide B, gamma-brain natriuretic peptide, natriuretic peptide precursor B, brain type natriuretic peptide
NYHA: New York Heart Association
PCR: Polymerase chain reaction
RA: Right atrial
SR: Sinus rhythm.

## Supplementary Materials

Supplementary 1 Supplementary Figure 1: microdissection technique for intact total RNA isolation from human atrial tissues. We optimized tissue preparing process under sterile conditions. Adipose tissue discarded right after biopsy-sized atrial tissue harvested. Atrium tissue dissected into tiny pieces with scissors and clamp on ice. Dissected tissue is submerged into liquid nitrogen in operating theatre and then transferred to -80°C in the laboratory until RNA isolation.

Supplementary 2 Supplementary Table 1: transcriptional profiling studies of AFib in different tissues and organisms.

Supplementary 3 Supplementary Table 2: samples that chosen for meta-analysis.

Supplementary 4 Supplementary Table 3: all differentially expressed genes in all LA-PV and LAA tissues of AFib vs. SR using GSE41177 data set (fold change > 1.5; *P* < 0.001 and *q* < 0.05).

Supplementary 5 Supplementary Table 4: all differentially expressed genes in all LAA tissues of AFib vs. SR using GSE2240 dataset (fold change > 1.5; *P* < 0.001 and *q* < 0.05).

Supplementary 6 Supplementary Table 5: Venn diagrams of meta-analysis and comparisons of DEGs from different tissues.

Supplementary 7 Supplementary Table 6: differentially expressed genes obtained from both LAA and RAA tissues of AFib vs. SR (fold change > 1.5; *P* < 0.05 and *q* < 0.05).

Supplementary 8 Supplementary Table 7: differentially expressed genes in RAA tissues of AFib vs. SR (fold change > 1.5; *P* < 0.05 and *q* < 0.05).

Supplementary 9 Supplementary Table 8: differentially expressed genes in LAA tissues of AFib vs. SR (fold change > 1.5; *P* < 0.05 and *q* < 0.05).

Supplementary 10 Supplementary Table 9: comparison of QPCR gene expression values between AFib vs. SR groups.

Supplementary 11 Supplementary Table 10: bioinformatics roadmap.
