# Supplementary figures and images for: Molecular Signatures of Human Chronic Atrial Fibrillation in Primary Mitral Regurgitation
Source: Cardiovasc Ther. 2021 Oct 15;2021:5516185. doi: 10.1155/2021/5516185 (PMC8538404; doi:10.1155/2021/5516185)

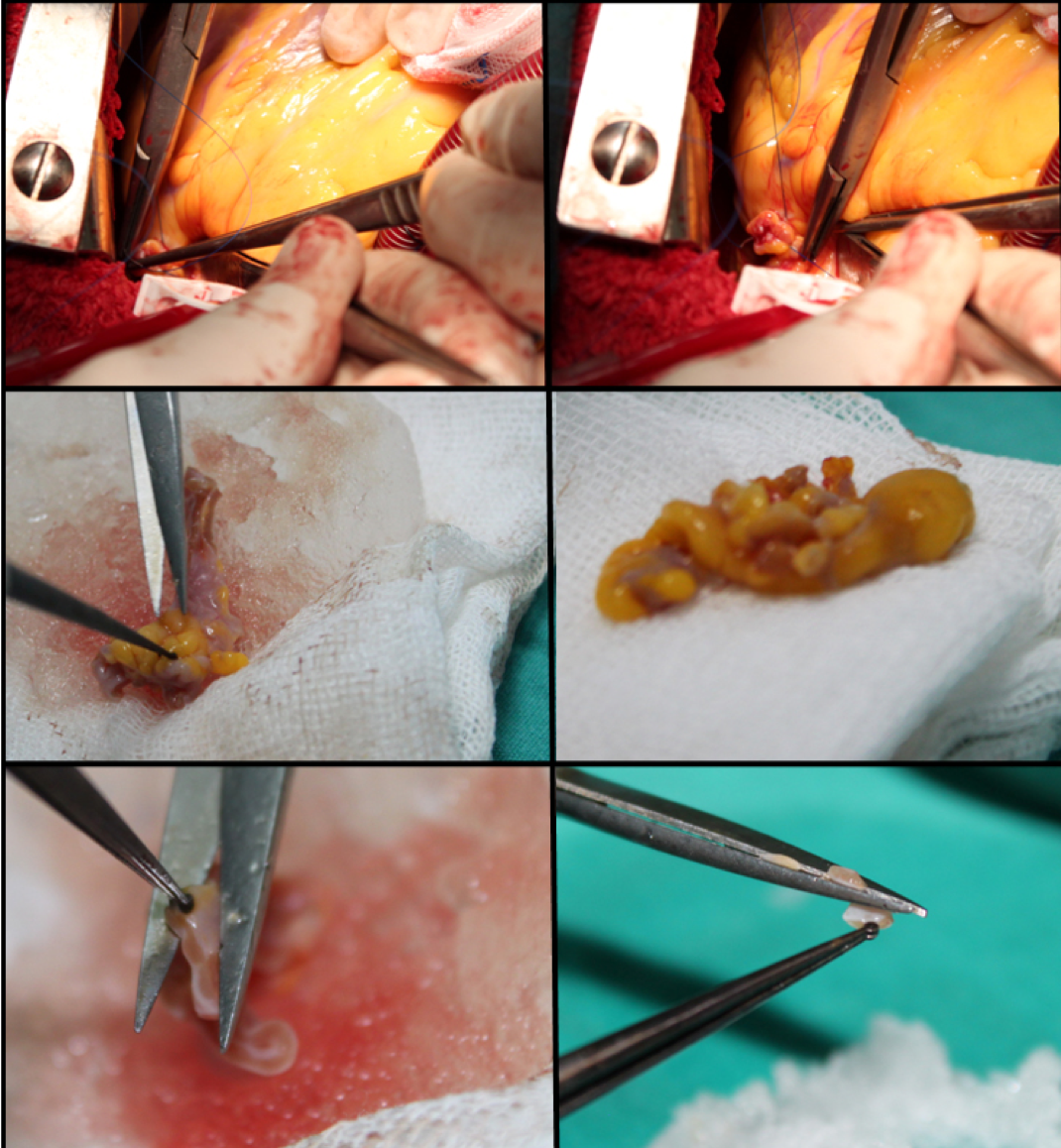

Supplement: Supplementary 1 — Supplementary Figure 1: microdissection technique for intact total RNA isolation from human atrial tissues. We optimized tissue preparing process under sterile conditions. Adipose tissue discarded right after biopsy-sized atrial tissue harvested. Atrium tissue dissected into tiny pieces with scissors and clamp on ice. Dissected tissue is submerged into liquid nitrogen in operating theatre and then transferred to -80°C in the laboratory until RNA isolation. [file 5516185.f1.png]
