# Supplementary material for: Molecular Signatures of Human Chronic Atrial Fibrillation in Primary Mitral Regurgitation
Source: Cardiovasc Ther. 2021 Oct 15;2021:5516185. doi: 10.1155/2021/5516185 (PMC8538404; doi:10.1155/2021/5516185)
Supplement: Supplementary 2 — Supplementary Table 1: transcriptional profiling studies of AFib in different tissues and organisms. [file 5516185.f2.docx]

**Supplementary Table 1:** Comparative analysis of AFib transcriptome studies.

| Organism | Race | Tissue Type | Operation/  AFib model | Duration of AFib | Number of Samples | Study Design | Underlying Heart Disease | Platform | Number of Genes Compared | Changed Genes in AFib | References |
| --- | --- | --- | --- | --- | --- | --- | --- | --- | --- | --- | --- |
| Rat  (2019) | Wistar Rats | left atria | Animal AFib model | 10 days | n_AFib_= 10  n_SR_= 10 | 10 days RAP vs. SR | continuous rapid pacing 15 Hz, 900 beats/min for 10 days | Affymetrix Rat Genome | 28514 transcripts | AF vs. non-AF  956 changed genes  395 down  561 up | ^1^ |
| Human  (2016) | Taiwanese | RAA, LAA | MV surgery, CABG | AFib patients with persistent AFib known more than 6 months | n_AFib_= 7  n_SR_= 6  paired LAA-RAA | differential LA to RA gene expression ratios between AFib and SR | rheumatic heart disease with significant mitral stenosis, coronary artery disease | Affymetrix HG_U133 Plus 2.0 Array | 54676 human expressed sequence tags | LA vs. RA  SR: 522  AFib: 462  significantly different LA/RA ratio between AFib and SR: 309 genes | ^2^ |
| Human (2016) | Swiss | blood | persistent AFib underwent ECV | non-self terminating episode lasting **>** 7 days | n_AFib_= 67  n_SR_= 67  paired smaples (before and after ECV) | 1–2 hours before and 4-6 weeks after successful ECV blood sampling | Persistent AFib | Illumina Human HT-12v4 BeadChip | 34694 unique genes using 47323 probes | SLC25A20 PDK4 ITGB5 DDX11L2 NAT8B TSC22D1 LOC732450 SF1 PLIN2 LRP5L | ^3^ |
| Human  (2015) | American | LAA | cardiac surgery  donor heart (n=2) | AFib-SR= 36 (8-75) months  AFib-AFib= 48 (12-96) months | n_AFib-AFib_= 129  n_AFib-SR_= 78  n_SR-SR_= 32 | AFib-AFib vs. SR-SR  AFib-AFib vs. AFib-SR  AFib-SR vs. SR-SR | Coronary artery disease, valve disease | Illumina Human HT-12 Expression BeadChip arrays | 49576 probe sets | AFib-AFib vs. SR-SR: 433 up 578 down  AFib-AFib vs. AFib-SR: 1043 up 1302 down  AFib-SR vs. SR-SR: 72 up 118 down | ^4^ |
| Canine  (2015) | Canis familiaris | anterior right fat pads | animal AFib model | 4 weeks | n_C_= 1  n_AFib_= 1  (3 samples pooled) | AFib vs. SR  (cardiac fat pads serve as intrinsic cardiac autonomic nerve source) | 400 bpm 4 weeks continuous RAP | Illumina Hiseq 2500  next-generation RNA sequencing | 61616 putative long non-coding RNAs | AFib vs. SR: 166 up 410 down | ^5^ |
| Human  (2014) | Large community based cohort | blood | The Framingham Heart Study Offspring Cohort | data is not available | n_AFib-Prevalent_= 177  n_AFib-Incident_= 143  n_SR_= 2126 | AFib-Prevalent vs. SR  AFib-Incident vs. SR  (Incident: 7 years follow up after blood collected) | large community-based cohort | Affymetrix Human Exon 1.0 ST Array | 287329 probe sets 209699 exons  17873 transcripts | AFib-Prevalent vs. SR: 7 genes up  AFib-Incident vs. SR: no significant changes | ^6^ |
| Human  (2013) | Taiwanese | LAA,  LA-PV junction | MV Replacement, AVR | 1-150 months | n_AFib-LAA_= 16  n_AFib-LA-PV_= 16  n_SR-LAA_= 3  n_SR-LAA-PV_= 3 | AFib vs. SR  paired LAA and LA-PV junction tissues from each patient | MR, MS, AS, ischemic heart disease | Affymetrix Human Genome U133 Plus 2.0 Array | 54765 human expressed sequence tags | LA-PV junction vs. LAA  AFib: 391 genes  SR: 332 genes  differentially expressed | ^7^  GSE41177 |
| Swine  (2013) | Sus scrofa | LAA | animal AFib model | 7 hours | n_AFib_= 3  n_AFib-I_= 5  n_SR_= 4 | SR vs. RAP  SR vs. RAP-Irbesartan | 600 bpm 7 hours RAP and RAP+Irbesartan | Affymetrix GeneChip Porcine Genome Arrays | 23937 probe sets  20201 genes | AFib vs. SR: 548 up 453 down  AFib-I vs. SR: 542 up 441 down | ^8^ |
| Swine  (2012) | Sus scrofa | RAA, LAA endothelial cells | animal AFib model | 6 weeks | n_AFib_= 4  n_C_= 4 | 6 weeks RAP persistent AFib model vs. SR | 600 bpm 6 weeks continuous atrial stimulation | Affymetrix GeneChip Porcine Genome Array | 23937 probe sets  20201 genes | LA: 325 genes  RA: 37 genes  differentially expressed | ^9^ |
| Swine  (2012) | Sinclair | LAA | animal AFib model, left lung lobectomy | 3 days | n_AFib_= 5  postop sustained AFib  n_SR_= 4 | lung lobectomy + AFib induced Swines vs. SR Swines | AFib induced by rapid burst pacing (10s) | Affymetrix GeneAtlas Porcine Gene 1.1 ST Array Strips | 25388 genes 572667 probes | 23 upregulated  10 downregulated genes (≥1.5-fold, *P*<0.05) in postop AFib group | ^10^ |
| Human  (2011) | Chinese | LAA | AFib: permanent AFib undergoing CABG + RA  C: healthy heart donors | 47.7 ± 25.0 months | n_AFib_= 7  n_C_= 4 | permanent AFib vs. SR | permanent AFib with coronary artery disease | Agilent Human 4x44K  Gene Expression Microarrays | 27958 genes | AFib vs. SR: 420 up 567 down | ^11^ |
| Mouse  (2009) | HL-1 atrial cell line | atrial cell line | cell culture AFib model | 24 hours | n_C_= 2  n_Paced_= 2  (5 samples pooled) | 24 hours RAP HL-1 cell culture AFib model vs. non-paced HL-1 cells | cells stimulated at 300 bpm for 24 hours | Affymetrix Genechip Mouse Genome 430 2.0 Array | 14000 mouse genes | 626 up 132 down | ^12^ |
| Swine  (2007) | Yorkshire-Landrace | LAA | animal AFib model | 3–4 weeks | n_AFib_= 9  n_SR_= 6 | 3-4 weeks continuous RAP at 600 bpm vs. SR | pacemaker 600 bpm 3-4 weeks | Incyte Human uniGEM V2 cDNA microarray | cross-species hybridization porcine RNA on human (9182 gene and EST) cDNA microarray | 121 genes upregulated 24 genes downregulated in AFib | ^13^ |
| Human (2006) | Russian | RAA | MV Replacement, CABG or LA myxoma excision | data is not available | n_C_= 10  n_AFib_= 12 | paroxysmal and constant AFib vs. SR (postmortem RAAs of street accidents) | CAD, MR, MS, LA myxoma | Atlas Human  cDNA Expression Arrays (Clontech) | 4100 genes | 24 up 15 down | ^14^ |
| Human  (2005) | French | RAA | MV Replacement, AVR, CABG | 7±4 years | n_AFib_= 11  n_SR_= 7  n_SR-CAD_= 11 | permanent AFib-VHD vs. SR-VHD or SR-CAD | MS, MR, AS, TR, ischemic heart disease | MWG Biotech human  genome-wide  microarray collection | 3863 genes | SR-VHD vs. SR-CAD: 832 genes  AFib-VHD vs. SR-VHD: 169 genes  differentially expressed | ^15^ |
| Human (2005) | Korean | LAA | AFib and C: MV Replacement  or AVR | more than 6 months | n_C_= 9  n_AFib_= 9 | chronic, persistent AFib vs. SR | MR, AR, MS | cDNA microarray | 8167 genes | 35 up 31 down | ^16^ |
| Human  (2005) | German | RAA, LV | AFib and C: Valve Repair or CABG  LV: nonfailing donor heart | Longer than 3 months | n_AFib_= 10  n_C_= 20  n_V_= 5 | permanent AFib atrium vs. SR atrium or LV | MR, MS, AR, AS, DC, CAD, Marfan | Affymetrix U133A+B Oligonucleotide Microarrays | 44.928 probe sets  33000 genes | 452 up 982 down | ^17^  GSE2240 |
| Swine  (2004) | Yorkshire-Landrace | RAA, LAA | animal AFib model | 6 weeks | n_C_= 6  n_AFib_= 6 | 6 weeks RAP AFib model vs. SR | pacemaker 600 bpm  4 weeks+ 2 weeks | cDNA microarray | 6035 genes | LA: 102 up 285 down  RA: 40 up 41 down | ^18^ |
| Human  (2003) | Korean | RAA | AFib: MV Repair + Maze  C: CABG | 7.6 years | n_C_= 26  n_AFib_= 26 | permanent AFib vs. SR | ASD, DC, HT | cDNA microarray | 1152 genes | 30 up 25 down | ^19^ |

AFib, atrial fibrillation; C, control; SR, sinus rhythm; RA, right atrium; LA, left atrium; RAA, right atrial appendage; LAA, left atrial appendage; A, atrial appendages; PV, pulmonary vein; MVR, mitral valve replacement; CABG, coronary artery bypass grafting; AVR, aortic valve replacement; LV, left ventricle; ASD, atrial septal defect; DC, dilated cardiomyopathy; HT, hypertension; AR, aortic regurgitation; AS, aortic stenosis; MR, mitral regurgitation; MS, mitral stenosis; CAD, coronary artery disease; VHD, valvular heart disease; TR, tricuspid regurgitation; PV, pulmonary vein; RAP, rapid atrial pacing; ECV, electrical cardioversion; RFA, radiofrequency ablation

1. Wang J, Li Z, Du J, Li J, Zhang Y, Liu J and Hou Y. The expression profile analysis of atrial mRNA in rats with atrial fibrillation: the role of IGF1 in atrial fibrosis. *BMC Cardiovasc Disord*. 2019;19:40.

2. Tsai FC, Lin YC, Chang SH, Chang GJ, Hsu YJ, Lin YM, Lee YS, Wang CL and Yeh YH. Differential left-to-right atria gene expression ratio in human sinus rhythm and atrial fibrillation: Implications for arrhythmogenesis and thrombogenesis. *International journal of cardiology*. 2016;222:104-112.

3. Raman K, Aeschbacher S, Bossard M, Hochgruber T, Zimmermann AJ, Kaufmann BA, Pumpol K, Rickenbacker P, Pare G and Conen D. Whole Blood Gene Expression Differentiates between Atrial Fibrillation and Sinus Rhythm after Cardioversion. *PLoS One*. 2016;11:e0157550.

4. Deshmukh A, Barnard J, Sun H, Newton D, Castel L, Pettersson G, Johnston D, Roselli E, Gillinov AM, McCurry K, Moravec C, Smith JD, Van Wagoner DR and Chung MK. Left atrial transcriptional changes associated with atrial fibrillation susceptibility and persistence. *Circ Arrhythm Electrophysiol*. 2015;8:32-41.

5. Wang W, Wang X, Zhang Y, Li Z, Xie X, Wang J, Gao M, Zhang S and Hou Y. Transcriptome analysis of canine cardiac fat pads: involvement of two novel long non-coding RNAs in atrial fibrillation neural remodeling. *J Cell Biochem*. 2015;116:809-21.

6. Lin H, Yin X, Lunetta KL, Dupuis J, McManus DD, Lubitz SA, Magnani JW, Joehanes R, Munson PJ, Larson MG, Levy D, Ellinor PT and Benjamin EJ. Whole blood gene expression and atrial fibrillation: the framingham heart study. *PloS one*. 2014;9:e96794.

7. Yeh YH, Kuo CT, Lee YS, Lin YM, Nattel S, Tsai FC and Chen WJ. Region-specific gene expression profiles in the left atria of patients with valvular atrial fibrillation. *Heart rhythm : the official journal of the Heart Rhythm Society*. 2013;10:383-91.

8. Chilukoti RK, Mostertz J, Bukowska A, Aderkast C, Felix SB, Busch M, Volker U, Goette A, Wolke C, Homuth G and Lendeckel U. Effects of irbesartan on gene expression revealed by transcriptome analysis of left atrial tissue in a porcine model of acute rapid pacing in vivo. *Int J Cardiol*. 2013;168:2100-8.

9. Cervero J, Segura V, Macias A, Gavira JJ, Montes R and Hermida J. Atrial fibrillation in Swines induces left atrial endocardial transcriptional remodelling. *Thromb Haemost*. 2012;108:742-9.

10. Heerdt PM, Kant R, Hu Z, Kanda VA, Christini DJ, Malhotra JK and Abbott GW. Transcriptomic analysis reveals atrial KCNE1 down-regulation following lung lobectomy. *J Mol Cell Cardiol*. 2012;53:350-3.

11. Zhou J, Gao J, Liu Y, Gu S, Zhang X, An X, Yan J, Xin Y and Su P. Human atrium transcript analysis of permanent atrial fibrillation. *Int Heart J*. 2014;55:71-7.

12. Mace LC, Yermalitskaya LV, Yi Y, Yang Z, Morgan AM and Murray KT. Transcriptional remodeling of rapidly stimulated HL-1 atrial myocytes exhibits concordance with human atrial fibrillation. *J Mol Cell Cardiol*. 2009;47:485-92.

13. Lin CS, Lai LP, Lin JL, Sun YL, Hsu CW, Chen CL, Mao SJ and Huang SK. Increased expression of extracellular matrix proteins in rapid atrial pacing-induced atrial fibrillation. *Heart Rhythm*. 2007;4:938-49.

14. Kharlap MS, Timofeeva AV, Goryunova LE, Khaspekov GL, Dzemeshkevich SL, Ruskin VV, Akchurin RS, Golitsyn SP and Beabealashvilli R. Atrial appendage transcriptional profile in patients with atrial fibrillation with structural heart diseases. *Ann N Y Acad Sci*. 2006;1091:205-17.

15. Lamirault G, Gaborit N, Le Meur N, Chevalier C, Lande G, Demolombe S, Escande D, Nattel S, Leger JJ and Steenman M. Gene expression profile associated with chronic atrial fibrillation and underlying valvular heart disease in man. *Journal of molecular and cellular cardiology*. 2006;40:173-84.

16. Kim NH, Ahn Y, Oh SK, Cho JK, Park HW, Kim YS, Hong MH, Nam KI, Park WJ, Jeong MH, Ahn BH, Choi JB, Kook H, Park JC, Jeong JW and Kang JC. Altered patterns of gene expression in response to chronic atrial fibrillation. *Int Heart J*. 2005;46:383-95.

17. Barth AS, Merk S, Arnoldi E, Zwermann L, Kloos P, Gebauer M, Steinmeyer K, Bleich M, Kaab S, Hinterseer M, Kartmann H, Kreuzer E, Dugas M, Steinbeck G and Nabauer M. Reprogramming of the human atrial transcriptome in permanent atrial fibrillation: expression of a ventricular-like genomic signature. *Circulation research*. 2005;96:1022-9.

18. Lai LP, Lin JL, Lin CS, Yeh HM, Tsay YG, Lee CF, Lee HH, Chang ZF, Hwang JJ, Su MJ, Tseng YZ and Huang SK. Functional genomic study on atrial fibrillation using cDNA microarray and two-dimensional protein electrophoresis techniques and identification of the myosin regulatory light chain isoform reprogramming in atrial fibrillation. *J Cardiovasc Electrophysiol*. 2004;15:214-23.

19. Kim YH, Lim DS, Lee JH, Shim WJ, Ro YM, Park GH, Becker KG, Cho-Chung YS and Kim MK. Gene expression profiling of oxidative stress on atrial fibrillation in humans. *Experimental & molecular medicine*. 2003;35:336-49.
