# Supplementary material for: Molecular Signatures of Human Chronic Atrial Fibrillation in Primary Mitral Regurgitation
Source: Cardiovasc Ther. 2021 Oct 15;2021:5516185. doi: 10.1155/2021/5516185 (PMC8538404; doi:10.1155/2021/5516185)
Supplement: Supplementary 3 — Supplementary Table 2: samples that chosen for meta-analysis. [file 5516185.f3.docx]

**Supplementary Table 2:** Comparative Analysis of Meta-analyses.

|  | GSE2240* | | GSE41177* | | | | GSE115574** | | | | | |
| --- | --- | --- | --- | --- | --- | --- | --- | --- | --- | --- | --- | --- |
| Rhythm | AFib | SR | AFib | | SR | | AFib | | | SR | | |
| Tissue Type | RA | RA | LA | LA-PV junction | LA | LA-PV junction | LA | RA | | LA | RA |  |
| Number of Samples | 10 | 20 | 16 | 16 | 3 | 3 | 15 | | 14 | 15 | 16 |  |

* Independent data set

** Our study’s data set
