# Supplementary material for: Molecular Signatures of Human Chronic Atrial Fibrillation in Primary Mitral Regurgitation
Source: Cardiovasc Ther. 2021 Oct 15;2021:5516185. doi: 10.1155/2021/5516185 (PMC8538404; doi:10.1155/2021/5516185)
Supplement: Supplementary 4 — Supplementary Table 3: all differentially expressed genes in all LA-PV and LAA tissues of AFib vs. SR using GSE41177 data set (fold change > 1.5; P < 0.001 and q < 0.05). [file 5516185.f4.docx]

**Supplementary Table 3:** All differentially expressed genes in all LA-PV and LAA tissues of AFib vs SR using GSE41177 data set (Fold Change > 1.5; p<0.001 and q<0.05).

| **Probeset ID** | **Entrez Gene** | **Gene Symbol** | **p-value** | **q value** | **MeanRatio(AF/SR)** | **MeanDiff(AF-SR)** | **FoldChange(AF/SR)** |
| --- | --- | --- | --- | --- | --- | --- | --- |
| 203536_s_at | 9391 | CIAO1 | 8,14E-08 | 1,99883E-05 | 1,72993 | 0,790714 | 1,72993 |
| 231966_at | 55607 | PPP1R9A | 1,07E-07 | 1,99883E-05 | 0,639082 | -0,645926 | -1,56474 |
| 231247_s_at | 727820 | LOC727820 | 1,17E-07 | 1,99883E-05 | 0,37365 | -1,42024 | -2,6763 |
| 210650_s_at | 27445 | PCLO | 1,64E-07 | 2,10319E-05 | 0,358712 | -1,4791 | -2,78775 |
| 212908_at | 23341 | DNAJC16 | 2,99E-07 | 2,78692E-05 | 0,587529 | -0,767268 | -1,70204 |
| 242819_at | --- | --- | 3,27E-07 | 2,78692E-05 | 0,431843 | -1,21142 | -2,31566 |
| 236331_at | 8999 | CDKL2 | 4,18E-07 | 3,05596E-05 | 0,578965 | -0,788452 | -1,72722 |
| 211792_s_at | 1031 | CDKN2C | 4,88E-07 | 3,12232E-05 | 1,6385 | 0,712372 | 1,6385 |
| 202867_s_at | 54788 | DNAJB12 | 1,43E-06 | 7,89969E-05 | 1,71426 | 0,77759 | 1,71426 |
| 214596_at | 1131 | CHRM3 | 1,66E-06 | 7,89969E-05 | 0,540712 | -0,887068 | -1,84941 |
| 238967_at | --- | --- | 1,82E-06 | 7,89969E-05 | 0,338009 | -1,56487 | -2,9585 |
| 1555968_a_at | --- | --- | 1,85E-06 | 7,89969E-05 | 0,611726 | -0,709043 | -1,63472 |
| 202917_s_at | 6279 | S100A8 | 2,48E-06 | 9,76687E-05 | 19,6019 | 4,29292 | 19,6019 |
| 216101_at | --- | --- | 3,11E-06 | 0,000103333 | 0,361719 | -1,46706 | -2,76458 |
| 1557585_at | 51606 | ATP6V1H | 3,34E-06 | 0,000103333 | 0,571689 | -0,806699 | -1,7492 |
| 204174_at | 241 | ALOX5AP | 3,47E-06 | 0,000103333 | 3,62036 | 1,85613 | 3,62036 |
| 239790_s_at | --- | --- | 3,64E-06 | 0,000103333 | 1,7036 | 0,76859 | 1,7036 |
| 237733_at | --- | --- | 3,70E-06 | 0,000103333 | 0,422452 | -1,24314 | -2,36713 |
| 201288_at | 397 | ARHGDIB | 3,83E-06 | 0,000103333 | 2,56223 | 1,3574 | 2,56223 |
| 204860_s_at | 4671 /// 101060527 | LOC101060527 /// NAIP | 4,10E-06 | 0,00010496 | 2,66943 | 1,41653 | 2,66943 |
| 234110_at | 283075 | LOC283075 | 4,59E-06 | 0,00010617 | 0,56181 | -0,831845 | -1,77996 |
| 214725_at | 157869 | SBSPON | 4,64E-06 | 0,00010617 | 0,244056 | -2,03472 | -4,09742 |
| 203198_at | 1025 | CDK9 | 4,77E-06 | 0,00010617 | 2,29091 | 1,19592 | 2,29091 |
| 202833_s_at | 5265 | SERPINA1 | 5,29E-06 | 0,000112945 | 4,68582 | 2,2283 | 4,68582 |
| 206253_at | 1740 | DLG2 | 7,55E-06 | 0,000149966 | 0,571267 | -0,807763 | -1,7505 |
| 209949_at | 4688 | NCF2 | 7,62E-06 | 0,000149966 | 3,37223 | 1,7537 | 3,37223 |
| 215095_at | --- | --- | 8,54E-06 | 0,000162017 | 0,544332 | -0,877442 | -1,83712 |
| 1556373_a_at | 222234 | FAM185A | 9,71E-06 | 0,000177464 | 0,480226 | -1,05821 | -2,08235 |
| 217785_s_at | 10652 | YKT6 | 1,03E-05 | 0,000181767 | 2,12905 | 1,09021 | 2,12905 |
| 204899_s_at | 8819 | SAP30 | 1,25E-05 | 0,000210637 | 2,1851 | 1,1277 | 2,1851 |
| 210629_x_at | 7940 | LST1 | 1,28E-05 | 0,000210637 | 2,42899 | 1,28036 | 2,42899 |
| 1555958_at | 55118 | CRTAC1 | 1,34E-05 | 0,000210637 | 0,486848 | -1,03846 | -2,05403 |
| 217501_at | 9391 | CIAO1 | 1,37E-05 | 0,000210637 | 0,628853 | -0,669204 | -1,5902 |
| 210042_s_at | 1522 | CTSZ | 1,44E-05 | 0,000210637 | 2,20439 | 1,14038 | 2,20439 |
| 239824_s_at | 84314 | TMEM107 | 1,46E-05 | 0,000210637 | 1,51258 | 0,597015 | 1,51258 |
| 236862_at | 57120 | GOPC | 1,48E-05 | 0,000210637 | 0,367742 | -1,44324 | -2,7193 |
| 1563386_at | --- | --- | 1,58E-05 | 0,000218483 | 0,532269 | -0,909773 | -1,87875 |
| 207085_x_at | 1438 | CSF2RA | 1,70E-05 | 0,000220351 | 2,25841 | 1,17531 | 2,25841 |
| 221952_x_at | 57570 | TRMT5 | 1,70E-05 | 0,000220351 | 1,50929 | 0,593868 | 1,50929 |
| 219861_at | 55192 | DNAJC17 | 1,72E-05 | 0,000220351 | 1,64617 | 0,719115 | 1,64617 |
| 210570_x_at | 5601 | MAPK9 | 1,77E-05 | 0,000220457 | 1,59396 | 0,672613 | 1,59396 |
| 234480_at | 57796 | DKFZP761C1711 | 1,99E-05 | 0,000237917 | 0,548174 | -0,867294 | -1,82424 |
| 235575_at | --- | --- | 2,03E-05 | 0,000237917 | 0,398167 | -1,32856 | -2,51151 |
| 222551_s_at | 65265 | C8orf33 | 2,06E-05 | 0,000237917 | 1,90238 | 0,927803 | 1,90238 |
| 211494_s_at | 8671 | SLC4A4 | 2,09E-05 | 0,000237917 | 0,365084 | -1,4537 | -2,73909 |
| 226133_s_at | 83874 | TBC1D10A | 2,17E-05 | 0,00024148 | 1,80102 | 0,848816 | 1,80102 |
| 1556432_at | --- | --- | 2,25E-05 | 0,000244621 | 0,583495 | -0,777207 | -1,71381 |
| 244758_at | 114821 | SCAND3 | 2,35E-05 | 0,000244621 | 0,423307 | -1,24022 | -2,36235 |
| 228725_x_at | 3275 | PRMT2 | 2,37E-05 | 0,000244621 | 2,09684 | 1,06822 | 2,09684 |
| 1555728_a_at | 51338 | MS4A4A | 2,43E-05 | 0,000244621 | 4,2479 | 2,08675 | 4,2479 |
| 221616_s_at | 51616 | TAF9B | 2,44E-05 | 0,000244621 | 0,591444 | -0,757685 | -1,69078 |
| 214574_x_at | 7940 | LST1 | 2,59E-05 | 0,000255257 | 2,37181 | 1,24599 | 2,37181 |
| 208130_s_at | 6916 | TBXAS1 | 3,13E-05 | 0,000302744 | 2,03482 | 1,0249 | 2,03482 |
| 1554027_a_at | 8671 | SLC4A4 | 3,34E-05 | 0,000310875 | 0,437316 | -1,19325 | -2,28668 |
| 219788_at | 29992 | PILRA | 3,35E-05 | 0,000310875 | 2,13014 | 1,09095 | 2,13014 |
| 200838_at | 1508 | CTSB | 3,41E-05 | 0,000310875 | 1,65439 | 0,726301 | 1,65439 |
| 1567457_at | 5879 | RAC1 | 3,46E-05 | 0,000310875 | 0,565255 | -0,823026 | -1,76911 |
| 242251_at | 166378 | SPATA5 | 3,69E-05 | 0,00032287 | 0,634239 | -0,656901 | -1,57669 |
| 203760_s_at | 6503 | SLA | 3,76E-05 | 0,00032287 | 1,70538 | 0,770091 | 1,70538 |
| 207001_x_at | 1831 | TSC22D3 | 3,78E-05 | 0,00032287 | 2,75891 | 1,4641 | 2,75891 |
| 1553366_s_at | 51239 /// 200539 | ANKRD23 /// ANKRD39 | 3,97E-05 | 0,000333602 | 0,603452 | -0,728689 | -1,65713 |
| 203535_at | 6280 | S100A9 | 4,11E-05 | 0,000335419 | 7,61688 | 2,9292 | 7,61688 |
| 213274_s_at | 1508 | CTSB | 4,23E-05 | 0,000335419 | 2,88036 | 1,52625 | 2,88036 |
| 215633_x_at | 7940 | LST1 | 4,26E-05 | 0,000335419 | 2,05052 | 1,03599 | 2,05052 |
| 1555736_a_at | 57085 | AGTRAP | 4,32E-05 | 0,000335419 | 1,99293 | 0,994893 | 1,99293 |
| 208756_at | 8668 | EIF3I | 4,32E-05 | 0,000335419 | 1,65052 | 0,722917 | 1,65052 |
| 200622_x_at | 801 /// 805 /// 808 | CALM1 /// CALM2 /// CALM3 | 4,51E-05 | 0,000342002 | 2,04596 | 1,03278 | 2,04596 |
| 200739_s_at | 6612 | SUMO3 | 4,58E-05 | 0,000342002 | 1,60986 | 0,686935 | 1,60986 |
| 207447_s_at | 25834 | MGAT4C | 4,70E-05 | 0,000342002 | 0,293536 | -1,76839 | -3,40674 |
| 203028_s_at | 1535 | CYBA | 4,75E-05 | 0,000342002 | 2,68706 | 1,42603 | 2,68706 |
| 1559265_at | 387640 | SKIDA1 | 4,93E-05 | 0,000342002 | 0,538218 | -0,893738 | -1,85798 |
| 222686_s_at | 55313 | CPPED1 | 4,96E-05 | 0,000342002 | 1,61012 | 0,687164 | 1,61012 |
| 213571_s_at | 9470 | EIF4E2 | 4,99E-05 | 0,000342002 | 2,81651 | 1,49391 | 2,81651 |
| 213733_at | 4542 | MYO1F | 5,05E-05 | 0,000342002 | 1,98839 | 0,991604 | 1,98839 |
| 225626_at | 55824 | PAG1 | 5,15E-05 | 0,000342002 | 1,81531 | 0,860217 | 1,81531 |
| 201954_at | 10095 | ARPC1B | 5,16E-05 | 0,000342002 | 1,91929 | 0,940574 | 1,91929 |
| 221059_s_at | 23406 | COTL1 | 5,20E-05 | 0,000342002 | 2,65207 | 1,40712 | 2,65207 |
| 200078_s_at | 533 | ATP6V0B | 5,26E-05 | 0,000342002 | 1,52057 | 0,604611 | 1,52057 |
| 1556919_at | 3953 | LEPR | 5,31E-05 | 0,000342002 | 0,649281 | -0,623084 | -1,54016 |
| 220037_s_at | 10894 | LYVE1 | 5,38E-05 | 0,000342002 | 4,22907 | 2,08034 | 4,22907 |
| 201526_at | 381 | ARF5 | 5,47E-05 | 0,000342002 | 1,81679 | 0,861391 | 1,81679 |
| 219059_s_at | 10894 | LYVE1 | 5,48E-05 | 0,000342002 | 2,33427 | 1,22297 | 2,33427 |
| 212766_s_at | 81875 | ISG20L2 | 5,74E-05 | 0,000354097 | 1,79711 | 0,845676 | 1,79711 |
| 1559033_at | 255167 | LOC255167 | 5,94E-05 | 0,000354564 | 0,337047 | -1,56898 | -2,96694 |
| 201004_at | 6748 | SSR4 | 5,99E-05 | 0,000354564 | 1,51942 | 0,603522 | 1,51942 |
| 239709_at | --- | --- | 6,05E-05 | 0,000354564 | 0,633374 | -0,65887 | -1,57885 |
| 1554455_at | 55180 | LINS | 6,06E-05 | 0,000354564 | 0,384494 | -1,37897 | -2,60082 |
| 209132_s_at | 54939 | COMMD4 | 6,17E-05 | 0,000354564 | 1,65001 | 0,722472 | 1,65001 |
| 1553471_at | 146861 | SLC35G3 | 6,29E-05 | 0,000354564 | 0,640059 | -0,643724 | -1,56236 |
| 214181_x_at | 7940 | LST1 | 6,30E-05 | 0,000354564 | 2,82791 | 1,49973 | 2,82791 |
| 202407_s_at | 26121 | PRPF31 | 6,32E-05 | 0,000354564 | 1,82913 | 0,871161 | 1,82913 |
| 204829_s_at | 2350 | FOLR2 | 6,47E-05 | 0,000354564 | 2,30553 | 1,2051 | 2,30553 |
| 225604_s_at | 152007 | GLIPR2 | 6,55E-05 | 0,000354564 | 4,47687 | 2,16249 | 4,47687 |
| 236808_at | 26127 | FGFR1OP2 | 6,57E-05 | 0,000354564 | 0,417705 | -1,25944 | -2,39404 |
| 204563_at | 6402 | SELL | 6,58E-05 | 0,000354564 | 3,65887 | 1,8714 | 3,65887 |
| 1554899_s_at | 2207 | FCER1G | 6,69E-05 | 0,000354586 | 6,10428 | 2,60982 | 6,10428 |
| 224107_at | --- | --- | 6,72E-05 | 0,000354586 | 0,55243 | -0,856136 | -1,81018 |
| 200744_s_at | 2782 | GNB1 | 6,81E-05 | 0,000354724 | 2,88788 | 1,53001 | 2,88788 |
| 200736_s_at | 2876 | GPX1 | 6,86E-05 | 0,000354724 | 2,54495 | 1,34764 | 2,54495 |
| 210936_at | 9948 | WDR1 | 6,99E-05 | 0,000356352 | 0,666458 | -0,585415 | -1,50047 |
| 217871_s_at | 4282 | MIF | 7,11E-05 | 0,000356352 | 3,15466 | 1,65748 | 3,15466 |
| 208447_s_at | 5631 | PRPS1 | 7,13E-05 | 0,000356352 | 1,98396 | 0,98838 | 1,98396 |
| 1564776_at | --- | --- | 7,17E-05 | 0,000356352 | 0,605463 | -0,72389 | -1,65163 |
| 211582_x_at | 7940 | LST1 | 7,26E-05 | 0,000357369 | 2,56597 | 1,3595 | 2,56597 |
| 226320_at | 10189 | ALYREF | 7,72E-05 | 0,000368784 | 1,84707 | 0,88524 | 1,84707 |
| 230807_at | 115948 | CCDC151 | 7,79E-05 | 0,000368784 | 1,69545 | 0,761669 | 1,69545 |
| 208335_s_at | 2532 | DARC | 7,80E-05 | 0,000368784 | 3,19067 | 1,67386 | 3,19067 |
| 238303_at | --- | --- | 7,83E-05 | 0,000368784 | 0,526005 | -0,926852 | -1,90112 |
| 230026_at | 84545 | MRPL43 | 7,85E-05 | 0,000368784 | 2,77333 | 1,47162 | 2,77333 |
| 1556935_at | --- | --- | 8,02E-05 | 0,000373326 | 0,385593 | -1,37485 | -2,59341 |
| 211581_x_at | 7940 | LST1 | 8,14E-05 | 0,000374117 | 2,3585 | 1,23787 | 2,3585 |
| 227155_at | 8543 | LMO4 | 8,20E-05 | 0,000374117 | 0,487133 | -1,03761 | -2,05283 |
| 202736_s_at | 25804 | LSM4 | 8,26E-05 | 0,000374117 | 1,69632 | 0,76241 | 1,69632 |
| 204232_at | 2207 | FCER1G | 8,33E-05 | 0,000374141 | 3,21318 | 1,684 | 3,21318 |
| 210667_s_at | 8209 | C21orf33 | 8,40E-05 | 0,000374141 | 2,23993 | 1,16346 | 2,23993 |
| 233069_at | 55370 | PPP4R1L | 8,55E-05 | 0,000376092 | 0,495535 | -1,01294 | -2,01802 |
| 1560258_a_at | --- | CTB-92J24.2 /// OTTHUMG00000183390 | 8,59E-05 | 0,000376092 | 0,556546 | -0,845426 | -1,7968 |
| 1553710_at | 152756 | FAM218A | 9,12E-05 | 0,000384856 | 0,619947 | -0,689782 | -1,61304 |
| 1563283_at | --- | --- | 9,19E-05 | 0,000384856 | 0,602766 | -0,730331 | -1,65902 |
| 1557505_a_at | --- | --- | 9,24E-05 | 0,000384856 | 0,53205 | -0,910367 | -1,87952 |
| 202957_at | 3059 | HCLS1 | 9,34E-05 | 0,000384856 | 2,46164 | 1,29962 | 2,46164 |
| 201642_at | 3460 | IFNGR2 | 9,35E-05 | 0,000384856 | 1,59166 | 0,670534 | 1,59166 |
| 202887_s_at | 54541 | DDIT4 | 9,35E-05 | 0,000384856 | 2,51872 | 1,33269 | 2,51872 |
| 226017_at | 112616 | CMTM7 | 9,37E-05 | 0,000384856 | 1,60977 | 0,686851 | 1,60977 |
| 214454_at | 9509 | ADAMTS2 | 9,53E-05 | 0,000384856 | 2,13149 | 1,09186 | 2,13149 |
| 203415_at | 10016 | PDCD6 | 9,60E-05 | 0,000384856 | 2,16167 | 1,11215 | 2,16167 |
| 202858_at | 7307 | U2AF1 | 9,61E-05 | 0,000384856 | 1,91618 | 0,938234 | 1,91618 |
| 223234_at | 10459 | MAD2L2 | 9,66E-05 | 0,000384856 | 2,19087 | 1,1315 | 2,19087 |
| 222583_s_at | 10762 | NUP50 | 9,70E-05 | 0,000384856 | 2,1051 | 1,07389 | 2,1051 |
| 239788_at | --- | --- | 9,91E-05 | 0,00039014 | 0,534121 | -0,904761 | -1,87223 |
| 232652_x_at | 51282 | SCAND1 | 0,00010188 | 0,00039557 | 1,88855 | 0,917277 | 1,88855 |
| 213530_at | 22930 | RAB3GAP1 | 0,00010253 | 0,00039557 | 0,567582 | -0,8171 | -1,76186 |
| 200968_s_at | 5479 | PPIB | 0,00010288 | 0,00039557 | 5,28771 | 2,40264 | 5,28771 |
| 205862_at | 9687 | GREB1 | 0,00010353 | 0,00039557 | 0,543537 | -0,87955 | -1,8398 |
| 218559_s_at | 9935 | MAFB | 0,00011055 | 0,000419279 | 2,25333 | 1,17206 | 2,25333 |
| 207565_s_at | 3140 | MR1 | 0,00011183 | 0,000421007 | 1,74873 | 0,806305 | 1,74873 |
| 1569436_at | 400128 | LOC400128 | 0,0001147 | 0,000428645 | 0,658475 | -0,602801 | -1,51866 |
| 1557014_a_at | 158228 | FAM201A | 0,00011703 | 0,000434191 | 0,3194 | -1,64656 | -3,13087 |
| 223328_at | 83787 | ARMC10 | 0,00011881 | 0,000437642 | 1,65407 | 0,726021 | 1,65407 |
| 244832_at | --- | OTTHUMG00000161357 /// RP11-752L20.3 | 0,00012152 | 0,000444416 | 0,591773 | -0,756884 | -1,68984 |
| 233007_at | --- | --- | 0,0001264 | 0,000458966 | 0,511111 | -0,968293 | -1,95652 |
| 225208_s_at | 83640 | FAM103A1 | 0,00012952 | 0,000466984 | 1,85297 | 0,889836 | 1,85297 |
| 214442_s_at | 9063 | PIAS2 | 0,00013073 | 0,000468075 | 1,52316 | 0,607063 | 1,52316 |
| 219191_s_at | 51411 | BIN2 | 0,00013208 | 0,000469625 | 2,02973 | 1,02129 | 2,02973 |
| 205048_s_at | 5723 | PSPH | 0,00013348 | 0,0004707 | 12,4952 | 3,6433 | 12,4952 |
| 205965_at | 10538 | BATF | 0,00013422 | 0,0004707 | 1,6359 | 0,710089 | 1,6359 |
| 223312_at | 84279 | PRADC1 | 0,00013656 | 0,000475452 | 2,17526 | 1,12119 | 2,17526 |
| 207008_at | 3579 | CXCR2 | 0,00013874 | 0,000475452 | 6,81594 | 2,76891 | 6,81594 |
| 1559102_at | --- | OTTHUMG00000176315 /// RP11-73K9.2 | 0,00014033 | 0,000475452 | 0,614594 | -0,702294 | -1,62709 |
| 235261_at | 146862 | UNC45B | 0,00014036 | 0,000475452 | 0,567154 | -0,818189 | -1,76319 |
| 235163_at | 126308 | MOB3A | 0,00014083 | 0,000475452 | 1,62001 | 0,696003 | 1,62001 |
| 241662_x_at | --- | --- | 0,00014171 | 0,000475452 | 0,592417 | -0,755315 | -1,688 |
| 217308_at | 26184 | OR1F2P | 0,00014241 | 0,000475452 | 0,475734 | -1,07177 | -2,10202 |
| 221700_s_at | 7311 | UBA52 | 0,00014502 | 0,000475452 | 1,60087 | 0,678852 | 1,60087 |
| 208148_at | 4622 | MYH4 | 0,00014512 | 0,000475452 | 0,598807 | -0,739836 | -1,66999 |
| 235652_at | --- | --- | 0,00014528 | 0,000475452 | 0,41342 | -1,27432 | -2,41885 |
| 210321_at | 2999 | GZMH | 0,00014579 | 0,000475452 | 2,15121 | 1,10515 | 2,15121 |
| 206120_at | 945 | CD33 | 0,00014775 | 0,000478775 | 1,68649 | 0,754027 | 1,68649 |
| 210945_at | 1288 | COL4A6 | 0,00014922 | 0,00048052 | 0,530228 | -0,915315 | -1,88598 |
| 232549_at | 54033 | RBM11 | 0,00015195 | 0,000482232 | 0,477158 | -1,06746 | -2,09574 |
| 211429_s_at | 5265 | SERPINA1 | 0,00015286 | 0,000482232 | 3,88183 | 1,95674 | 3,88183 |
| 237480_at | --- | --- | 0,00015312 | 0,000482232 | 0,536268 | -0,898974 | -1,86474 |
| 202397_at | 10204 /// 128322 | LOC128322 /// NUTF2 | 0,00015386 | 0,000482232 | 4,15771 | 2,05579 | 4,15771 |
| 209473_at | 953 | ENTPD1 | 0,00015447 | 0,000482232 | 2,17738 | 1,12259 | 2,17738 |
| 213061_s_at | 123803 | NTAN1 | 0,00015649 | 0,000483665 | 2,02632 | 1,01886 | 2,02632 |
| 240145_at | --- | --- | 0,00015691 | 0,000483665 | 0,407384 | -1,29554 | -2,45469 |
| 212041_at | 9114 | ATP6V0D1 | 0,00015781 | 0,000483665 | 2,12429 | 1,08698 | 2,12429 |
| 1559949_at | --- | --- | 0,00015967 | 0,000483665 | 0,308634 | -1,69603 | -3,24009 |
| 210699_at | --- | --- | 0,0001603 | 0,000483665 | 0,626833 | -0,673846 | -1,59532 |
| 225282_at | 64744 | SMAP2 | 0,00016059 | 0,000483665 | 2,05836 | 1,0415 | 2,05836 |
| 232543_x_at | 64333 | ARHGAP9 | 0,00016242 | 0,000486319 | 2,51949 | 1,33313 | 2,51949 |
| 224387_at | 28991 | COMMD5 | 0,00016528 | 0,000492008 | 1,50434 | 0,58913 | 1,50434 |
| 206768_at | 6123 | RPL3L | 0,00016653 | 0,00049284 | 1,982 | 0,986955 | 1,982 |
| 206941_x_at | 9723 | SEMA3E | 0,00016838 | 0,000492889 | 0,477863 | -1,06533 | -2,09265 |
| 203561_at | 2212 | FCGR2A | 0,00016847 | 0,000492889 | 2,86296 | 1,51751 | 2,86296 |
| 203186_s_at | 6275 | S100A4 | 0,00016961 | 0,000493405 | 4,22739 | 2,07977 | 4,22739 |
| 228532_at | 128346 | C1orf162 | 0,00017158 | 0,00049633 | 2,24994 | 1,16988 | 2,24994 |
| 212165_at | 92703 /// 653659 | TMEM183A /// TMEM183B | 0,00017433 | 0,000501259 | 1,87021 | 0,903201 | 1,87021 |
| 228351_at | 55127 | HEATR1 | 0,00017614 | 0,000501259 | 0,657333 | -0,605305 | -1,5213 |
| 230790_x_at | --- | --- | 0,00017622 | 0,000501259 | 0,471374 | -1,08506 | -2,12146 |
| 1555889_a_at | 10491 /// 100653071 | CRTAP /// LOC100653071 | 0,000178 | 0,000501337 | 1,52644 | 0,610175 | 1,52644 |
| 219371_s_at | 10365 | KLF2 | 0,00017904 | 0,000501337 | 3,60144 | 1,84858 | 3,60144 |
| 204109_s_at | 4800 | NFYA | 0,00017996 | 0,000501337 | 1,80021 | 0,848165 | 1,80021 |
| 225401_at | 112770 | C1orf85 | 0,00018017 | 0,000501337 | 1,88991 | 0,918319 | 1,88991 |
| 213187_x_at | 2512 | FTL | 0,00018198 | 0,000503634 | 2,15822 | 1,10984 | 2,15822 |
| 225878_at | 23095 | KIF1B | 0,00018323 | 0,00050401 | 0,662839 | -0,593269 | -1,50866 |
| 211101_x_at | 11027 | LILRA2 | 0,00018472 | 0,00050401 | 1,79929 | 0,847428 | 1,79929 |
| 1557065_at | 56252 | YLPM1 | 0,00018507 | 0,00050401 | 0,570755 | -0,809056 | -1,75206 |
| 203530_s_at | 6810 | STX4 | 0,00018843 | 0,000509295 | 2,12247 | 1,08574 | 2,12247 |
| 222364_at | 23446 | SLC44A1 | 0,00018984 | 0,000509295 | 0,451586 | -1,14693 | -2,21442 |
| 244047_at | --- | --- | 0,00018999 | 0,000509295 | 0,421474 | -1,24649 | -2,37263 |
| 217309_s_at | 10311 | DSCR3 | 0,00019291 | 0,000514424 | 1,98427 | 0,988606 | 1,98427 |
| 219666_at | 64231 | MS4A6A | 0,00019764 | 0,00052432 | 3,43113 | 1,77869 | 3,43113 |
| 219710_at | 79628 | SH3TC2 | 0,00019967 | 0,000526956 | 0,653761 | -0,613164 | -1,52961 |
| 202296_s_at | 11079 | RER1 | 0,00020479 | 0,000537266 | 1,59898 | 0,677149 | 1,59898 |
| 209225_x_at | 3842 | TNPO1 | 0,00020567 | 0,000537266 | 0,612091 | -0,708183 | -1,63374 |
| 208819_at | 4218 | RAB8A | 0,00021375 | 0,000553886 | 1,90576 | 0,930367 | 1,90576 |
| 209449_at | 57819 | LSM2 | 0,0002142 | 0,000553886 | 1,90293 | 0,928221 | 1,90293 |
| 233059_at | 3760 | KCNJ3 | 0,00021628 | 0,000554623 | 0,385319 | -1,37587 | -2,59525 |
| 204479_at | 26578 | OSTF1 | 0,00021713 | 0,000554623 | 2,41087 | 1,26955 | 2,41087 |
| 201541_s_at | 10467 | ZNHIT1 | 0,00021773 | 0,000554623 | 2,29352 | 1,19756 | 2,29352 |
| 235458_at | 84868 | HAVCR2 | 0,00021938 | 0,00055606 | 1,972 | 0,979662 | 1,972 |
| 225328_at | 114907 | FBXO32 | 0,00022306 | 0,000562582 | 0,568601 | -0,814512 | -1,7587 |
| 201143_s_at | 1965 | EIF2S1 | 0,00023223 | 0,000582839 | 2,87782 | 1,52498 | 2,87782 |
| 214250_at | 4926 | NUMA1 | 0,00023522 | 0,000585022 | 0,611226 | -0,710222 | -1,63606 |
| 200621_at | 1465 | CSRP1 | 0,00023538 | 0,000585022 | 2,07487 | 1,05302 | 2,07487 |
| 233425_at | --- | --- | 0,00023697 | 0,000586124 | 0,571423 | -0,80737 | -1,75002 |
| 219799_s_at | 10170 | DHRS9 | 0,00023824 | 0,000586442 | 3,42712 | 1,777 | 3,42712 |
| 231393_x_at | 23099 | ZBTB43 | 0,00024002 | 0,000587984 | 0,644078 | -0,634692 | -1,55261 |
| 1567183_s_at | --- | AC069277.2 /// OTTHUMG00000154923 | 0,00024326 | 0,000593079 | 0,649318 | -0,623003 | -1,54008 |
| 239400_at | 729220 | FLJ45513 | 0,00025327 | 0,000614558 | 0,629732 | -0,667191 | -1,58798 |
| 228047_at | 26775 | SNORA72 | 0,0002564 | 0,000617898 | 0,527736 | -0,922111 | -1,89489 |
| 220243_at | 29068 | ZBTB44 | 0,00025791 | 0,000617898 | 0,613499 | -0,704867 | -1,62999 |
| 204861_s_at | 4671 /// 101060527 | LOC101060527 /// NAIP | 0,00025874 | 0,000617898 | 2,06133 | 1,04357 | 2,06133 |
| 218077_s_at | 51304 | ZDHHC3 | 0,00025947 | 0,000617898 | 1,68462 | 0,752427 | 1,68462 |
| 205349_at | 2769 | GNA15 | 0,00026268 | 0,000621117 | 1,64082 | 0,714415 | 1,64082 |
| 228212_at | 145501 | ISM2 | 0,00026445 | 0,000621117 | 0,630514 | -0,665399 | -1,58601 |
| 230689_at | --- | --- | 0,00026446 | 0,000621117 | 0,611802 | -0,708864 | -1,63452 |
| 223306_at | 84650 | EBPL | 0,00026605 | 0,000621986 | 1,56436 | 0,645572 | 1,56436 |
| 207128_s_at | 7766 | ZNF223 | 0,0002718 | 0,000627701 | 0,642998 | -0,637113 | -1,55521 |
| 210573_s_at | 10623 /// 101060460 | LOC101060460 /// POLR3C | 0,00027207 | 0,000627701 | 1,99805 | 0,99859 | 1,99805 |
| 209965_s_at | 5892 | RAD51D | 0,00027405 | 0,000627701 | 1,56085 | 0,642336 | 1,56085 |
| 204446_s_at | 240 | ALOX5 | 0,00027461 | 0,000627701 | 2,11808 | 1,08276 | 2,11808 |
| 215316_at | --- | --- | 0,00027462 | 0,000627701 | 0,602775 | -0,730308 | -1,65899 |
| 204787_at | 11326 | VSIG4 | 0,00027676 | 0,000629778 | 2,41727 | 1,27338 | 2,41727 |
| 210108_at | 776 | CACNA1D | 0,00028285 | 0,000640786 | 0,415473 | -1,26717 | -2,4069 |
| 226051_at | 140606 | SELM | 0,00028671 | 0,000646113 | 2,01281 | 1,00921 | 2,01281 |
| 202159_at | 2193 | FARSA | 0,00028808 | 0,000646113 | 1,66619 | 0,736553 | 1,66619 |
| 217478_s_at | 3108 | HLA-DMA | 0,00029135 | 0,000646113 | 2,38154 | 1,25189 | 2,38154 |
| 217930_s_at | 54472 | TOLLIP | 0,00029213 | 0,000646113 | 1,73196 | 0,792401 | 1,73196 |
| 212829_at | 5305 | PIP4K2A | 0,00029244 | 0,000646113 | 1,90241 | 0,927828 | 1,90241 |
| 221479_s_at | 665 | BNIP3L | 0,00029337 | 0,000646113 | 1,55004 | 0,632308 | 1,55004 |
| 220986_s_at | 81789 | TIGD6 | 0,00029403 | 0,000646113 | 0,653065 | -0,614702 | -1,53124 |
| 225065_x_at | 26800 /// 125144 /// 692087 /// 692106 | C17orf76-AS1 /// SNORD49A /// SNORD49B /// SNORD65 | 0,00030353 | 0,000661898 | 3,21325 | 1,68403 | 3,21325 |
| 1564482_at | 539 | ATP5O | 0,000304 | 0,000661898 | 0,601135 | -0,734239 | -1,66352 |
| 217770_at | 51604 | PIGT | 0,00030577 | 0,000661898 | 2,40442 | 1,26569 | 2,40442 |
| 237491_at | --- | --- | 0,00030721 | 0,000661898 | 0,301158 | -1,73141 | -3,32052 |
| 204122_at | 7305 | TYROBP | 0,00030793 | 0,000661898 | 5,25664 | 2,39414 | 5,25664 |
| 209201_x_at | 7852 | CXCR4 | 0,0003112 | 0,000661898 | 2,7657 | 1,46764 | 2,7657 |
| 230789_at | 140883 | ZNF280B | 0,00031121 | 0,000661898 | 0,624326 | -0,679629 | -1,60173 |
| 208659_at | 1192 | CLIC1 | 0,00031241 | 0,000661898 | 2,52758 | 1,33775 | 2,52758 |
| 212625_at | 8677 | STX10 | 0,00031285 | 0,000661898 | 1,91042 | 0,933888 | 1,91042 |
| 229464_at | 50804 | MYEF2 | 0,0003179 | 0,000667862 | 0,621007 | -0,687318 | -1,61029 |
| 1554466_a_at | 84326 | C16orf13 | 0,00031906 | 0,000667862 | 1,79972 | 0,847775 | 1,79972 |
| 222670_s_at | 9935 | MAFB | 0,00032032 | 0,000667862 | 2,01766 | 1,01268 | 2,01766 |
| 218913_s_at | 51291 | GMIP | 0,00032089 | 0,000667862 | 1,8678 | 0,90134 | 1,8678 |
| 204319_s_at | 6001 | RGS10 | 0,00032659 | 0,000674508 | 2,49988 | 1,32186 | 2,49988 |
| 1565887_at | --- | --- | 0,00032769 | 0,000674508 | 0,266643 | -1,90702 | -3,75033 |
| 209693_at | 23245 | ASTN2 | 0,00032803 | 0,000674508 | 0,485567 | -1,04226 | -2,05945 |
| 201863_at | 26017 | FAM32A | 0,00033362 | 0,000683254 | 1,94868 | 0,962495 | 1,94868 |
| 236000_s_at | --- | --- | 0,00033707 | 0,000687575 | 0,524576 | -0,930776 | -1,9063 |
| 241464_s_at | --- | hsa-let-7a-3 /// hsa-let-7b /// hsa-mir-4763 /// OTTHUMG00000150446 /// RP4-695O20__B.10 | 0,00034326 | 0,000695666 | 0,650791 | -0,619733 | -1,53659 |
| 224583_at | 23406 | COTL1 | 0,00034376 | 0,000695666 | 3,28157 | 1,71439 | 3,28157 |
| 204043_at | 6948 | TCN2 | 0,00034678 | 0,000699015 | 1,95 | 0,963476 | 1,95 |
| 224097_s_at | 50848 | F11R | 0,00034997 | 0,000702677 | 1,60006 | 0,678123 | 1,60006 |
| 244107_at | --- | --- | 0,00035425 | 0,000702947 | 0,463099 | -1,11061 | -2,15936 |
| 219774_at | 54520 | CCDC93 | 0,000355 | 0,000702947 | 1,68236 | 0,750484 | 1,68236 |
| 233351_at | --- | --- | 0,00035508 | 0,000702947 | 0,56632 | -0,82031 | -1,76579 |
| 244121_at | 130507 | UBR3 | 0,00035668 | 0,000702947 | 0,568109 | -0,815761 | -1,76023 |
| 208438_s_at | 2268 | FGR | 0,00036015 | 0,000702947 | 1,9824 | 0,987249 | 1,9824 |
| 201721_s_at | 7805 | LAPTM5 | 0,00036075 | 0,000702947 | 2,80971 | 1,49042 | 2,80971 |
| 227186_s_at | 64975 | MRPL41 | 0,00036104 | 0,000702947 | 2,75796 | 1,4636 | 2,75796 |
| 1559103_s_at | --- | OTTHUMG00000176315 /// RP11-73K9.2 | 0,00036108 | 0,000702947 | 0,564234 | -0,825635 | -1,77231 |
| 218206_x_at | 51282 | SCAND1 | 0,00036353 | 0,000705036 | 1,63214 | 0,706767 | 1,63214 |
| 218237_s_at | 81539 | SLC38A1 | 0,00036606 | 0,00070528 | 0,465461 | -1,10327 | -2,14841 |
| 223369_at | 28989 | NTMT1 | 0,00036642 | 0,00070528 | 1,84664 | 0,884905 | 1,84664 |
| 204502_at | 25939 | SAMHD1 | 0,00037549 | 0,000709689 | 1,735 | 0,794933 | 1,735 |
| 221253_s_at | 81567 /// 100526836 | BLOC1S5-TXNDC5 /// TXNDC5 | 0,00037572 | 0,000709689 | 2,33211 | 1,22164 | 2,33211 |
| 233563_s_at | 54973 | CPSF3L | 0,00037641 | 0,000709689 | 1,56102 | 0,642488 | 1,56102 |
| 223883_s_at | 56164 | STK31 | 0,00037709 | 0,000709689 | 0,512185 | -0,965263 | -1,95242 |
| 237410_x_at | --- | --- | 0,00037723 | 0,000709689 | 0,653294 | -0,614195 | -1,5307 |
| 217865_at | 55819 | RNF130 | 0,00038091 | 0,000709689 | 3,37327 | 1,75415 | 3,37327 |
| 205237_at | 2219 | FCN1 | 0,00038291 | 0,000709689 | 2,59904 | 1,37798 | 2,59904 |
| 240965_at | --- | --- | 0,00038532 | 0,000709689 | 0,601297 | -0,733851 | -1,66307 |
| 244050_at | 401494 | PTPLAD2 | 0,00038535 | 0,000709689 | 2,05199 | 1,03702 | 2,05199 |
| 211386_at | 84786 | MGC12488 | 0,00038559 | 0,000709689 | 0,578006 | -0,790843 | -1,73009 |
| 224598_at | 11282 | MGAT4B | 0,00039068 | 0,000709689 | 2,3172 | 1,21238 | 2,3172 |
| 202802_at | 1725 | DHPS | 0,00039121 | 0,000709689 | 1,84281 | 0,881909 | 1,84281 |
| 232063_x_at | 10056 | FARSB | 0,00039134 | 0,000709689 | 0,470806 | -1,08679 | -2,12402 |
| 1555910_at | 79810 | PTCD2 | 0,0003926 | 0,000709689 | 0,564735 | -0,824354 | -1,77074 |
| 203430_at | 23593 | HEBP2 | 0,000393 | 0,000709689 | 2,15107 | 1,10505 | 2,15107 |
| 222218_s_at | 29992 | PILRA | 0,00039511 | 0,000709689 | 2,18825 | 1,12978 | 2,18825 |
| 219215_s_at | 55630 | SLC39A4 | 0,00039555 | 0,000709689 | 1,68994 | 0,756968 | 1,68994 |
| 206050_s_at | 6050 | RNH1 | 0,00039608 | 0,000709689 | 1,96292 | 0,973005 | 1,96292 |
| 219506_at | 79630 | C1orf54 | 0,00039611 | 0,000709689 | 2,8302 | 1,5009 | 2,8302 |
| 232489_at | 54482 | TRMT13 | 0,000397 | 0,000709689 | 0,337731 | -1,56606 | -2,96094 |
| 233090_at | --- | --- | 0,00039781 | 0,000709689 | 0,654244 | -0,6121 | -1,52848 |
| 221908_at | 84900 | RNFT2 | 0,00040123 | 0,000713289 | 1,61627 | 0,692667 | 1,61627 |
| 200971_s_at | 27230 | SERP1 | 0,00040623 | 0,000717928 | 1,87413 | 0,906225 | 1,87413 |
| 205632_s_at | 8395 | PIP5K1B | 0,00040664 | 0,000717928 | 0,497426 | -1,00745 | -2,01035 |
| 218102_at | 51071 | DERA | 0,00040901 | 0,000718318 | 1,69805 | 0,763879 | 1,69805 |
| 223468_s_at | 56963 | RGMA | 0,00040967 | 0,000718318 | 1,6473 | 0,720106 | 1,6473 |
| 230315_at | --- | --- | 0,00041345 | 0,00071957 | 0,546287 | -0,87227 | -1,83054 |
| 200951_s_at | 894 | CCND2 | 0,00041413 | 0,00071957 | 1,6266 | 0,701856 | 1,6266 |
| 211852_s_at | 8455 | ATRN | 0,00041646 | 0,00071957 | 1,86071 | 0,89585 | 1,86071 |
| 240806_at | 6138 | RPL15 | 0,000417 | 0,00071957 | 1,60487 | 0,682454 | 1,60487 |
| 1553527_at | 338321 | NLRP9 | 0,00041855 | 0,00071957 | 0,652553 | -0,615832 | -1,53244 |
| 233243_at | --- | --- | 0,00041881 | 0,00071957 | 0,568924 | -0,813691 | -1,7577 |
| 226717_at | 220074 | LRTOMT | 0,00042271 | 0,000723833 | 1,80102 | 0,848811 | 1,80102 |
| 1558494_at | --- | --- | 0,00042614 | 0,000727286 | 0,652531 | -0,615882 | -1,53249 |
| 1555812_a_at | 397 | ARHGDIB | 0,00042946 | 0,000730502 | 2,77716 | 1,47361 | 2,77716 |
| 239761_at | 2650 | GCNT1 | 0,00043347 | 0,000734881 | 1,52202 | 0,605989 | 1,52202 |
| 203022_at | 10535 | RNASEH2A | 0,00043806 | 0,000740224 | 1,65227 | 0,724452 | 1,65227 |
| 200024_at | 6193 | RPS5 | 0,00044372 | 0,000745496 | 2,36964 | 1,24467 | 2,36964 |
| 1566507_a_at | 26268 | FBXO9 | 0,00044409 | 0,000745496 | 0,658067 | -0,603693 | -1,5196 |
| 205119_s_at | 2357 | FPR1 | 0,00044785 | 0,00074934 | 2,63705 | 1,39892 | 2,63705 |
| 204106_at | 7016 | TESK1 | 0,0004517 | 0,000753322 | 1,9099 | 0,933498 | 1,9099 |
| 217232_x_at | 3043 | HBB | 0,00045638 | 0,000758661 | 5,4206 | 2,43845 | 5,4206 |
| 208018_s_at | 3055 | HCK | 0,00046009 | 0,000759237 | 2,36082 | 1,23929 | 2,36082 |
| 1558251_a_at | 84914 | ZNF587 | 0,00046057 | 0,000759237 | 0,518296 | -0,948153 | -1,9294 |
| 216570_x_at | --- | OTTHUMG00000009792 /// RP4-595K12.1 | 0,00046118 | 0,000759237 | 1,67442 | 0,743666 | 1,67442 |
| 240512_x_at | 386618 | KCTD4 | 0,00046606 | 0,000762707 | 0,540884 | -0,886608 | -1,84882 |
| 224535_s_at | 78988 | MRP63 | 0,0004663 | 0,000762707 | 1,69872 | 0,764448 | 1,69872 |
| 240652_at | --- | --- | 0,00046775 | 0,000762707 | 0,538613 | -0,892678 | -1,85662 |
| 205070_at | 54556 | ING3 | 0,00047312 | 0,000769009 | 0,623373 | -0,681832 | -1,60418 |
| 242208_at | 100129482 | ZNF37BP | 0,00047492 | 0,000769487 | 0,589821 | -0,761651 | -1,69543 |
| 217733_s_at | 9168 | TMSB10 | 0,00047794 | 0,000771943 | 2,20082 | 1,13804 | 2,20082 |
| 226384_at | 84513 | PPAPDC1B | 0,00048223 | 0,000776427 | 2,30976 | 1,20774 | 2,30976 |
| 214617_at | 5551 | PRF1 | 0,00048508 | 0,000778556 | 1,79729 | 0,84582 | 1,79729 |
| 244112_x_at | --- | --- | 0,00048666 | 0,000778654 | 0,465075 | -1,10446 | -2,15019 |
| 226219_at | 257106 | ARHGAP30 | 0,00048899 | 0,000779067 | 2,11833 | 1,08293 | 2,11833 |
| 227850_x_at | 148170 | CDC42EP5 | 0,00049016 | 0,000779067 | 1,8923 | 0,920143 | 1,8923 |
| 232405_at | --- | --- | 0,0004915 | 0,000779067 | 0,607899 | -0,718096 | -1,64501 |
| 201858_s_at | 5552 | SRGN | 0,00049369 | 0,000779067 | 4,34701 | 2,12002 | 4,34701 |
| 229650_s_at | 79086 | SMIM7 | 0,00049811 | 0,000779067 | 1,8195 | 0,863541 | 1,8195 |
| 219263_at | 79589 | RNF128 | 0,00049871 | 0,000779067 | 0,337824 | -1,56566 | -2,96013 |
| 235961_at | 23432 | GPR161 | 0,00049893 | 0,000779067 | 1,652 | 0,724212 | 1,652 |
| 218387_s_at | 25796 | PGLS | 0,00049909 | 0,000779067 | 1,98674 | 0,990404 | 1,98674 |
| 1553297_a_at | 1441 | CSF3R | 0,00050099 | 0,000779652 | 2,36523 | 1,24198 | 2,36523 |
| 242572_at | --- | --- | 0,00050494 | 0,000783417 | 0,401331 | -1,31713 | -2,49171 |
| 216456_at | --- | --- | 0,00050691 | 0,000784101 | 0,598117 | -0,741501 | -1,67191 |
| 223344_s_at | 58475 | MS4A7 | 0,00051152 | 0,000787369 | 2,51405 | 1,33001 | 2,51405 |
| 220027_s_at | 54922 | RASIP1 | 0,00051401 | 0,000787369 | 2,19144 | 1,13188 | 2,19144 |
| 37012_at | 832 | CAPZB | 0,0005144 | 0,000787369 | 2,28315 | 1,19102 | 2,28315 |
| 203835_at | 2615 | LRRC32 | 0,00051555 | 0,000787369 | 2,37141 | 1,24574 | 2,37141 |
| 212003_at | 26099 | SZRD1 | 0,00051806 | 0,000787369 | 1,5806 | 0,660471 | 1,5806 |
| 220755_s_at | 50854 | C6orf48 | 0,00051825 | 0,000787369 | 2,22026 | 1,15073 | 2,22026 |
| 202953_at | 713 | C1QB | 0,00052099 | 0,000789193 | 3,61064 | 1,85225 | 3,61064 |
| 219100_at | 79991 | OBFC1 | 0,00052406 | 0,000791493 | 1,72134 | 0,783531 | 1,72134 |
| 204007_at | 2215 | FCGR3B | 0,00052742 | 0,000794226 | 4,24315 | 2,08513 | 4,24315 |
| 204214_s_at | 10981 | RAB32 | 0,00052932 | 0,000794761 | 2,85111 | 1,51152 | 2,85111 |
| 209116_x_at | 3043 | HBB | 0,00053095 | 0,000794871 | 5,20922 | 2,38107 | 5,20922 |
| 237664_at | --- | --- | 0,00053339 | 0,000794959 | 0,587443 | -0,767479 | -1,70229 |
| 243963_at | --- | --- | 0,00053411 | 0,000794959 | 0,407549 | -1,29496 | -2,45369 |
| 236150_at | 123688 | HYKK | 0,00053931 | 0,000800361 | 1,73317 | 0,79341 | 1,73317 |
| 1554285_at | 84868 | HAVCR2 | 0,0005445 | 0,000804392 | 1,97985 | 0,985389 | 1,97985 |
| 200652_at | 6746 | SSR2 | 0,0005488 | 0,000804392 | 2,0379 | 1,02708 | 2,0379 |
| 1560347_at | --- | --- | 0,00055325 | 0,000804392 | 0,642932 | -0,637261 | -1,55537 |
| 200967_at | 5479 | PPIB | 0,00055372 | 0,000804392 | 4,19648 | 2,06918 | 4,19648 |
| 207977_s_at | 1805 | DPT | 0,00055496 | 0,000804392 | 2,90652 | 1,53929 | 2,90652 |
| 209312_x_at | 3123 /// 3126 /// 3127 /// 100507709 /// 100507714 | HLA-DRB1 /// HLA-DRB4 /// HLA-DRB5 /// LOC100507709 /// LOC100507714 | 0,00055663 | 0,000804392 | 2,20672 | 1,14191 | 2,20672 |
| 201698_s_at | 8683 /// 283459 | GATC /// SRSF9 | 0,00055671 | 0,000804392 | 1,61559 | 0,692065 | 1,61559 |
| 201718_s_at | 2037 | EPB41L2 | 0,00055828 | 0,000804392 | 4,50694 | 2,17215 | 4,50694 |
| 203334_at | 1659 | DHX8 | 0,00055864 | 0,000804392 | 2,00545 | 1,00392 | 2,00545 |
| 205147_x_at | 4689 | NCF4 | 0,00056344 | 0,000804392 | 2,08191 | 1,05791 | 2,08191 |
| 200869_at | 6142 /// 26780 | RPL18A /// SNORA68 | 0,00056423 | 0,000804392 | 3,63629 | 1,86247 | 3,63629 |
| 208093_s_at | 81565 | NDEL1 | 0,00056736 | 0,000804392 | 1,57927 | 0,659257 | 1,57927 |
| 224925_at | 57580 | PREX1 | 0,00056904 | 0,000804392 | 1,81837 | 0,862649 | 1,81837 |
| 201001_s_at | 7335 /// 387521 /// 387522 | TMEM189 /// TMEM189-UBE2V1 /// UBE2V1 | 0,00056932 | 0,000804392 | 1,57997 | 0,659897 | 1,57997 |
| 215014_at | 3752 | KCND3 | 0,00057143 | 0,000804392 | 0,57118 | -0,807983 | -1,75076 |
| 203853_s_at | 9846 | GAB2 | 0,00057174 | 0,000804392 | 1,97209 | 0,979725 | 1,97209 |
| 211696_x_at | 3043 | HBB | 0,000572 | 0,000804392 | 4,16867 | 2,05959 | 4,16867 |
| 222067_x_at | 3017 | HIST1H2BD | 0,00057208 | 0,000804392 | 1,51475 | 0,59908 | 1,51475 |
| 211047_x_at | 1175 | AP2S1 | 0,00057297 | 0,000804392 | 2,05765 | 1,041 | 2,05765 |
| 200773_x_at | 5757 | PTMA | 0,00057346 | 0,000804392 | 1,62105 | 0,696925 | 1,62105 |
| 218644_at | 26499 | PLEK2 | 0,00057528 | 0,000804392 | 0,646459 | -0,629368 | -1,54689 |
| 212788_x_at | 2512 | FTL | 0,00057659 | 0,000804392 | 1,7676 | 0,821793 | 1,7676 |
| 239418_x_at | --- | --- | 0,0005807 | 0,00080793 | 0,649266 | -0,623119 | -1,5402 |
| 225818_s_at | 84897 | TBRG1 | 0,00058928 | 0,00081065 | 1,58472 | 0,664226 | 1,58472 |
| 203544_s_at | 8027 | STAM | 0,00058989 | 0,00081065 | 0,615547 | -0,700058 | -1,62457 |
| 218215_s_at | 7376 | NR1H2 | 0,00059034 | 0,00081065 | 1,90862 | 0,932533 | 1,90862 |
| 202605_at | 2990 | GUSB | 0,00059156 | 0,00081065 | 2,33222 | 1,22171 | 2,33222 |
| 221548_s_at | 80895 | ILKAP | 0,00059258 | 0,00081065 | 1,69362 | 0,760112 | 1,69362 |
| 221666_s_at | 29108 | PYCARD | 0,00059337 | 0,00081065 | 2,49315 | 1,31797 | 2,49315 |
| 212419_at | 219654 | ZCCHC24 | 0,00059374 | 0,00081065 | 1,66025 | 0,731399 | 1,66025 |
| 224825_at | 116092 | DNTTIP1 | 0,00059722 | 0,000813233 | 1,58185 | 0,661614 | 1,58185 |
| 201161_s_at | 8531 | YBX3 | 0,00060246 | 0,000818199 | 2,38687 | 1,25512 | 2,38687 |
| 241456_at | 149297 | FAM78B | 0,00060759 | 0,000821117 | 0,607669 | -0,718642 | -1,64563 |
| 201191_at | 5306 | PITPNA | 0,00060955 | 0,000821117 | 1,98898 | 0,992027 | 1,98898 |
| 224358_s_at | 58475 | MS4A7 | 0,00061109 | 0,000821117 | 3,25311 | 1,70182 | 3,25311 |
| 40148_at | 323 | APBB2 | 0,000612 | 0,000821117 | 1,5471 | 0,629562 | 1,5471 |
| 207393_at | 3062 | HCRTR2 | 0,00061588 | 0,000821117 | 1,56764 | 0,64859 | 1,56764 |
| 218227_at | 10101 | NUBP2 | 0,00061863 | 0,000821117 | 1,63794 | 0,711885 | 1,63794 |
| 239155_at | 1525 | CXADR | 0,00061868 | 0,000821117 | 0,47052 | -1,08767 | -2,12531 |
| 204710_s_at | 26100 | WIPI2 | 0,00061886 | 0,000821117 | 1,50233 | 0,587203 | 1,50233 |
| 218097_s_at | 79004 | CUEDC2 | 0,00062179 | 0,000821117 | 2,3387 | 1,2257 | 2,3387 |
| 211395_x_at | 9103 | FCGR2C | 0,00062182 | 0,000821117 | 2,45561 | 1,29608 | 2,45561 |
| 200704_at | 9516 | LITAF | 0,00062225 | 0,000821117 | 2,84382 | 1,50783 | 2,84382 |
| 223539_s_at | 8293 /// 728492 | SERF1A /// SERF1B | 0,0006241 | 0,000821443 | 2,46997 | 1,30449 | 2,46997 |
| 208894_at | 3122 | HLA-DRA | 0,00063255 | 0,000828574 | 2,89301 | 1,53257 | 2,89301 |
| 206543_at | 6595 | SMARCA2 | 0,00063315 | 0,000828574 | 0,461074 | -1,11693 | -2,16885 |
| 1566302_at | 6992 | PPP1R11 | 0,00063438 | 0,000828574 | 0,663217 | -0,592446 | -1,5078 |
| 200805_at | 10960 | LMAN2 | 0,00063747 | 0,000830498 | 1,80737 | 0,853894 | 1,80737 |
| 212974_at | 22898 | DENND3 | 0,00064349 | 0,000833535 | 1,50026 | 0,58521 | 1,50026 |
| 1561103_at | --- | OTTHUMG00000008327 /// RP11-378I13.1 | 0,00064477 | 0,000833535 | 0,501439 | -0,995854 | -1,99426 |
| 201137_s_at | 3115 | HLA-DPB1 | 0,00064523 | 0,000833535 | 4,23779 | 2,08331 | 4,23779 |
| 201422_at | 5296 /// 10437 | IFI30 /// PIK3R2 | 0,00064782 | 0,000833535 | 2,21576 | 1,1478 | 2,21576 |
| 203489_at | 10572 | SIVA1 | 0,00064924 | 0,000833535 | 2,12396 | 1,08676 | 2,12396 |
| 204298_s_at | 4015 | LOX | 0,00065108 | 0,000833535 | 1,8086 | 0,854871 | 1,8086 |
| 213616_at | 25941 | TPGS2 | 0,0006512 | 0,000833535 | 1,62358 | 0,699178 | 1,62358 |
| 239504_at | --- | --- | 0,00065319 | 0,000834003 | 0,422581 | -1,2427 | -2,36641 |
| 221088_s_at | 55607 | PPP1R9A | 0,0006573 | 0,000835266 | 0,569977 | -0,811024 | -1,75446 |
| 220786_s_at | 55089 | SLC38A4 | 0,00065745 | 0,000835266 | 0,602157 | -0,731788 | -1,6607 |
| 236024_at | 2823 | GPM6A | 0,00066387 | 0,000840588 | 0,604056 | -0,727247 | -1,65548 |
| 232481_s_at | 84189 | SLITRK6 | 0,00066492 | 0,000840588 | 0,381202 | -1,39137 | -2,62328 |
| 202465_at | 5118 | PCOLCE | 0,00066851 | 0,000843052 | 1,72421 | 0,785939 | 1,72421 |
| 203219_s_at | 353 | APRT | 0,0006725 | 0,000844237 | 1,62416 | 0,699695 | 1,62416 |
| 223952_x_at | 10170 | DHRS9 | 0,00067275 | 0,000844237 | 2,25202 | 1,17122 | 2,25202 |
| 213897_s_at | 6150 | MRPL23 | 0,00067644 | 0,000845467 | 1,90809 | 0,93213 | 1,90809 |
| 202839_s_at | 4713 | NDUFB7 | 0,00067703 | 0,000845467 | 1,96735 | 0,976255 | 1,96735 |
| 224992_s_at | 80790 | CMIP | 0,00068181 | 0,000849363 | 1,71588 | 0,778949 | 1,71588 |
| 230681_at | 84897 | TBRG1 | 0,00068962 | 0,000856552 | 0,649706 | -0,622142 | -1,53916 |
| 222921_s_at | 23493 | HEY2 | 0,00069093 | 0,000856552 | 1,61551 | 0,691986 | 1,61551 |
| 203746_s_at | 3052 | HCCS | 0,00069389 | 0,000858147 | 1,55739 | 0,639134 | 1,55739 |
| 214234_s_at | 1577 | CYP3A5 | 0,00070012 | 0,000863765 | 0,6413 | -0,640928 | -1,55933 |
| 201379_s_at | 7165 | TPD52L2 | 0,00070396 | 0,00086641 | 1,75071 | 0,807941 | 1,75071 |
| 204238_s_at | 10591 | DNPH1 | 0,00070807 | 0,000866441 | 1,77073 | 0,824343 | 1,77073 |
| 224064_s_at | 79947 | DHDDS | 0,00070853 | 0,000866441 | 2,22279 | 1,15237 | 2,22279 |
| 204985_s_at | 79090 | TRAPPC6A | 0,00070906 | 0,000866441 | 1,87851 | 0,909591 | 1,87851 |
| 213892_s_at | 353 | APRT | 0,00071563 | 0,000872382 | 1,72413 | 0,785866 | 1,72413 |
| 219952_s_at | 57192 | MCOLN1 | 0,00071741 | 0,000872475 | 1,59598 | 0,674445 | 1,59598 |
| 217860_at | 4705 | NDUFA10 | 0,00072118 | 0,000874985 | 1,80997 | 0,855963 | 1,80997 |
| 240874_at | --- | --- | 0,000723 | 0,000875124 | 0,534733 | -0,903109 | -1,87009 |
| 231880_at | 57464 | STRIP2 | 0,0007292 | 0,000878896 | 0,54175 | -0,884301 | -1,84587 |
| 203575_at | 1459 | CSNK2A2 | 0,00072955 | 0,000878896 | 1,87943 | 0,910294 | 1,87943 |
| 200868_s_at | 55905 | RNF114 | 0,00073187 | 0,000879622 | 3,16037 | 1,66009 | 3,16037 |
| 208907_s_at | 28973 | MRPS18B | 0,00073468 | 0,00088093 | 2,30138 | 1,2025 | 2,30138 |
| 202965_s_at | 827 | CAPN6 | 0,00073867 | 0,000883494 | 0,488252 | -1,0343 | -2,04812 |
| 225304_s_at | 2527 /// 126328 | FUT5 /// NDUFA11 | 0,00074027 | 0,000883494 | 3,31302 | 1,72815 | 3,31302 |
| 223359_s_at | 5150 | PDE7A | 0,00074384 | 0,000885683 | 0,553358 | -0,853714 | -1,80715 |
| 220934_s_at | 79064 | TMEM223 | 0,00074836 | 0,000888999 | 1,57963 | 0,659584 | 1,57963 |
| 209606_at | 9595 | CYTIP | 0,0007636 | 0,000902542 | 2,34245 | 1,22802 | 2,34245 |
| 215039_at | 339524 | LOC339524 | 0,0007647 | 0,000902542 | 2,51644 | 1,33138 | 2,51644 |
| 221267_s_at | 81926 | ABHD17A | 0,00076505 | 0,000902542 | 1,91216 | 0,935204 | 1,91216 |
| 237361_at | --- | --- | 0,00076763 | 0,000903487 | 0,641517 | -0,640441 | -1,55881 |
| 239489_at | --- | --- | 0,00077406 | 0,000903487 | 0,581913 | -0,781125 | -1,71847 |
| 204712_at | 11197 | WIF1 | 0,00077561 | 0,000903487 | 0,406482 | -1,29874 | -2,46013 |
| 225329_at | 348262 | FAM195B | 0,0007782 | 0,000903487 | 1,83543 | 0,876116 | 1,83543 |
| 207831_x_at | 1725 | DHPS | 0,00077905 | 0,000903487 | 1,70701 | 0,771473 | 1,70701 |
| 216682_s_at | 55578 | SUPT20H | 0,00078107 | 0,000903487 | 0,605227 | -0,724453 | -1,65227 |
| 222460_s_at | 80011 | FAM192A | 0,00078162 | 0,000903487 | 2,13294 | 1,09285 | 2,13294 |
| 1554918_a_at | 10257 | ABCC4 | 0,00078263 | 0,000903487 | 2,21322 | 1,14614 | 2,21322 |
| 201245_s_at | 55611 | OTUB1 | 0,00078414 | 0,000903487 | 1,59059 | 0,669564 | 1,59059 |
| 226319_s_at | 10189 | ALYREF | 0,00078477 | 0,000903487 | 2,37502 | 1,24794 | 2,37502 |
| 230337_at | 6654 | SOS1 | 0,00078526 | 0,000903487 | 0,457055 | -1,12956 | -2,18792 |
| 205681_at | 597 | BCL2A1 | 0,00079244 | 0,000908621 | 3,9417 | 1,97882 | 3,9417 |
| 240634_x_at | --- | --- | 0,00079543 | 0,000908621 | 0,57971 | -0,786598 | -1,725 |
| 1561777_at | --- | --- | 0,00079725 | 0,000908621 | 0,506537 | -0,981259 | -1,97419 |
| 236261_at | 114880 | OSBPL6 | 0,00079828 | 0,000908621 | 0,614484 | -0,702554 | -1,62738 |
| 212742_at | 27246 /// 101060478 | LOC101060478 /// RNF115 | 0,00079859 | 0,000908621 | 1,96704 | 0,976027 | 1,96704 |
| 203932_at | 3109 | HLA-DMB | 0,00080464 | 0,000912337 | 2,30381 | 1,20402 | 2,30381 |
| 236472_at | --- | --- | 0,00080804 | 0,000912337 | 0,540483 | -0,88768 | -1,8502 |
| 206099_at | 5583 | PRKCH | 0,00080956 | 0,000912337 | 1,76099 | 0,816389 | 1,76099 |
| 210075_at | 51257 | 2.Mar | 0,00081142 | 0,000912337 | 2,264 | 1,17888 | 2,264 |
| 1554769_at | 146540 | ZNF785 | 0,00081307 | 0,000912337 | 0,596343 | -0,745786 | -1,67689 |
| 223922_x_at | 64231 | MS4A6A | 0,00081396 | 0,000912337 | 2,30396 | 1,20411 | 2,30396 |
| 224885_s_at | 200185 | KRTCAP2 | 0,00081433 | 0,000912337 | 2,54008 | 1,34487 | 2,54008 |
| 234074_at | --- | --- | 0,00081619 | 0,000912421 | 0,472394 | -1,08194 | -2,11688 |
| 1568594_s_at | 84851 | TRIM52 | 0,00082042 | 0,000913645 | 0,631637 | -0,662832 | -1,58319 |
| 208937_s_at | 3397 | ID1 | 0,00082085 | 0,000913645 | 3,39623 | 1,76393 | 3,39623 |
| 210992_x_at | 9103 | FCGR2C | 0,00082447 | 0,00091478 | 2,29426 | 1,19803 | 2,29426 |
| 212891_s_at | 90480 | GADD45GIP1 | 0,00082554 | 0,00091478 | 1,90906 | 0,932859 | 1,90906 |
| 211327_x_at | 3077 | HFE | 0,00082723 | 0,00091478 | 1,51015 | 0,594688 | 1,51015 |
| 202483_s_at | 5902 | RANBP1 | 0,00083136 | 0,000917367 | 1,57474 | 0,655111 | 1,57474 |
| 209592_s_at | 10238 | DCAF7 | 0,00083471 | 0,000919082 | 1,71065 | 0,774544 | 1,71065 |
| 210982_s_at | 3122 | HLA-DRA | 0,00084582 | 0,00092741 | 5,08484 | 2,3462 | 5,08484 |
| 213566_at | 6039 | RNASE6 | 0,00084629 | 0,00092741 | 2,00684 | 1,00493 | 2,00684 |
| 224009_x_at | 10170 | DHRS9 | 0,00084771 | 0,00092741 | 2,30424 | 1,20429 | 2,30424 |
| 225602_at | 152007 | GLIPR2 | 0,0008522 | 0,000928363 | 2,20225 | 1,13898 | 2,20225 |
| 203582_s_at | 5867 /// 10638 | RAB4A /// SPHAR | 0,00085221 | 0,000928363 | 2,09225 | 1,06505 | 2,09225 |
| 203847_s_at | 10270 | AKAP8 | 0,00085793 | 0,000930118 | 1,59124 | 0,67015 | 1,59124 |
| 218327_s_at | 9342 | SNAP29 | 0,00085835 | 0,000930118 | 1,65406 | 0,726013 | 1,65406 |
| 211787_s_at | 1973 /// 26781 /// 652965 /// 652966 /// 100533955 | EIF4A1 /// SENP3-EIF4A1 /// SNORA48 /// SNORA67 /// SNORD10 | 0,00085973 | 0,000930118 | 1,71337 | 0,776836 | 1,71337 |
| 238596_at | 118924 | FRA10AC1 | 0,00086123 | 0,000930118 | 0,506788 | -0,980547 | -1,97321 |
| 238983_at | 79730 | NSUN7 | 0,00086356 | 0,000930118 | 0,423739 | -1,23875 | -2,35994 |
| 221207_s_at | 26960 | NBEA | 0,00086673 | 0,000930118 | 0,57448 | -0,799671 | -1,7407 |
| 203922_s_at | 1536 | CYBB | 0,00086793 | 0,000930118 | 2,10504 | 1,07385 | 2,10504 |
| 235960_at | 8087 | FXR1 | 0,00086861 | 0,000930118 | 0,58394 | -0,776107 | -1,7125 |
| 211513_s_at | 11054 | OGFR | 0,00087095 | 0,000930118 | 1,56913 | 0,649962 | 1,56913 |
| 225792_at | 51361 | HOOK1 | 0,00087377 | 0,000930118 | 0,34341 | -1,54199 | -2,91197 |
| 202348_s_at | 1861 | TOR1A | 0,0008738 | 0,000930118 | 1,54163 | 0,62446 | 1,54163 |
| 208803_s_at | 6731 | SRP72 | 0,0008765 | 0,000930885 | 1,55072 | 0,632938 | 1,55072 |
| 242011_at | --- | --- | 0,00087854 | 0,000930885 | 0,632905 | -0,659938 | -1,58002 |
| 225353_s_at | 714 | C1QC | 0,00088112 | 0,000930885 | 2,21746 | 1,14891 | 2,21746 |
| 218765_at | 51092 | SIDT2 | 0,0008818 | 0,000930885 | 1,71098 | 0,774826 | 1,71098 |
| 213068_at | 1805 | DPT | 0,00089061 | 0,000935148 | 2,3545 | 1,23542 | 2,3545 |
| 1566785_x_at | 4905 | NSF | 0,00089244 | 0,000935148 | 0,4254 | -1,23311 | -2,35073 |
| 213095_x_at | 199 | AIF1 | 0,00089262 | 0,000935148 | 1,75371 | 0,810412 | 1,75371 |
| 214109_at | 987 | LRBA | 0,00089536 | 0,000935148 | 0,551713 | -0,85801 | -1,81254 |
| 1566462_at | --- | --- | 0,00089619 | 0,000935148 | 0,619985 | -0,689695 | -1,61294 |
| 227864_s_at | 93343 | MVB12A | 0,00089972 | 0,000935148 | 2,01903 | 1,01366 | 2,01903 |
| 232784_at | --- | --- | 0,00090243 | 0,000935148 | 1,53066 | 0,614151 | 1,53066 |
| 228500_at | 199745 | THAP8 | 0,00090337 | 0,000935148 | 1,58589 | 0,665289 | 1,58589 |
| 204464_s_at | 1909 | EDNRA | 0,00090571 | 0,000935148 | 0,577646 | -0,791741 | -1,73116 |
| 202723_s_at | 2308 | FOXO1 | 0,00090806 | 0,000935148 | 2,25549 | 1,17344 | 2,25549 |
| 222912_at | 408 | ARRB1 | 0,00090816 | 0,000935148 | 1,86945 | 0,902611 | 1,86945 |
| 218231_at | 55577 | NAGK | 0,00090896 | 0,000935148 | 1,93746 | 0,954165 | 1,93746 |
| 217990_at | 51292 | GMPR2 | 0,00090958 | 0,000935148 | 1,76262 | 0,817721 | 1,76262 |
| 213062_at | 123803 | NTAN1 | 0,00091558 | 0,000939428 | 1,71588 | 0,778947 | 1,71588 |
| 244023_at | 6850 | SYK | 0,00092577 | 0,000947985 | 0,659048 | -0,601546 | -1,51734 |
| 202041_s_at | 9158 | FIBP | 0,00093249 | 0,000952963 | 1,89728 | 0,923935 | 1,89728 |
| 201106_at | 2879 | GPX4 | 0,00094098 | 0,000958106 | 2,90205 | 1,53707 | 2,90205 |
| 220005_at | 53829 | P2RY13 | 0,00094126 | 0,000958106 | 2,38377 | 1,25324 | 2,38377 |
| 224909_s_at | 57580 | PREX1 | 0,0009453 | 0,0009603 | 1,95133 | 0,964454 | 1,95133 |
| 208612_at | 2923 | PDIA3 | 0,00095123 | 0,000964415 | 3,6232 | 1,85726 | 3,6232 |
| 225797_at | 116541 | MRPL54 | 0,00095429 | 0,000965602 | 3,59048 | 1,84418 | 3,59048 |
| 204006_s_at | 2214 /// 2215 | FCGR3A /// FCGR3B | 0,00096364 | 0,000973142 | 6,75316 | 2,75556 | 6,75316 |
| 203761_at | 6503 | SLA | 0,00096647 | 0,000973398 | 2,18184 | 1,12554 | 2,18184 |
| 225245_x_at | 55766 | H2AFJ | 0,00096769 | 0,000973398 | 2,16498 | 1,11435 | 2,16498 |
| 219350_s_at | 56616 | DIABLO | 0,00097385 | 0,000974879 | 1,91029 | 0,93379 | 1,91029 |
| 231990_at | 9958 | USP15 | 0,00097453 | 0,000974879 | 1,54245 | 0,62522 | 1,54245 |
| 229506_at | 151742 | PPM1L | 0,00097488 | 0,000974879 | 0,491558 | -1,02457 | -2,03435 |
