# Supplementary material for: Molecular Signatures of Human Chronic Atrial Fibrillation in Primary Mitral Regurgitation
Source: Cardiovasc Ther. 2021 Oct 15;2021:5516185. doi: 10.1155/2021/5516185 (PMC8538404; doi:10.1155/2021/5516185)
Supplement: Supplementary 5 — Supplementary Table 4: all differentially expressed genes in all LAA tissues of AFib vs. SR using GSE2240 dataset (fold change > 1.5; P < 0.001 and q < 0.05). [file 5516185.f5.docx]

**Supplementary Table 4:** All differentially expressed genes in all LAA tissues of AFib vs SR using GSE2240 dataset (Fold Change > 1.5; p<0.001 and q<0.05).

| **Probeset ID** | **Entrez Gene** | **Gene Symbol** | **p-value** | **q value** | **MeanRatio(AF/SR)** | **MeanDiff(AF-SR)** | **FoldChange(AF/SR)** |
| --- | --- | --- | --- | --- | --- | --- | --- |
| 201149_s_at | 7078 | TIMP3 | 2,24E-11 | 6,19162E-09 | 0,481565 | -1,0542 | -2,07656 |
| 201147_s_at | 7078 | TIMP3 | 5,42E-11 | 7,48642E-09 | 0,568579 | -0,814568 | -1,75877 |
| 201148_s_at | 7078 | TIMP3 | 7,90E-10 | 7,27007E-08 | 0,613512 | -0,704837 | -1,62996 |
| 217889_s_at | 79901 | CYBRD1 | 4,70E-09 | 3,24323E-07 | 0,439876 | -1,18483 | -2,27337 |
| 221288_at | 2845 | GPR22 | 5,91E-09 | 3,26163E-07 | 0,459109 | -1,12309 | -2,17813 |
| 203630_s_at | 10466 | COG5 | 1,01E-08 | 4,66472E-07 | 0,557137 | -0,843897 | -1,79489 |
| 204260_at | 1114 | CHGB | 2,23E-08 | 8,799E-07 | 2,36316 | 1,24072 | 2,36316 |
| 219799_s_at | 10170 | DHRS9 | 3,34E-08 | 1,12778E-06 | 2,43434 | 1,28353 | 2,43434 |
| 206768_at | 6123 | RPL3L | 3,68E-08 | 1,12778E-06 | 1,63104 | 0,705788 | 1,63104 |
| 221795_at | 4915 | NTRK2 | 4,50E-08 | 1,24297E-06 | 0,398576 | -1,32707 | -2,50893 |
| 207468_s_at | 6425 | SFRP5 | 5,71E-08 | 1,43236E-06 | 0,400654 | -1,31957 | -2,49592 |
| 209693_at | 23245 | ASTN2 | 7,61E-08 | 1,62123E-06 | 0,466422 | -1,10029 | -2,14398 |
| 201752_s_at | 120 | ADD3 | 7,64E-08 | 1,62123E-06 | 0,574554 | -0,799485 | -1,74048 |
| 205253_at | 5087 | PBX1 | 1,67E-07 | 3,11926E-06 | 0,650678 | -0,619985 | -1,53686 |
| 204755_x_at | 3131 | HLF | 1,70E-07 | 3,11926E-06 | 0,591602 | -0,757301 | -1,69033 |
| 204753_s_at | 3131 | HLF | 1,99E-07 | 3,42234E-06 | 0,538147 | -0,893928 | -1,85823 |
| 216235_s_at | 1909 | EDNRA | 2,11E-07 | 3,42234E-06 | 0,623291 | -0,682023 | -1,60439 |
| 203940_s_at | 22846 | VASH1 | 2,32E-07 | 3,56413E-06 | 1,58928 | 0,668374 | 1,58928 |
| 209921_at | 23657 | SLC7A11 | 3,02E-07 | 4,31885E-06 | 0,477443 | -1,0666 | -2,09449 |
| 212057_at | 23199 | GSE1 | 3,13E-07 | 4,31885E-06 | 0,542297 | -0,882845 | -1,84401 |
| 201718_s_at | 2037 | EPB41L2 | 3,72E-07 | 4,78766E-06 | 0,615573 | -0,699999 | -1,6245 |
| 224009_x_at | 10170 | DHRS9 | 3,82E-07 | 4,78766E-06 | 2,38418 | 1,25349 | 2,38418 |
| 230577_at | 100507008 | LINC00844 | 4,52E-07 | 5,42802E-06 | 0,482692 | -1,05082 | -2,07171 |
| 201150_s_at | 7078 | TIMP3 | 4,97E-07 | 5,71318E-06 | 0,651336 | -0,618526 | -1,53531 |
| 226905_at | 359845 | FAM101B | 5,26E-07 | 5,80537E-06 | 1,57542 | 0,655736 | 1,57542 |
| 214680_at | 4915 | NTRK2 | 5,89E-07 | 6,25256E-06 | 0,582786 | -0,778961 | -1,71589 |
| 202718_at | 3485 | IGFBP2 | 6,25E-07 | 6,39298E-06 | 2,67719 | 1,42072 | 2,67719 |
| 202995_s_at | 2192 | FBLN1 | 7,62E-07 | 7,3106E-06 | 0,536451 | -0,898483 | -1,8641 |
| 201753_s_at | 120 | ADD3 | 7,68E-07 | 7,3106E-06 | 0,642335 | -0,638603 | -1,55682 |
| 207334_s_at | 7048 | TGFBR2 | 9,68E-07 | 8,65321E-06 | 0,588022 | -0,766058 | -1,70062 |
| 244655_at | 100507311 | LOC100507311 | 9,72E-07 | 8,65321E-06 | 0,648617 | -0,62456 | -1,54174 |
| 205493_s_at | 10570 | DPYSL4 | 1,01E-06 | 8,70073E-06 | 2,15821 | 1,10984 | 2,15821 |
| 221503_s_at | 3839 | KPNA3 | 1,13E-06 | 9,46655E-06 | 0,59687 | -0,744511 | -1,67541 |
| 204633_s_at | 9252 | RPS6KA5 | 1,22E-06 | 9,76704E-06 | 0,660752 | -0,597819 | -1,51343 |
| 206073_at | 8292 | COLQ | 1,28E-06 | 9,76704E-06 | 1,90413 | 0,92913 | 1,90413 |
| 210147_at | 419 | ART3 | 1,30E-06 | 9,76704E-06 | 0,62611 | -0,675511 | -1,59716 |
| 208370_s_at | 1827 | RCAN1 | 1,31E-06 | 9,76704E-06 | 1,7304 | 0,791108 | 1,7304 |
| 215407_s_at | 23245 | ASTN2 | 1,38E-06 | 1,00049E-05 | 0,504123 | -0,988153 | -1,98364 |
| 231964_at | 636 | BICD1 | 1,56E-06 | 1,10152E-05 | 1,61401 | 0,690647 | 1,61401 |
| 223007_s_at | 23731 | TMEM245 | 1,66E-06 | 1,11075E-05 | 0,622479 | -0,683904 | -1,60648 |
| 203423_at | 5947 | RBP1 | 1,68E-06 | 1,11075E-05 | 0,62196 | -0,685107 | -1,60782 |
| 210198_s_at | 5354 | PLP1 | 1,72E-06 | 1,11075E-05 | 0,549402 | -0,864067 | -1,82016 |
| 223952_x_at | 10170 | DHRS9 | 1,73E-06 | 1,11075E-05 | 2,21366 | 1,14643 | 2,21366 |
| 203629_s_at | 10466 | COG5 | 2,00E-06 | 1,25162E-05 | 0,629896 | -0,666814 | -1,58756 |
| 222108_at | 347902 | AMIGO2 | 2,14E-06 | 1,31281E-05 | 0,663146 | -0,592602 | -1,50796 |
| 212224_at | 216 | ALDH1A1 | 2,24E-06 | 1,33763E-05 | 0,494293 | -1,01656 | -2,02309 |
| 204591_at | 10752 | CHL1 | 2,28E-06 | 1,33763E-05 | 0,513025 | -0,962899 | -1,94922 |
| 227566_at | 50863 /// 100653217 | LOC100653217 /// NTM | 2,47E-06 | 1,42268E-05 | 0,394949 | -1,34026 | -2,53197 |
| 209612_s_at | 125 | ADH1B | 2,67E-06 | 1,50299E-05 | 0,580156 | -0,785488 | -1,72368 |
| 201787_at | 2192 | FBLN1 | 2,73E-06 | 1,50936E-05 | 0,558917 | -0,839294 | -1,78917 |
| 213001_at | 23452 | ANGPTL2 | 2,92E-06 | 1,57887E-05 | 1,60848 | 0,685701 | 1,60848 |
| 201433_s_at | 9791 | PTDSS1 | 3,25E-06 | 1,72757E-05 | 0,666246 | -0,585874 | -1,50095 |
| 211355_x_at | 3953 | LEPR | 3,59E-06 | 1,77428E-05 | 0,514045 | -0,960032 | -1,94535 |
| 211813_x_at | 1634 | DCN | 3,62E-06 | 1,77428E-05 | 0,658716 | -0,602272 | -1,51811 |
| 227819_at | 59352 | LGR6 | 3,71E-06 | 1,77428E-05 | 0,647756 | -0,626477 | -1,54379 |
| 211356_x_at | 3953 | LEPR | 3,71E-06 | 1,77428E-05 | 0,491507 | -1,02472 | -2,03456 |
| 202994_s_at | 2192 | FBLN1 | 3,72E-06 | 1,77428E-05 | 0,595363 | -0,74816 | -1,67965 |
| 214040_s_at | 2934 | GSN | 3,73E-06 | 1,77428E-05 | 0,462083 | -1,11378 | -2,16411 |
| 219679_s_at | 51322 | WAC | 3,89E-06 | 1,82185E-05 | 0,623601 | -0,681305 | -1,60359 |
| 229580_at | --- | OTTHUMG00000175814 /// RP11-13L2.4 | 4,46E-06 | 2,04971E-05 | 0,60365 | -0,728216 | -1,65659 |
| 205782_at | 2252 | FGF7 | 4,73E-06 | 2,06964E-05 | 0,658936 | -0,601789 | -1,5176 |
| 210625_s_at | 8165 | AKAP1 | 4,79E-06 | 2,06964E-05 | 0,626426 | -0,674784 | -1,59636 |
| 243737_at | 23439 | ATP1B4 | 4,82E-06 | 2,06964E-05 | 2,46466 | 1,30139 | 2,46466 |
| 205478_at | 5502 | PPP1R1A | 4,83E-06 | 2,06964E-05 | 0,495034 | -1,0144 | -2,02006 |
| 217966_s_at | 116496 | FAM129A | 4,95E-06 | 2,06964E-05 | 0,517472 | -0,950446 | -1,93247 |
| 235849_at | 286133 | SCARA5 | 4,95E-06 | 2,06964E-05 | 0,586787 | -0,769091 | -1,7042 |
| 217813_s_at | 10927 | SPIN1 | 5,32E-06 | 2,19349E-05 | 0,6363 | -0,652222 | -1,57159 |
| 202311_s_at | 1277 | COL1A1 | 5,44E-06 | 2,20744E-05 | 1,8505 | 0,887917 | 1,8505 |
| 227561_at | 4921 | DDR2 | 6,24E-06 | 2,49518E-05 | 0,587262 | -0,767923 | -1,70282 |
| 202350_s_at | 4147 /// 100506558 | LOC100506558 /// MATN2 | 6,49E-06 | 2,55903E-05 | 0,579582 | -0,786914 | -1,72538 |
| 214961_at | 23281 | MTUS2 | 7,26E-06 | 2,79155E-05 | 0,570464 | -0,809791 | -1,75296 |
| 209616_s_at | 1066 /// 100653057 | CES1 /// LOC100653057 | 7,28E-06 | 2,79155E-05 | 0,515385 | -0,956279 | -1,9403 |
| 205854_at | 7289 | TULP3 | 7,97E-06 | 3,01386E-05 | 0,601982 | -0,732209 | -1,66118 |
| 214305_s_at | 23451 | SF3B1 | 9,61E-06 | 3,57159E-05 | 0,646862 | -0,628469 | -1,54592 |
| 209590_at | 655 | BMP7 | 9,71E-06 | 3,57159E-05 | 0,658911 | -0,601844 | -1,51766 |
| 223500_at | 10815 | CPLX1 | 1,05E-05 | 0,000038088 | 1,76754 | 0,821744 | 1,76754 |
| 211110_s_at | 367 | AR | 1,06E-05 | 0,000038088 | 0,552745 | -0,855313 | -1,80915 |
| 229802_at | 8840 | WISP1 | 1,09E-05 | 0,000038732 | 1,51567 | 0,599955 | 1,51567 |
| 211354_s_at | 3953 | LEPR | 1,17E-05 | 4,07411E-05 | 0,557061 | -0,844092 | -1,79513 |
| 201151_s_at | 4154 | MBNL1 | 1,30E-05 | 4,46255E-05 | 0,592713 | -0,754595 | -1,68716 |
| 222020_s_at | 50863 /// 100653217 | LOC100653217 /// NTM | 1,31E-05 | 4,46255E-05 | 0,553911 | -0,852274 | -1,80534 |
| 212713_at | 4239 | MFAP4 | 1,40E-05 | 4,72509E-05 | 0,581532 | -0,782069 | -1,7196 |
| 204719_at | 10351 | ABCA8 | 1,43E-05 | 4,72818E-05 | 0,609836 | -0,713506 | -1,63978 |
| 212188_at | 115207 | KCTD12 | 1,44E-05 | 4,72818E-05 | 0,579104 | -0,788107 | -1,72681 |
| 203803_at | 51449 | PCYOX1 | 1,59E-05 | 5,14615E-05 | 0,540551 | -0,887498 | -1,84997 |
| 212912_at | 6196 | RPS6KA2 | 1,61E-05 | 5,14615E-05 | 1,52537 | 0,609162 | 1,52537 |
| 213004_at | 23452 | ANGPTL2 | 1,63E-05 | 5,14615E-05 | 1,54967 | 0,631957 | 1,54967 |
| 212151_at | 5087 | PBX1 | 1,64E-05 | 5,14615E-05 | 0,626659 | -0,674247 | -1,59576 |
| 241706_at | 144402 | CPNE8 | 1,68E-05 | 5,2248E-05 | 0,5663 | -0,820361 | -1,76585 |
| 204811_s_at | 9254 | CACNA2D2 | 1,80E-05 | 5,51614E-05 | 0,597049 | -0,744078 | -1,6749 |
| 220037_s_at | 10894 | LYVE1 | 1,90E-05 | 5,77249E-05 | 0,386857 | -1,37013 | -2,58494 |
| 209894_at | 3953 | LEPR | 1,95E-05 | 5,79045E-05 | 0,51657 | -0,952965 | -1,93585 |
| 241753_at | --- | --- | 1,95E-05 | 5,79045E-05 | 0,454075 | -1,139 | -2,20228 |
| 225061_at | 55466 | DNAJA4 | 2,13E-05 | 6,24703E-05 | 1,56035 | 0,641867 | 1,56035 |
| 226663_at | 100505494 | ANKRD10-IT1 | 2,40E-05 | 6,93082E-05 | 1,5153 | 0,599606 | 1,5153 |
| 220484_at | 55283 | MCOLN3 | 2,41E-05 | 6,93082E-05 | 0,536332 | -0,898803 | -1,86452 |
| 211896_s_at | 1634 | DCN | 2,51E-05 | 7,13457E-05 | 0,652256 | -0,61649 | -1,53314 |
| 211068_x_at | 253725 | FAM21C | 2,56E-05 | 7,21791E-05 | 0,611028 | -0,710689 | -1,63658 |
| 214589_at | 2257 | FGF12 | 2,68E-05 | 7,46452E-05 | 0,620093 | -0,689444 | -1,61266 |
| 229530_at | 2982 | GUCY1A3 | 2,74E-05 | 7,49119E-05 | 0,492135 | -1,02287 | -2,03196 |
| 224989_at | --- | --- | 2,74E-05 | 7,49119E-05 | 0,631561 | -0,663007 | -1,58338 |
| 209355_s_at | 8613 | PPAP2B | 2,78E-05 | 7,51507E-05 | 0,462577 | -1,11223 | -2,1618 |
| 201917_s_at | 55186 | SLC25A36 | 2,83E-05 | 7,59429E-05 | 0,452908 | -1,14271 | -2,20795 |
| 225681_at | 115908 | CTHRC1 | 2,89E-05 | 7,62259E-05 | 2,04772 | 1,03402 | 2,04772 |
| 204312_x_at | 1385 | CREB1 | 2,90E-05 | 7,62259E-05 | 0,627299 | -0,672774 | -1,59414 |
| 207764_s_at | 10114 | HIPK3 | 2,96E-05 | 7,71196E-05 | 0,628299 | -0,670476 | -1,5916 |
| 209094_at | 23576 | DDAH1 | 3,27E-05 | 0,000083651 | 1,55694 | 0,638712 | 1,55694 |
| 201565_s_at | 3398 | ID2 | 3,27E-05 | 0,000083651 | 0,640319 | -0,643137 | -1,56172 |
| 210168_at | 729 | C6 | 3,34E-05 | 8,44831E-05 | 0,545583 | -0,87413 | -1,8329 |
| 236304_at | --- | --- | 3,47E-05 | 8,70647E-05 | 2,49175 | 1,31716 | 2,49175 |
| 202627_s_at | 5054 | SERPINE1 | 3,56E-05 | 8,84712E-05 | 1,58496 | 0,664451 | 1,58496 |
| 209613_s_at | 125 | ADH1B | 3,65E-05 | 8,99905E-05 | 0,568466 | -0,814855 | -1,75912 |
| 225835_at | 6558 | SLC12A2 | 3,69E-05 | 9,02171E-05 | 1,55041 | 0,632646 | 1,55041 |
| 203542_s_at | 687 | KLF9 | 3,96E-05 | 9,58935E-05 | 0,604688 | -0,725737 | -1,65374 |
| 206667_s_at | 9522 | SCAMP1 | 4,04E-05 | 9,68798E-05 | 0,665258 | -0,588015 | -1,50318 |
| 212488_at | 1289 | COL5A1 | 4,13E-05 | 9,72512E-05 | 1,52828 | 0,611907 | 1,52828 |
| 211726_s_at | 2327 | FMO2 | 4,14E-05 | 9,72512E-05 | 0,535223 | -0,901789 | -1,86838 |
| 204463_s_at | 1909 | EDNRA | 4,16E-05 | 9,72512E-05 | 0,604779 | -0,725521 | -1,6535 |
| 228547_at | 9378 | NRXN1 | 4,21E-05 | 9,76808E-05 | 0,5312 | -0,912674 | -1,88253 |
| 219338_s_at | 54839 | LRRC49 | 4,30E-05 | 0,000098992 | 0,529572 | -0,917101 | -1,88832 |
| 204313_s_at | 1385 | CREB1 | 4,37E-05 | 9,94813E-05 | 0,499652 | -1,001 | -2,00139 |
| 218967_s_at | 9317 | PTER | 4,44E-05 | 9,94813E-05 | 0,507801 | -0,977664 | -1,96927 |
| 205399_at | 9201 | DCLK1 | 4,47E-05 | 9,94813E-05 | 0,629463 | -0,667808 | -1,58866 |
| 243929_at | --- | --- | 4,47E-05 | 9,94813E-05 | 0,360333 | -1,4726 | -2,77521 |
| 201737_s_at | 10299 | 6.Mar | 4,91E-05 | 0,000108491 | 0,40294 | -1,31136 | -2,48176 |
| 210655_s_at | 2309 /// 2310 | FOXO3 /// FOXO3B | 5,00E-05 | 0,000109032 | 0,663927 | -0,590904 | -1,50619 |
| 201711_x_at | 5903 | RANBP2 | 5,09E-05 | 0,000109032 | 0,627699 | -0,671856 | -1,59312 |
| 205018_s_at | 10150 | MBNL2 | 5,10E-05 | 0,000109032 | 0,599147 | -0,739019 | -1,66904 |
| 225817_at | 84952 | CGNL1 | 5,13E-05 | 0,000109032 | 0,662001 | -0,595095 | -1,51057 |
| 204762_s_at | 2775 | GNAO1 | 5,17E-05 | 0,000109032 | 0,617965 | -0,694403 | -1,61821 |
| 218051_s_at | 64943 | NT5DC2 | 5,21E-05 | 0,000109032 | 0,606522 | -0,721367 | -1,64874 |
| 201645_at | 3371 | TNC | 5,21E-05 | 0,000109032 | 2,00277 | 1,002 | 2,00277 |
| 201313_at | 2026 | ENO2 | 5,32E-05 | 0,000110432 | 1,61369 | 0,69036 | 1,61369 |
| 217802_s_at | 64710 | NUCKS1 | 5,56E-05 | 0,000113853 | 0,600588 | -0,735553 | -1,66504 |
| 209170_s_at | 2824 | GPM6B | 5,57E-05 | 0,000113853 | 0,550456 | -0,8613 | -1,81667 |
| 226228_at | 361 | AQP4 | 5,72E-05 | 0,000115267 | 0,439627 | -1,18565 | -2,27466 |
| 201008_s_at | 10628 /// 101060503 | LOC101060503 /// TXNIP | 5,72E-05 | 0,000115267 | 0,654393 | -0,611771 | -1,52813 |
| 202998_s_at | 4017 | LOXL2 | 6,11E-05 | 0,000122109 | 1,83136 | 0,872914 | 1,83136 |
| 206389_s_at | 5139 | PDE3A | 6,26E-05 | 0,000123795 | 0,627049 | -0,67335 | -1,59477 |
| 222244_s_at | 55000 | TUG1 | 6,28E-05 | 0,000123795 | 0,630149 | -0,666234 | -1,58693 |
| 212370_x_at | 55747 /// 253725 /// 387680 | FAM21A /// FAM21B /// FAM21C | 6,50E-05 | 0,000127056 | 0,584673 | -0,774297 | -1,71036 |
| 214946_x_at | 55747 /// 253725 /// 387680 | FAM21A /// FAM21B /// FAM21C | 6,54E-05 | 0,000127056 | 0,601527 | -0,733299 | -1,66244 |
| 222919_at | 10345 | TRDN | 6,97E-05 | 0,000134618 | 0,578693 | -0,78913 | -1,72803 |
| 222927_s_at | 594855 | CPLX3 | 7,10E-05 | 0,00013606 | 0,641658 | -0,640123 | -1,55846 |
| 205528_s_at | 862 | RUNX1T1 | 7,71E-05 | 0,000146815 | 0,621016 | -0,687297 | -1,61026 |
| 201918_at | 55186 | SLC25A36 | 7,82E-05 | 0,000147879 | 0,658236 | -0,603323 | -1,51921 |
| 212226_s_at | 8613 | PPAP2B | 8,35E-05 | 0,000156826 | 0,514191 | -0,959624 | -1,9448 |
| 203789_s_at | 10512 | SEMA3C | 8,52E-05 | 0,000157844 | 0,596743 | -0,74482 | -1,67576 |
| 224237_at | --- | --- | 8,52E-05 | 0,000157844 | 0,587591 | -0,767116 | -1,70186 |
| 205738_s_at | 2170 | FABP3 | 8,67E-05 | 0,000159551 | 1,51044 | 0,594974 | 1,51044 |
| 228434_at | 153579 | BTNL9 | 8,86E-05 | 0,000162003 | 1,61885 | 0,69497 | 1,61885 |
| 225895_at | 171024 | SYNPO2 | 9,13E-05 | 0,000165694 | 0,495665 | -1,01256 | -2,01749 |
| 213342_at | 10413 | YAP1 | 9,81E-05 | 0,000176986 | 0,622367 | -0,684163 | -1,60677 |
| 204597_x_at | 6781 | STC1 | 9,91E-05 | 0,000177572 | 1,5305 | 0,614002 | 1,5305 |
| 213524_s_at | 50486 | G0S2 | 0,00010172 | 0,000181118 | 0,622477 | -0,683909 | -1,60649 |
| 226615_at | 9213 | XPR1 | 0,00010308 | 0,000181573 | 1,5322 | 0,615609 | 1,5322 |
| 208850_s_at | 7070 | THY1 | 0,00010329 | 0,000181573 | 1,6266 | 0,701857 | 1,6266 |
| 204364_s_at | 65055 | REEP1 | 0,00010503 | 0,000183465 | 0,593199 | -0,753412 | -1,68578 |
| 202628_s_at | 5054 | SERPINE1 | 0,00010775 | 0,000187034 | 1,77044 | 0,82411 | 1,77044 |
| 213156_at | --- | --- | 0,00010943 | 0,000188767 | 0,645936 | -0,630536 | -1,54814 |
| 206315_at | 9244 | CRLF1 | 0,00011187 | 0,000191772 | 1,79454 | 0,843613 | 1,79454 |
| 229461_x_at | 257194 | NEGR1 | 0,00011362 | 0,00019357 | 0,609741 | -0,713733 | -1,64004 |
| 212013_at | 7837 | PXDN | 0,00011577 | 0,000196033 | 1,5356 | 0,618807 | 1,5356 |
| 217678_at | 23657 | SLC7A11 | 0,00011774 | 0,000198146 | 0,643048 | -0,637001 | -1,55509 |
| 213272_s_at | 57146 | TMEM159 | 0,00012015 | 0,000200977 | 1,58425 | 0,663798 | 1,58425 |
| 213872_at | --- | --- | 0,00012223 | 0,000203227 | 0,570982 | -0,808484 | -1,75137 |
| 226035_at | 57478 | USP31 | 0,00012362 | 0,000204309 | 0,443159 | -1,1741 | -2,25653 |
| 226206_at | 7975 | MAFK | 0,00012686 | 0,000208415 | 1,59608 | 0,674536 | 1,59608 |
| 230793_at | 55604 | LRRC16A | 0,00012966 | 0,000211754 | 0,657345 | -0,605278 | -1,52127 |
| 214433_s_at | 8991 | SELENBP1 | 0,00013243 | 0,000215006 | 0,576474 | -0,794674 | -1,73468 |
| 219872_at | 51313 | FAM198B | 0,00013342 | 0,000215351 | 0,643685 | -0,635574 | -1,55356 |
| 226281_at | 92737 | DNER | 0,00013619 | 0,000218536 | 0,365466 | -1,45219 | -2,73623 |
| 226880_at | 64710 | NUCKS1 | 0,00014515 | 0,000230757 | 0,38693 | -1,36985 | -2,58445 |
| 227918_s_at | 79699 | ZYG11B | 0,00014548 | 0,000230757 | 0,530729 | -0,913952 | -1,8842 |
| 209897_s_at | 9353 | SLIT2 | 0,00014808 | 0,000233546 | 0,481885 | -1,05324 | -2,07518 |
| 213201_s_at | 7138 | TNNT1 | 0,00015026 | 0,000235635 | 1,62719 | 0,702386 | 1,62719 |
| 239348_at | --- | --- | 0,00015199 | 0,000236997 | 0,654694 | -0,611108 | -1,52743 |
| 203349_s_at | 2119 | ETV5 | 0,00015575 | 0,000241498 | 1,52145 | 0,605445 | 1,52145 |
| 225241_at | 151887 | CCDC80 | 0,0001572 | 0,000242393 | 0,660096 | -0,599252 | -1,51493 |
| 213016_at | 56987 | BBX | 0,00016674 | 0,000255665 | 0,661967 | -0,59517 | -1,51065 |
| 205177_at | 7135 | TNNI1 | 0,00017232 | 0,000259858 | 0,334491 | -1,57996 | -2,98962 |
| 211769_x_at | 10955 | SERINC3 | 0,00017248 | 0,000259858 | 0,636888 | -0,650887 | -1,57013 |
| 232541_at | --- | --- | 0,00017327 | 0,000259858 | 0,540499 | -0,887636 | -1,85014 |
| 202173_s_at | 7716 | VEZF1 | 0,00017366 | 0,000259858 | 0,473514 | -1,07852 | -2,11187 |
| 219221_at | 253461 | ZBTB38 | 0,00017462 | 0,000259858 | 0,55556 | -0,847986 | -1,79999 |
| 219059_s_at | 10894 | LYVE1 | 0,00017512 | 0,000259858 | 0,543214 | -0,880408 | -1,8409 |
| 204132_s_at | 2309 /// 2310 | FOXO3 /// FOXO3B | 0,00017734 | 0,000261748 | 0,656023 | -0,608181 | -1,52434 |
| 222404_x_at | 51495 | PTPLAD1 | 0,00017932 | 0,000263254 | 0,576021 | -0,795808 | -1,73605 |
| 221881_s_at | 25932 | CLIC4 | 0,00018203 | 0,00026582 | 0,659984 | -0,599497 | -1,51519 |
| 235308_at | 26137 | ZBTB20 | 0,00018518 | 0,000267613 | 0,471781 | -1,08381 | -2,11963 |
| 242059_at | --- | --- | 0,0001852 | 0,000267613 | 0,653687 | -0,613327 | -1,52978 |
| 209167_at | 2824 | GPM6B | 0,000192 | 0,000275718 | 0,497925 | -1,006 | -2,00833 |
| 202555_s_at | 4638 | MYLK | 0,0001928 | 0,000275718 | 0,518875 | -0,94654 | -1,92725 |
| 219737_s_at | 5101 | PCDH9 | 0,0002206 | 0,000313838 | 0,629218 | -0,668369 | -1,58927 |
| 209875_s_at | 6696 | SPP1 | 0,00022373 | 0,000316665 | 3,23169 | 1,69229 | 3,23169 |
| 202310_s_at | 1277 | COL1A1 | 0,00023146 | 0,000325933 | 1,868 | 0,901497 | 1,868 |
| 205382_s_at | 1675 | CFD | 0,00023601 | 0,000330652 | 0,5959 | -0,746859 | -1,67814 |
| 213998_s_at | 10521 | DDX17 | 0,00023897 | 0,000333114 | 0,577491 | -0,792129 | -1,73163 |
| 241208_at | 10611 | PDLIM5 | 0,00024076 | 0,000333509 | 0,603771 | -0,727926 | -1,65626 |
| 203697_at | 2487 | FRZB | 0,00024167 | 0,000333509 | 1,64944 | 0,721981 | 1,64944 |
| 213135_at | 7074 | TIAM1 | 0,00024619 | 0,000338045 | 0,577037 | -0,793265 | -1,73299 |
| 230958_s_at | --- | --- | 0,00025054 | 0,000342318 | 0,545187 | -0,875176 | -1,83423 |
| 211003_x_at | 7052 | TGM2 | 0,00025378 | 0,000345038 | 0,61548 | -0,700216 | -1,62475 |
| 210461_s_at | 3983 | ABLIM1 | 0,00025586 | 0,000346168 | 0,659691 | -0,600137 | -1,51586 |
| 202409_at | 3481 | IGF2 | 0,00026282 | 0,00035385 | 1,73556 | 0,7954 | 1,73556 |
| 221942_s_at | 2982 | GUCY1A3 | 0,00026853 | 0,000359783 | 0,642033 | -0,63928 | -1,55755 |
| 204864_s_at | 3572 | IL6ST | 0,00027999 | 0,00037174 | 0,570401 | -0,809952 | -1,75315 |
| 227501_at | --- | --- | 0,00028015 | 0,00037174 | 0,600614 | -0,73549 | -1,66496 |
| 233364_s_at | --- | --- | 0,00028459 | 0,000375824 | 0,603397 | -0,728821 | -1,65728 |
| 212007_at | 23190 | UBXN4 | 0,0002885 | 0,000379171 | 0,647057 | -0,628035 | -1,54546 |
| 204369_at | 5290 | PIK3CA | 0,00029189 | 0,000380432 | 0,572727 | -0,80408 | -1,74603 |
| 234000_s_at | 51495 | PTPLAD1 | 0,00029438 | 0,000380432 | 0,537563 | -0,895495 | -1,86025 |
| 221473_x_at | 10955 | SERINC3 | 0,00029488 | 0,000380432 | 0,660888 | -0,597523 | -1,51312 |
| 226094_at | 5286 | PIK3C2A | 0,00029497 | 0,000380432 | 0,618907 | -0,692206 | -1,61575 |
| 218330_s_at | 89797 | NAV2 | 0,0003039 | 0,000388991 | 1,57357 | 0,654037 | 1,57357 |
| 213456_at | 25928 | SOSTDC1 | 0,00030443 | 0,000388991 | 0,583163 | -0,778029 | -1,71479 |
| 211573_x_at | 7052 | TGM2 | 0,00031045 | 0,000394861 | 0,641704 | -0,640021 | -1,55835 |
| 222791_at | 54665 | RSBN1 | 0,00032396 | 0,000410149 | 0,463673 | -1,10882 | -2,15669 |
| 226252_at | --- | --- | 0,00032811 | 0,000413508 | 0,632424 | -0,661036 | -1,58122 |
| 235281_x_at | 79026 | AHNAK | 0,0003319 | 0,000416379 | 0,664089 | -0,590552 | -1,50582 |
| 224646_x_at | 283120 /// 100033819 | H19 /// MIR675 | 0,00033526 | 0,000418693 | 2,2299 | 1,15698 | 2,2299 |
| 224933_s_at | 221037 | JMJD1C | 0,00036338 | 0,000451765 | 0,603745 | -0,727988 | -1,65633 |
| 212240_s_at | 5295 | PIK3R1 | 0,00037287 | 0,000461425 | 0,391025 | -1,35467 | -2,55738 |
| 208151_x_at | 10521 | DDX17 | 0,0003747 | 0,000461425 | 0,663252 | -0,592372 | -1,50772 |
| 224997_x_at | 283120 /// 100033819 | H19 /// MIR675 | 0,00037616 | 0,000461425 | 1,56721 | 0,648194 | 1,56721 |
| 202971_s_at | 8445 | DYRK2 | 0,00038608 | 0,000471497 | 0,554778 | -0,850019 | -1,80252 |
| 223287_s_at | 27086 | FOXP1 | 0,00038944 | 0,000473504 | 0,595503 | -0,747818 | -1,67925 |
| 207186_s_at | 2186 | BPTF | 0,00039967 | 0,000483806 | 0,515038 | -0,957248 | -1,9416 |
| 219949_at | 79442 | LRRC2 | 0,00040507 | 0,000488205 | 1,82891 | 0,870981 | 1,82891 |
| 205547_s_at | 6876 | TAGLN | 0,00041549 | 0,000498592 | 0,626141 | -0,67544 | -1,59708 |
| 208791_at | 1191 | CLU | 0,00042311 | 0,000505535 | 0,548588 | -0,866206 | -1,82286 |
| 201019_s_at | 1964 /// 101060318 | EIF1AX /// LOC101060318 | 0,00042604 | 0,00050684 | 0,635977 | -0,652954 | -1,57238 |
| 208792_s_at | 1191 | CLU | 0,00043567 | 0,000515892 | 0,553374 | -0,853674 | -1,8071 |
| 212929_s_at | 55747 /// 253725 /// 387680 | FAM21A /// FAM21B /// FAM21C | 0,00043739 | 0,000515892 | 0,662763 | -0,593436 | -1,50884 |
| 202934_at | 3099 | HK2 | 0,00044135 | 0,000518355 | 1,55827 | 0,639946 | 1,55827 |
| 235538_at | --- | --- | 0,00045169 | 0,000526341 | 0,572819 | -0,803849 | -1,74575 |
| 221011_s_at | 81606 | LBH | 0,00045197 | 0,000526341 | 1,55742 | 0,639159 | 1,55742 |
| 229067_at | --- | --- | 0,00045616 | 0,000528992 | 0,574736 | -0,799028 | -1,73993 |
| 223189_x_at | 55904 | KMT2E | 0,00046005 | 0,000531276 | 0,482607 | -1,05108 | -2,07208 |
| 203698_s_at | 2487 | FRZB | 0,00048467 | 0,000557368 | 1,67284 | 0,742296 | 1,67284 |
| 212368_at | 23036 | ZNF292 | 0,00048734 | 0,000558114 | 0,527173 | -0,92365 | -1,89691 |
| 223139_s_at | 170506 | DHX36 | 0,00049736 | 0,00056724 | 0,452551 | -1,14385 | -2,20969 |
| 211992_at | 65125 | WNK1 | 0,00051426 | 0,000584102 | 0,654352 | -0,61186 | -1,52823 |
| 207983_s_at | 10735 | STAG2 | 0,00051647 | 0,000584198 | 0,548925 | -0,865318 | -1,82174 |
| 202404_s_at | 1278 | COL1A2 | 0,00052881 | 0,000595716 | 1,76099 | 0,816386 | 1,76099 |
| 227646_at | 1879 | EBF1 | 0,00055504 | 0,000622722 | 0,649287 | -0,623072 | -1,54015 |
| 229839_at | 286133 | SCARA5 | 0,00056394 | 0,000630154 | 0,631287 | -0,663632 | -1,58407 |
| 227121_at | --- | --- | 0,00056672 | 0,000630707 | 0,622379 | -0,684134 | -1,60674 |
| 205529_s_at | 862 | RUNX1T1 | 0,00059225 | 0,000656468 | 0,653653 | -0,613404 | -1,52986 |
| 212249_at | 5295 | PIK3R1 | 0,0005952 | 0,000657097 | 0,588845 | -0,764039 | -1,69824 |
| 228297_at | --- | --- | 0,00060066 | 0,000660486 | 0,406659 | -1,29811 | -2,45906 |
| 214911_s_at | 6046 | BRD2 | 0,00061241 | 0,000668808 | 0,643278 | -0,636485 | -1,55454 |
| 223519_at | 51776 | ZAK | 0,00061307 | 0,000668808 | 0,347536 | -1,52477 | -2,8774 |
| 229797_at | 55283 | MCOLN3 | 0,00062563 | 0,000679822 | 0,563113 | -0,828503 | -1,77584 |
| 205713_s_at | 1311 | COMP | 0,00064599 | 0,000699188 | 2,20704 | 1,14211 | 2,20704 |
| 233814_at | 1946 | EFNA5 | 0,00069199 | 0,000739862 | 0,634188 | -0,657018 | -1,57682 |
| 204359_at | 23768 /// 100506718 | FLRT2 /// LOC100506718 | 0,00069212 | 0,000739862 | 0,612379 | -0,707504 | -1,63298 |
| 219571_s_at | 7559 | ZNF12 | 0,00069337 | 0,000739862 | 0,581039 | -0,783294 | -1,72106 |
| 243584_at | --- | --- | 0,00069429 | 0,000739862 | 0,664126 | -0,59047 | -1,50574 |
| 209614_at | 125 | ADH1B | 0,00071137 | 0,000755151 | 0,665356 | -0,587802 | -1,50295 |
| 205168_at | 4921 | DDR2 | 0,00072102 | 0,000762454 | 0,564627 | -0,824631 | -1,77108 |
| 219789_at | 4883 | NPR3 | 0,00073731 | 0,000776705 | 0,537329 | -0,896122 | -1,86106 |
| 202600_s_at | 8204 | NRIP1 | 0,00076013 | 0,000797707 | 0,527076 | -0,923917 | -1,89726 |
| 219377_at | 64762 | GAREM | 0,00076834 | 0,000803266 | 0,635586 | -0,653841 | -1,57335 |
| 204326_x_at | 4501 | MT1X | 0,00077346 | 0,000805565 | 0,551198 | -0,859358 | -1,81423 |
| 229052_at | 51239 /// 200539 | ANKRD23 /// ANKRD39 | 0,00077995 | 0,000809268 | 0,576562 | -0,794452 | -1,73442 |
| 218930_s_at | 54664 | TMEM106B | 0,00078353 | 0,000809942 | 0,660747 | -0,59783 | -1,51344 |
| 228478_at | --- | --- | 0,00080916 | 0,000832463 | 0,450655 | -1,1499 | -2,21899 |
| 228740_at | --- | OTTHUMG00000172945 /// RP11-999E24.3 | 0,00081135 | 0,000832463 | 1,50615 | 0,590863 | 1,50615 |
| 230130_at | 9353 | SLIT2 | 0,00082639 | 0,000844757 | 0,648664 | -0,624456 | -1,54163 |
| 228545_at | 7707 | ZNF148 | 0,0008323 | 0,00084766 | 0,589159 | -0,763271 | -1,69734 |
| 212251_at | 92140 | MTDH | 0,00084319 | 0,000855592 | 0,621199 | -0,686874 | -1,60979 |
| 206555_s_at | 55623 | THUMPD1 | 0,00088794 | 0,000897693 | 0,617251 | -0,69607 | -1,62009 |
| 236034_at | 285 | ANGPT2 | 0,00089278 | 0,000899296 | 1,6046 | 0,682213 | 1,6046 |
| 215076_s_at | 1281 | COL3A1 | 0,00091048 | 0,000913793 | 1,50506 | 0,589821 | 1,50506 |
| 239849_at | --- | --- | 0,00097388 | 0,000973876 | 0,587007 | -0,768549 | -1,70356 |
