# Supplementary material for: Molecular Signatures of Human Chronic Atrial Fibrillation in Primary Mitral Regurgitation
Source: Cardiovasc Ther. 2021 Oct 15;2021:5516185. doi: 10.1155/2021/5516185 (PMC8538404; doi:10.1155/2021/5516185)
Supplement: Supplementary 6 — Supplementary Table 5: Venn diagrams of meta-analysis and comparisons of DEGs from different tissues. [file 5516185.f6.docx]

**Supplementary Table 5:** Venn diagrams of meta-analysis and comparisons of DEGs from different tissues.


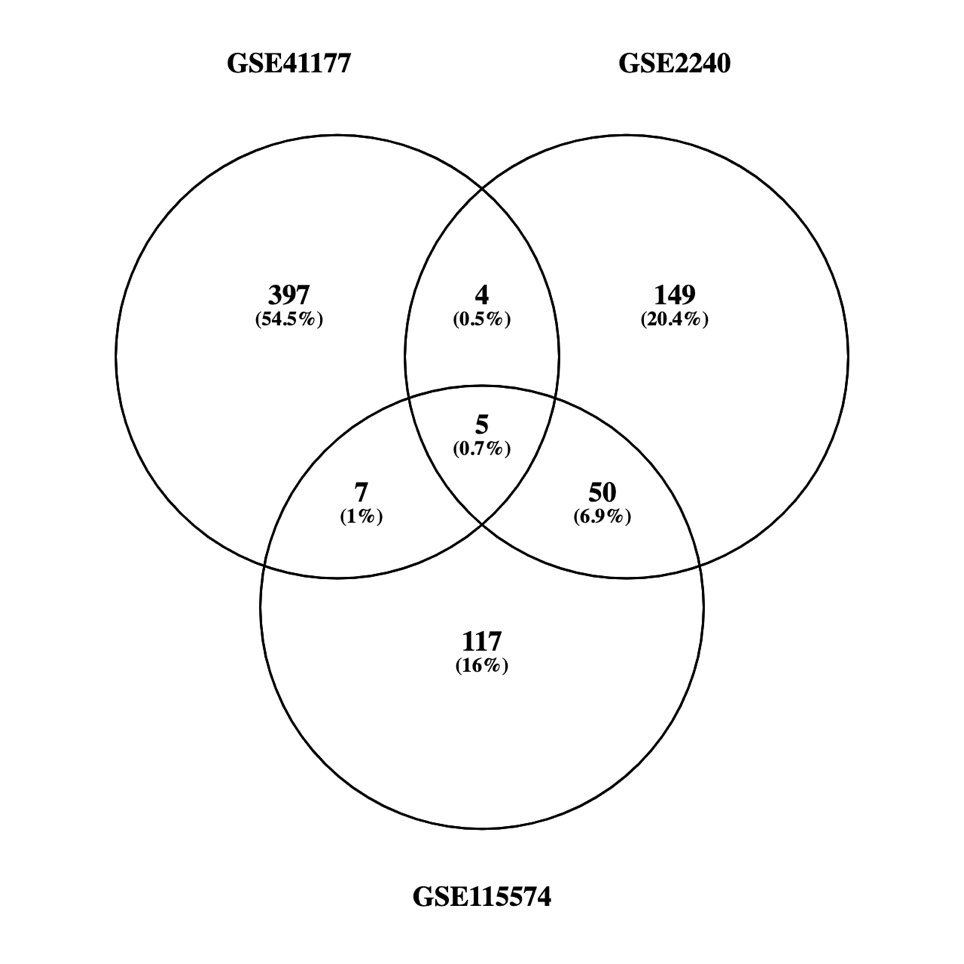


Venn diagram of differentially expressed genes of 2 study chosen for meta-analysis and our study. For qRT-PCR gene selection we used most up- or down-regulated in our data set and common genes in meta-analysis. All comparisons are AFib vs SR (Fold Change > 1.5; p<0.05)

common genes in venn diagram:

4: LEPR / LYVE1 / EPB41L2 / EDNRA

5: --- / DHRS9: 2.24 / RPL3L: 1.93 / ANKRD23 /// ANKRD39: -2.04 / ASTN2: -1.97

7: PSPH / COL4A6 / NUMA1 / CXCR4 / CCND2 / DPT / PPM1L

50: ATP1B4: 2.81 / COLQ: 2.63 / IGFBP2: 2.61 / COMP: 2.47 / SPP1: 2.1 / ANGPTL2: 2.09 / CHGB: 2.37 / DPYSL4: 1.90 / RCAN1: 1.86 / COL1A1: 1.85 / TNC: 1.84 / COL3A1: 1.71 / LBH: 1.70 / DNAJA4: 1.67 / H19 /// MIR675: 1.66 / COL1A2: 1.66 / CPLX1: 1.66 / HK2: 1.62 / TNNT1: 1.58 / VASH1: 1.54 / THY1: 1.53 / COL5A1: 1.52 / ETV5: 1.52 / SCARA5: -1.51 / GNAO1: -1.53 / CHL1: -1.55 / BMP7: -1.56 / NTRK2: -1.57 / FAM21A /// FAM21B /// FAM21C: -1.58 / SOSTDC1: -1.58 / OTTHUMG00000175814 /// RP11-13L2.4: -1.59 / NRXN1: -1.61 / MT1X: -1.62 / CPLX3: -1.65 / ART3: -1.68 / MYLK: -1.70 / LRRC49: -1.72 / GPR22: -1.78 / DNER: -1.80 / COG5: -1.81 / AQP4: -1.85 / LOC100653217 /// NTM: -1.86 / PPP1R1A: -1.88 / SFRP5: -1.92 / LGR6: -2 / SLC7A11: -2.12 / TRDN: -2.12 / LINC00844: -2.28 / TNNI1: -2.43 / MCOLN3: -2.81

(Genes: FC of GSE115574 study) Upregulated Downregulated Gene of interest

Venn diagram of differentially expressed genes from comparisons of AFib vs SR in tissues left atrium, right atrium, left+right atrium in GSE115574 study (our study) (Fold Change > 1.5; p<0.05)

**
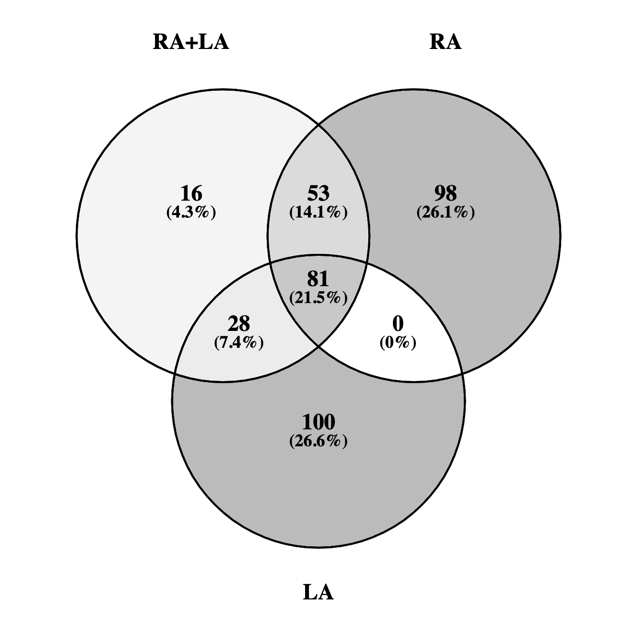
**

| **81 common elements in "RA+LA", "RA" and "LA"** | **28 common elements in "RA+LA" and "LA"** | **53 common elements in "RA+LA" and "RA"** |
| --- | --- | --- |
| MCOLN3  LOC100507477  CPLX3  TNNI1  AKAP3  PSPH  LINC00844  C1orf105  LRRN3  TRDN  SLC7A11  ANKRD23 /// ANKRD39  GALNT16  LGR6  STXBP6  ASTN2  CNTN3  OTOGL  SFRP5  BCHE  PPP1R1A  TLL2  FAM181B  AQP4  TRAC  COG5  CEL  GPR22  GADD45G  CACNB2  ENOSF1  ART3  ANKRD23  SUSD4  MUSTN1 /// TMEM110-MUSTN1  PPM1L  TMEM178A  SNX22  MPP3  RASGRP2  SOSTDC1  ULK4  BMP7  CHL1  COL4A6  GPNMB  ANGPTL2  RNF216  LINC00339  DKK3  NMNAT2  ATP13A3  EHD3  HK2  EXT1  CPLX1  DGKI  OTTHUMG00000015620 /// RP11-557H15.4  LBH  PDE8B  NES  LTBP2  SOGA2  USP46  KCNK3  TNC  RCAN1  DIRAS3  DPYSL4  RPL3L  DHRS9  OTTHUMG00000015496 /// RP11-532N4.2  LOC100128727  RELN  CHGB  SLC6A6  COMP  IGFBP2  COLQ  ATP1B4  NPPB | ATF3  NR4A2  LRRC49  SLN  MYBPC1  EGR1  MT1X  SLC27A6  NR4A1  KLF4  BEX2  KLF10  KDELR3  HTR2B  CYP26B1  GNG12  CCND2  SRPX2  LTBP1  THY1  PPARA  CYP1B1  SERPINE2  GSTT2  SFRP4  RASL11B  FHL2  SPP1 | ACTG2  LOC100653217 /// NTM  SYT13  RASD1  MYLK  LOC286087  CPNE5  HOPX  NRXN1  CACNA1G  SLC1A3  FAM21A /// FAM21B /// FAM21C  OTTHUMG00000175814 /// RP11-13L2.4  DLL1  ALCAM  NTRK2  RALGAPA2  GHRH  NRF1  CCDC163P  FAT3  GNAO1  DAPK2  RGS6  SCARA5  NUMA1  LOC100505729 /// VPS8  CXCR4  OTTHUMG00000019290 /// RP11-432J24.5  KIFAP3  PFKFB2  C4orf29  TMEM173  ETV5  PLCE1  CCND1  VASH1  HSPA2  CALU  TNNT1  ASPN  MARCH3  COL1A1  LAPTM4B  COL3A1  COL12A1  COL1A2  DNAJA4  RGS4  PRKAR1A  UNC5B-AS1  PHLDA1  MXRA5 |
