# Supplementary material for: Molecular Signatures of Human Chronic Atrial Fibrillation in Primary Mitral Regurgitation
Source: Cardiovasc Ther. 2021 Oct 15;2021:5516185. doi: 10.1155/2021/5516185 (PMC8538404; doi:10.1155/2021/5516185)
Supplement: Supplementary 7 — Supplementary Table 6: differentially expressed genes obtained from both LAA and RAA tissues of AFib vs. SR (fold change > 1.5; P < 0.05 and q < 0.05). [file 5516185.f7.docx]

**Supplementary Table 6:** Differentially expressed genes obtained from both LAA and RAA tissues of AFib vs. SR (Fold Change > 1.5; p<0.05 and q<0.05).

| **Probeset ID** | **Entrez Gene** | **Gene Symbol** | **p-value** | **q value** | **MeanRatio(AF/SR)** | **MeanDiff(AF-SR)** | **FoldChange(AF/SR)** |
| --- | --- | --- | --- | --- | --- | --- | --- |
| 213004_at | 23452 | ANGPTL2 | 6,18E-10 | 1,36509E-07 | 1,95536 | 0,967433 | 1,95536 |
| 204260_at | 1114 | CHGB | 1,62E-08 | 1,07134E-06 | 2,36891 | 1,24423 | 2,36891 |
| 1555869_a_at | 100507477 | LOC100507477 | 2,05E-08 | 1,07134E-06 | 0,393483 | -1,34563 | -2,5414 |
| 219514_at | 23452 | ANGPTL2 | 2,27E-08 | 1,07134E-06 | 1,52144 | 0,605439 | 1,52144 |
| 229797_at | 55283 | MCOLN3 | 2,42E-08 | 1,07134E-06 | 0,355531 | -1,49195 | -2,8127 |
| 222919_at | 10345 | TRDN | 6,09E-08 | 2,12892E-06 | 0,470372 | -1,08813 | -2,12598 |
| 213001_at | 23452 | ANGPTL2 | 6,74E-08 | 2,12892E-06 | 2,09373 | 1,06607 | 2,09373 |
| 214369_s_at | 10235 | RASGRP2 | 1,11E-07 | 2,87032E-06 | 0,622265 | -0,6844 | -1,60703 |
| 215843_s_at | 7093 | TLL2 | 1,17E-07 | 2,87032E-06 | 0,532638 | -0,908773 | -1,87745 |
| 231430_at | 220382 | FAM181B | 1,41E-07 | 3,11557E-06 | 0,534434 | -0,903915 | -1,87114 |
| 227065_at | 54476 | RNF216 | 3,04E-07 | 5,74469E-06 | 1,58808 | 0,667281 | 1,58808 |
| 243810_at | --- | --- | 3,12E-07 | 5,74469E-06 | 2,84772 | 1,50981 | 2,84772 |
| 230560_at | 29091 | STXBP6 | 3,72E-07 | 6,31781E-06 | 0,500324 | -0,999066 | -1,99871 |
| 218426_s_at | 54476 | RNF216 | 4,60E-07 | 6,86243E-06 | 1,53255 | 0,615932 | 1,53255 |
| 223952_x_at | 10170 | DHRS9 | 4,66E-07 | 6,86243E-06 | 2,17337 | 1,11994 | 2,17337 |
| 230418_s_at | 57452 | GALNT16 | 5,84E-07 | 7,79247E-06 | 0,49347 | -1,01897 | -2,02647 |
| 230384_at | 200539 | ANKRD23 | 5,99E-07 | 7,79247E-06 | 0,598533 | -0,740497 | -1,67075 |
| 1553746_a_at | 283310 | OTOGL | 6,42E-07 | 7,84613E-06 | 0,520876 | -0,940987 | -1,91984 |
| 225061_at | 55466 | DNAJA4 | 6,75E-07 | 7,84613E-06 | 1,75275 | 0,809622 | 1,75275 |
| 208370_s_at | 1827 | RCAN1 | 7,78E-07 | 8,60025E-06 | 1,86469 | 0,898936 | 1,86469 |
| 228754_at | 6533 | SLC6A6 | 1,18E-06 | 1,24698E-05 | 2,40456 | 1,26578 | 2,40456 |
| 210270_at | 9628 | RGS6 | 1,51E-06 | 1,5169E-05 | 0,656555 | -0,607012 | -1,5231 |
| 200604_s_at | 5573 | PRKAR1A | 1,60E-06 | 1,53755E-05 | 1,71751 | 0,780315 | 1,71751 |
| 224009_x_at | 10170 | DHRS9 | 1,72E-06 | 1,55668E-05 | 2,23922 | 1,16299 | 2,23922 |
| 207344_at | 10566 | AKAP3 | 1,76E-06 | 1,55668E-05 | 0,417483 | -1,26021 | -2,3953 |
| 240339_at | --- | --- | 2,34E-06 | 1,98697E-05 | 0,569414 | -0,812451 | -1,75619 |
| 201995_at | 2131 | EXT1 | 2,56E-06 | 2,01744E-05 | 1,65378 | 0,72577 | 1,65378 |
| 221288_at | 2845 | GPR22 | 2,56E-06 | 2,01744E-05 | 0,562144 | -0,830988 | -1,7789 |
| 209921_at | 23657 | SLC7A11 | 3,24E-06 | 2,47015E-05 | 0,471029 | -1,08611 | -2,12301 |
| 209841_s_at | 54674 | LRRN3 | 3,56E-06 | 2,62233E-05 | 0,459469 | -1,12196 | -2,17642 |
| 205952_at | 3777 | KCNK3 | 4,96E-06 | 3,34368E-05 | 1,79921 | 0,847363 | 1,79921 |
| 219799_s_at | 10170 | DHRS9 | 4,99E-06 | 3,34368E-05 | 1,93369 | 0,951353 | 1,93369 |
| 214357_at | 92346 | C1orf105 | 5,10E-06 | 3,34368E-05 | 0,45669 | -1,13071 | -2,18967 |
| 240395_at | 100128727 | LOC100128727 | 5,14E-06 | 3,34368E-05 | 2,12534 | 1,08769 | 2,12534 |
| 239882_at | --- | OTTHUMG00000015620 /// RP11-557H15.4 | 5,51E-06 | 3,47788E-05 | 1,69156 | 0,758358 | 1,69156 |
| 239474_at | --- | --- | 6,37E-06 | 3,81616E-05 | 1,90107 | 0,926809 | 1,90107 |
| 209590_at | 655 | BMP7 | 6,39E-06 | 3,81616E-05 | 0,638185 | -0,647954 | -1,56694 |
| 204763_s_at | 2775 | GNAO1 | 6,75E-06 | 3,92465E-05 | 0,653257 | -0,614277 | -1,53079 |
| 229302_at | 130733 | TMEM178A | 7,25E-06 | 4,11045E-05 | 0,606392 | -0,721677 | -1,6491 |
| 231048_at | --- | --- | 7,89E-06 | 4,29878E-05 | 0,569644 | -0,811868 | -1,75548 |
| 210380_s_at | 8913 | CACNA1G | 8,24E-06 | 4,29878E-05 | 0,628262 | -0,670562 | -1,59169 |
| 230577_at | 100507008 | LINC00844 | 8,26E-06 | 4,29878E-05 | 0,437592 | -1,19234 | -2,28523 |
| 1560164_at | --- | OTTHUMG00000015496 /// RP11-532N4.2 | 8,55E-06 | 4,29878E-05 | 2,04447 | 1,03173 | 2,04447 |
| 230417_at | 57452 | GALNT16 | 8,56E-06 | 4,29878E-05 | 0,561404 | -0,832889 | -1,78125 |
| 212929_s_at | 55747 /// 253725 /// 387680 | FAM21A /// FAM21B /// FAM21C | 8,84E-06 | 4,34134E-05 | 0,632004 | -0,661993 | -1,58227 |
| 206073_at | 8292 | COLQ | 1,15E-05 | 5,53403E-05 | 2,63803 | 1,39946 | 2,63803 |
| 209553_at | 23355 /// 100505729 | LOC100505729 /// VPS8 | 1,35E-05 | 6,20473E-05 | 1,50395 | 0,588757 | 1,50395 |
| 205478_at | 5502 | PPP1R1A | 1,36E-05 | 6,20473E-05 | 0,531128 | -0,912867 | -1,88278 |
| 223438_s_at | 5465 | PPARA | 1,38E-05 | 6,20473E-05 | 1,53324 | 0,616588 | 1,53324 |
| 219775_s_at | 594855 | CPLX3 | 1,46E-05 | 6,42837E-05 | 0,605729 | -0,723257 | -1,6509 |
| 207468_s_at | 6425 | SFRP5 | 1,48E-05 | 6,42837E-05 | 0,520905 | -0,940907 | -1,91974 |
| 227819_at | 59352 | LGR6 | 1,58E-05 | 6,65902E-05 | 0,497602 | -1,00694 | -2,00964 |
| 224916_at | 340061 | TMEM173 | 1,60E-05 | 6,65902E-05 | 1,52468 | 0,608503 | 1,52468 |
| 229831_at | 5067 | CNTN3 | 1,69E-05 | 6,93338E-05 | 0,518019 | -0,948923 | -1,93043 |
| 213456_at | 25928 | SOSTDC1 | 1,75E-05 | 7,05066E-05 | 0,631361 | -0,663462 | -1,58388 |
| 1557292_a_at | 55283 | MCOLN3 | 1,81E-05 | 7,10015E-05 | 0,536032 | -0,899608 | -1,86556 |
| 231040_at | --- | --- | 1,83E-05 | 7,10015E-05 | 0,646543 | -0,629181 | -1,54669 |
| 230509_at | 79856 | SNX22 | 1,91E-05 | 7,29144E-05 | 0,619712 | -0,690329 | -1,61365 |
| 243737_at | 23439 | ATP1B4 | 2,82E-05 | 0,000105489 | 2,81159 | 1,49138 | 2,81159 |
| 218425_at | 54476 | RNF216 | 3,07E-05 | 0,000113134 | 1,7062 | 0,770791 | 1,7062 |
| 1554789_a_at | 8622 | PDE8B | 3,24E-05 | 0,000117494 | 1,77225 | 0,82558 | 1,77225 |
| 203869_at | 64854 | USP46 | 3,70E-05 | 0,00013203 | 1,75965 | 0,815292 | 1,75965 |
| 222927_s_at | 594855 | CPLX3 | 4,15E-05 | 0,000145595 | 0,4112 | -1,28209 | -2,43191 |
| 228108_at | 151742 | PPM1L | 4,58E-05 | 0,000158278 | 0,606385 | -0,721695 | -1,64912 |
| 213256_at | 115123 | 3.Mar | 5,59E-05 | 0,000190147 | 1,59162 | 0,670499 | 1,59162 |
| 220994_s_at | 29091 | STXBP6 | 5,81E-05 | 0,000194562 | 0,603602 | -0,72833 | -1,65672 |
| 235759_at | --- | --- | 6,75E-05 | 0,000218302 | 0,657867 | -0,604133 | -1,52006 |
| 206806_at | 9162 | DGKI | 6,77E-05 | 0,000218302 | 1,65814 | 0,729564 | 1,65814 |
| 226228_at | 361 | AQP4 | 6,82E-05 | 0,000218302 | 0,539973 | -0,889042 | -1,85195 |
| 203629_s_at | 10466 | COG5 | 7,26E-05 | 0,000229321 | 0,606719 | -0,7209 | -1,64821 |
| 1554334_a_at | 55466 | DNAJA4 | 7,39E-05 | 0,000229959 | 1,66243 | 0,733297 | 1,66243 |
| 205713_s_at | 1311 | COMP | 7,52E-05 | 0,000230789 | 2,4752 | 1,30754 | 2,4752 |
| 230508_at | 27122 | DKK3 | 7,91E-05 | 0,000239598 | 1,55644 | 0,638252 | 1,55644 |
| 203630_s_at | 10466 | COG5 | 8,19E-05 | 0,000244537 | 0,55084 | -0,860296 | -1,81541 |
| 218935_at | 30845 | EHD3 | 8,31E-05 | 0,000244908 | 1,60705 | 0,684419 | 1,60705 |
| 205910_s_at | 1056 | CEL | 8,54E-05 | 0,000248258 | 0,550887 | -0,860171 | -1,81525 |
| 224215_s_at | 28514 | DLL1 | 8,93E-05 | 0,00025429 | 0,634779 | -0,655674 | -1,57535 |
| 219142_at | 65997 | RASL11B | 9,05E-05 | 0,00025429 | 1,61426 | 0,690871 | 1,61426 |
| 226856_at | 389125 /// 100526772 | MUSTN1 /// TMEM110-MUSTN1 | 9,20E-05 | 0,00025429 | 0,603585 | -0,72837 | -1,65677 |
| 215407_s_at | 23245 | ASTN2 | 9,21E-05 | 0,00025429 | 0,534603 | -0,903461 | -1,87055 |
| 219865_at | 29092 | LINC00339 | 0,000103374 | 0,000282045 | 1,53675 | 0,619883 | 1,53675 |
| 206768_at | 6123 | RPL3L | 0,000116608 | 0,000314273 | 1,93048 | 0,948958 | 1,93048 |
| 202800_at | 6507 | SLC1A3 | 0,000118794 | 0,000316307 | 0,630635 | -0,665123 | -1,5857 |
| 235129_at | 5502 | PPP1R1A | 0,00012067 | 0,000317477 | 0,562904 | -0,829039 | -1,7765 |
| 203940_s_at | 22846 | VASH1 | 0,000126595 | 0,000329147 | 1,54408 | 0,626751 | 1,54408 |
| 221796_at | 4915 | NTRK2 | 0,000132246 | 0,000337312 | 0,63835 | -0,64758 | -1,56654 |
| 217678_at | 23657 | SLC7A11 | 0,000132788 | 0,000337312 | 0,559472 | -0,837862 | -1,7874 |
| 221011_s_at | 81606 | LBH | 0,000141093 | 0,000351239 | 1,69328 | 0,759823 | 1,69328 |
| 205433_at | 590 | BCHE | 0,000141449 | 0,000351239 | 0,527059 | -0,923965 | -1,89732 |
| 209693_at | 23245 | ASTN2 | 0,000144847 | 0,00035568 | 0,507186 | -0,979414 | -1,97166 |
| 239136_at | 728978 | UNC5B-AS1 | 0,000159781 | 0,00038804 | 1,81259 | 0,858053 | 1,81259 |
| 1553970_s_at | 1056 | CEL | 0,000178397 | 0,000428541 | 0,555027 | -0,849371 | -1,80171 |
| 229052_at | 51239 /// 200539 | ANKRD23 /// ANKRD39 | 0,00018624 | 0,00044257 | 0,490336 | -1,02816 | -2,03942 |
| 202718_at | 3485 | IGFBP2 | 0,000207219 | 0,000487185 | 2,61422 | 1,38638 | 2,61422 |
| 213228_at | 8622 | PDE8B | 0,000212245 | 0,000493749 | 1,70051 | 0,765969 | 1,70051 |
| 1554018_at | 10457 | GPNMB | 0,000228416 | 0,000525833 | 1,51213 | 0,596579 | 1,51213 |
| 213645_at | 55556 | ENOSF1 | 0,000255114 | 0,000580605 | 0,58722 | -0,768028 | -1,70294 |
| 213358_at | 23255 | SOGA2 | 0,000257463 | 0,000580605 | 1,71037 | 0,774312 | 1,71037 |
| 205499_at | 27286 | SRPX2 | 0,000262085 | 0,000585058 | 1,52325 | 0,607154 | 1,52325 |
| 243189_at | 4899 | NRF1 | 0,000292436 | 0,000646284 | 0,643565 | -0,635842 | -1,55384 |
| 220484_at | 55283 | MCOLN3 | 0,000300234 | 0,000656948 | 0,589727 | -0,761881 | -1,6957 |
| 206186_at | 4356 | MPP3 | 0,000321699 | 0,000697015 | 0,62116 | -0,686962 | -1,60989 |
| 238780_s_at | --- | --- | 0,000370115 | 0,00079413 | 0,644105 | -0,634631 | -1,55254 |
| 202393_s_at | 7071 | KLF10 | 0,000381063 | 0,000809759 | 0,660246 | -0,598925 | -1,51459 |
| 205177_at | 7135 | TNNI1 | 0,000393775 | 0,000828803 | 0,411717 | -1,28028 | -2,42886 |
| 206801_at | 4879 | NPPB | 0,000416598 | 0,000865512 | 3,46553 | 1,79308 | 3,46553 |
| 242052_at | --- | --- | 0,000419049 | 0,000865512 | 1,50754 | 0,592193 | 1,50754 |
| 203333_at | 22920 | KIFAP3 | 0,000424847 | 0,000869363 | 1,51078 | 0,595289 | 1,51078 |
| 204017_at | 11015 | KDELR3 | 0,000456122 | 0,000924798 | 1,50464 | 0,589421 | 1,50464 |
| 236304_at | --- | --- | 0,000471605 | 0,000939889 | 2,13308 | 1,09294 | 2,13308 |
| 200755_s_at | 813 | CALU | 0,000472071 | 0,000939889 | 1,55787 | 0,639577 | 1,55787 |
| 236029_at | 120114 | FAT3 | 0,000490452 | 0,000967767 | 0,65048 | -0,620424 | -1,53733 |
| 204591_at | 10752 | CHL1 | 0,000541691 | 0,001059413 | 0,646987 | -0,62819 | -1,54562 |
| 219825_at | 56603 | CYP26B1 | 0,000547224 | 0,001060847 | 1,51049 | 0,595016 | 1,51049 |
| 203348_s_at | 2119 | ETV5 | 0,000573008 | 0,001101172 | 1,52562 | 0,609395 | 1,52562 |
| 215184_at | 23604 | DAPK2 | 0,000612023 | 0,001164128 | 0,653772 | -0,613141 | -1,52959 |
| 1559420_x_at | 783 | CACNB2 | 0,000616303 | 0,001164128 | 0,651468 | -0,618233 | -1,53499 |
| 205493_s_at | 10570 | DPYSL4 | 0,000622917 | 0,00116665 | 1,90292 | 0,928218 | 1,90292 |
| 204143_s_at | 55556 | ENOSF1 | 0,000630317 | 0,001170589 | 0,65874 | -0,602219 | -1,51805 |
| 203349_s_at | 2119 | ETV5 | 0,000650705 | 0,001197869 | 1,60802 | 0,685281 | 1,60802 |
| 204121_at | 10912 | GADD45G | 0,000655847 | 0,001197869 | 0,571364 | -0,807519 | -1,7502 |
| 1559419_at | 783 | CACNB2 | 0,000682893 | 0,001237044 | 0,586959 | -0,768669 | -1,7037 |
| 228547_at | 9378 | NRXN1 | 0,000747408 | 0,001342904 | 0,620527 | -0,688435 | -1,61153 |
| 214087_s_at | 4604 | MYBPC1 | 0,00077234 | 0,001376509 | 0,597931 | -0,741949 | -1,67243 |
| 223822_at | 55061 | SUSD4 | 0,000886532 | 0,001567389 | 0,600825 | -0,734983 | -1,66438 |
| 209670_at | 28755 | TRAC | 0,000923268 | 0,001618581 | 0,546986 | -0,870424 | -1,8282 |
| 241968_at | 80167 | C4orf29 | 0,000930135 | 0,001618581 | 1,5154 | 0,599698 | 1,5154 |
| 219389_at | 55061 | SUSD4 | 0,000953099 | 0,001645585 | 0,635183 | -0,654755 | -1,57435 |
| 219338_s_at | 54839 | LRRC49 | 0,000979692 | 0,001678387 | 0,580234 | -0,785294 | -1,72344 |
| 204682_at | 4053 | LTBP2 | 0,0010405 | 0,00176885 | 1,81186 | 0,857473 | 1,81186 |
| 234314_at | 57186 | RALGAPA2 | 0,00106309 | 0,001793457 | 0,641399 | -0,640707 | -1,55909 |
| 226086_at | 57586 | SYT13 | 0,0011935 | 0,001998208 | 0,573133 | -0,803059 | -1,7448 |
| 205112_at | 51196 | PLCE1 | 0,00128486 | 0,002134993 | 1,53079 | 0,614274 | 1,53079 |
| 206638_at | 3357 | HTR2B | 0,00131015 | 0,00216077 | 1,50765 | 0,592298 | 1,50765 |
| 1559003_a_at | 126661 | CCDC163P | 0,00140862 | 0,002305963 | 0,649282 | -0,623083 | -1,54016 |
| 232206_at | 54986 | ULK4 | 0,00146424 | 0,00237939 | 0,635942 | -0,653032 | -1,57247 |
| 209875_s_at | 6696 | SPP1 | 0,00150688 | 0,002430806 | 2,08282 | 1,05854 | 2,08282 |
| 205923_at | 5649 | RELN | 0,00153308 | 0,00245515 | 2,19247 | 1,13256 | 2,19247 |
| 224367_at | 84707 | BEX2 | 0,00164564 | 0,002616449 | 0,658114 | -0,60359 | -1,51949 |
| 224823_at | 4638 | MYLK | 0,00178302 | 0,002814624 | 0,589375 | -0,762741 | -1,69671 |
| 209596_at | 25878 | MXRA5 | 0,00184489 | 0,002891636 | 2,08459 | 1,05976 | 2,08459 |
| 223500_at | 10815 | CPLX1 | 0,00192148 | 0,002979899 | 1,65702 | 0,728595 | 1,65702 |
| 201645_at | 3371 | TNC | 0,00192817 | 0,002979899 | 1,84068 | 0,880236 | 1,84068 |
| 227189_at | 57699 | CPNE5 | 0,00210679 | 0,003233337 | 0,602169 | -0,73176 | -1,66066 |
| 208850_s_at | 7070 | THY1 | 0,00218372 | 0,00332829 | 1,5315 | 0,614943 | 1,5315 |
| 210147_at | 419 | ART3 | 0,00220361 | 0,003335601 | 0,593678 | -0,752247 | -1,68441 |
| 205048_s_at | 5723 | PSPH | 0,00225223 | 0,003371639 | 0,424136 | -1,2374 | -2,35774 |
| 202934_at | 3099 | HK2 | 0,00225793 | 0,003371639 | 1,61182 | 0,688687 | 1,61182 |
| 202672_s_at | 467 | ATF3 | 0,00251473 | 0,003729902 | 0,43064 | -1,21545 | -2,32212 |
| 215506_s_at | 9077 | DIRAS3 | 0,00253588 | 0,003736197 | 1,89698 | 0,923703 | 1,89698 |
| 229580_at | --- | OTTHUMG00000175814 /// RP11-13L2.4 | 0,00281535 | 0,004100001 | 0,632668 | -0,66048 | -1,58061 |
| 1557149_at | --- | OTTHUMG00000019290 /// RP11-432J24.5 | 0,00281991 | 0,004100001 | 1,50975 | 0,59431 | 1,50975 |
| 205439_at | 2953 | GSTT2 | 0,00300259 | 0,004337074 | 1,57608 | 0,656338 | 1,57608 |
| 1556842_at | 286087 | LOC286087 | 0,00313826 | 0,004503607 | 0,598657 | -0,740198 | -1,67041 |
| 204326_x_at | 4501 | MT1X | 0,00345324 | 0,004923652 | 0,61754 | -0,695395 | -1,61933 |
| 225842_at | 22822 | PHLDA1 | 0,00355324 | 0,005033757 | 2,05422 | 1,03859 | 2,05422 |
| 226733_at | 5208 | PFKFB2 | 0,00361713 | 0,005069027 | 1,51377 | 0,59815 | 1,51377 |
| 225664_at | 1303 | COL12A1 | 0,00362401 | 0,005069027 | 1,62707 | 0,702274 | 1,62707 |
| 204622_x_at | 4929 | NR4A2 | 0,00372639 | 0,005179448 | 0,479792 | -1,05952 | -2,08424 |
| 204051_s_at | 6424 | SFRP4 | 0,00377255 | 0,005210835 | 1,60592 | 0,683399 | 1,60592 |
| 1554663_a_at | 4926 | NUMA1 | 0,00384479 | 0,005277631 | 0,661519 | -0,596146 | -1,51167 |
| 209755_at | 23057 | NMNAT2 | 0,00404202 | 0,005514114 | 1,56842 | 0,649315 | 1,56842 |
| 217997_at | 22822 | PHLDA1 | 0,00406737 | 0,005514655 | 2,1097 | 1,07704 | 2,1097 |
| 209840_s_at | 54674 | LRRN3 | 0,00480501 | 0,006475044 | 0,602199 | -0,731689 | -1,66058 |
| 223690_at | 4053 | LTBP2 | 0,00490532 | 0,006559653 | 1,70698 | 0,771444 | 1,70698 |
| 202310_s_at | 1277 | COL1A1 | 0,00492716 | 0,006559653 | 1,85558 | 0,891868 | 1,85558 |
| 213992_at | 1288 | COL4A6 | 0,0050679 | 0,006706622 | 0,661707 | -0,595735 | -1,51124 |
| 216248_s_at | 4929 | NR4A2 | 0,00512307 | 0,006739277 | 0,518077 | -0,948761 | -1,93021 |
| 214524_at | 2691 | GHRH | 0,00517144 | 0,006762652 | 0,64153 | -0,640412 | -1,55877 |
| 222834_s_at | 55970 | GNG12 | 0,00542968 | 0,007058584 | 1,51294 | 0,597351 | 1,51294 |
| 219087_at | 54829 | ASPN | 0,00551273 | 0,007124639 | 1,58399 | 0,663562 | 1,58399 |
| 229346_at | 10763 | NES | 0,00593808 | 0,007629742 | 1,70317 | 0,768223 | 1,70317 |
| 211538_s_at | 3306 | HSPA2 | 0,00653911 | 0,00835343 | 1,5552 | 0,637103 | 1,5552 |
| 221730_at | 1290 | COL5A2 | 0,00679467 | 0,008630012 | 1,54834 | 0,630726 | 1,54834 |
| 203570_at | 4016 | LOXL1 | 0,00737649 | 0,009315453 | 1,65232 | 0,724492 | 1,65232 |
| 201387_s_at | 7345 | UCHL1 | 0,00784101 | 0,009845814 | 1,53517 | 0,618403 | 1,53517 |
| 223467_at | 51655 | RASD1 | 0,00836744 | 0,010408293 | 0,583294 | -0,777705 | -1,7144 |
| 202728_s_at | 4052 | LTBP1 | 0,00838315 | 0,010408293 | 1,52463 | 0,608457 | 1,52463 |
| 202340_x_at | 3164 | NR4A1 | 0,00864144 | 0,01066904 | 0,637276 | -0,65001 | -1,56918 |
| 213201_s_at | 7138 | TNNT1 | 0,00872175 | 0,010708371 | 1,57866 | 0,658705 | 1,57866 |
| 1564378_a_at | --- | --- | 0,00889633 | 0,01086237 | 1,55695 | 0,638726 | 1,55695 |
| 204621_s_at | 4929 | NR4A2 | 0,00911109 | 0,011063466 | 0,563913 | -0,826456 | -1,77332 |
| 204337_at | 5999 | RGS4 | 0,009266 | 0,011190087 | 1,67726 | 0,746108 | 1,67726 |
| 205374_at | 6588 | SLN | 0,00945688 | 0,011358535 | 0,590154 | -0,760837 | -1,69447 |
| 219558_at | 79572 | ATP13A3 | 0,00965146 | 0,011529582 | 1,60447 | 0,682096 | 1,60447 |
| 219932_at | 28965 | SLC27A6 | 0,0100913 | 0,011990201 | 0,63545 | -0,65415 | -1,57369 |
| 212488_at | 1289 | COL5A1 | 0,0103097 | 0,012184191 | 1,51806 | 0,602233 | 1,51806 |
| 212190_at | 5270 | SERPINE2 | 0,0104934 | 0,012319354 | 1,56689 | 0,647904 | 1,56689 |
| 201236_s_at | 7832 | BTG2 | 0,0105364 | 0,012319354 | 0,642135 | -0,639051 | -1,5573 |
| 202274_at | 72 | ACTG2 | 0,0105913 | 0,012319354 | 0,525181 | -0,929114 | -1,90411 |
| 217996_at | 22822 | PHLDA1 | 0,0110003 | 0,012728096 | 2,09508 | 1,06701 | 2,09508 |
| 217028_at | 7852 | CXCR4 | 0,0110979 | 0,012774145 | 1,50876 | 0,593367 | 1,50876 |
| 227566_at | 50863 /// 100653217 | LOC100653217 /// NTM | 0,0117773 | 0,013485924 | 0,536378 | -0,898678 | -1,86436 |
| 1556499_s_at | 1277 | COL1A1 | 0,0122444 | 0,013948518 | 1,59852 | 0,676741 | 1,59852 |
| 202436_s_at | 1545 | CYP1B1 | 0,0129604 | 0,014688453 | 1,5462 | 0,628723 | 1,5462 |
| 211597_s_at | 84525 | HOPX | 0,0135325 | 0,015258584 | 0,609756 | -0,713696 | -1,64 |
| 228450_at | 144100 | PLEKHA7 | 0,0138356 | 0,015521155 | 1,57382 | 0,654267 | 1,57382 |
| 200951_s_at | 894 | CCND2 | 0,0152985 | 0,017075598 | 1,51778 | 0,601964 | 1,51778 |
| 240065_at | 153643 | FAM81B | 0,0155821 | 0,017304744 | 0,615 | -0,701341 | -1,62602 |
| 1560169_at | --- | --- | 0,0173182 | 0,019136611 | 1,62899 | 0,703982 | 1,62899 |
| 226281_at | 92737 | DNER | 0,0182592 | 0,020076036 | 0,557904 | -0,84191 | -1,79242 |
| 213975_s_at | 4069 | LYZ | 0,0195449 | 0,021383282 | 1,55851 | 0,640171 | 1,55851 |
| 201852_x_at | 1281 | COL3A1 | 0,0196636 | 0,02140717 | 1,71986 | 0,782291 | 1,71986 |
| 201952_at | 214 | ALCAM | 0,0198208 | 0,021472533 | 0,63569 | -0,653605 | -1,57309 |
| 207977_s_at | 1805 | DPT | 0,021108 | 0,022755454 | 1,5725 | 0,653058 | 1,5725 |
| 215076_s_at | 1281 | COL3A1 | 0,0214182 | 0,022977778 | 1,61406 | 0,690692 | 1,61406 |
| 208711_s_at | 595 | CCND1 | 0,0223076 | 0,023816327 | 1,54337 | 0,626086 | 1,54337 |
| 227140_at | 3624 | INHBA | 0,0227386 | 0,024159763 | 0,610277 | -0,712465 | -1,6386 |
| 1554679_a_at | 55353 | LAPTM4B | 0,0236482 | 0,025005991 | 1,60704 | 0,68441 | 1,60704 |
| 212922_s_at | 56950 | SMYD2 | 0,0245184 | 0,025802697 | 1,56097 | 0,642439 | 1,56097 |
| 233276_at | --- | --- | 0,0256954 | 0,026913191 | 0,647064 | -0,628019 | -1,54544 |
| 202404_s_at | 1278 | COL1A2 | 0,0261892 | 0,027301006 | 1,65745 | 0,728963 | 1,65745 |
| 229839_at | 286133 | SCARA5 | 0,0313888 | 0,032567722 | 0,661036 | -0,5972 | -1,51278 |
| 222020_s_at | 50863 /// 100653217 | LOC100653217 /// NTM | 0,0331889 | 0,034274518 | 0,603983 | -0,727419 | -1,65567 |
| 203815_at | 2952 | GSTT1 | 0,0353139 | 0,036299404 | 0,512145 | -0,965377 | -1,95257 |
| 201694_s_at | 1958 | EGR1 | 0,0385166 | 0,039408188 | 0,607064 | -0,720079 | -1,64727 |
| 221841_s_at | 9314 | KLF4 | 0,0395608 | 0,040290031 | 0,646339 | -0,629637 | -1,54718 |
| 1557146_a_at | 146336 | SSTR5-AS1 | 0,0404696 | 0,041026521 | 0,592056 | -0,756194 | -1,68903 |
| 202949_s_at | 2274 | FHL2 | 0,0410277 | 0,041402382 | 1,62355 | 0,699148 | 1,62355 |
| 210764_s_at | 3491 | CYR61 | 0,0423367 | 0,04252914 | 0,630234 | -0,666041 | -1,58671 |
| 224646_x_at | 283120 /// 100033819 | H19 /// MIR675 | 0,0480695 | 0,0480695 | 1,66309 | 0,733868 | 1,66309 |
