# Supplementary material for: Molecular Signatures of Human Chronic Atrial Fibrillation in Primary Mitral Regurgitation
Source: Cardiovasc Ther. 2021 Oct 15;2021:5516185. doi: 10.1155/2021/5516185 (PMC8538404; doi:10.1155/2021/5516185)
Supplement: Supplementary 8 — Supplementary Table 7: differentially expressed genes in RAA tissues of AFib vs. SR (fold change > 1.5; P < 0.05 and q < 0.05). [file 5516185.f8.docx]

**Supplementary Table 7:** Differentially expressed genes in RAA tissues of AFib vs SR (Fold Change > 1.5; p<0.05 and q<0.05).

| **Probeset ID** | **Entrez Gene** | **Gene Symbol** | **p-value** | **q value** | **MeanRatio(AF/SR)** | **MeanDiff(AF-SR)** | **FoldChange(AF/SR)** |
| --- | --- | --- | --- | --- | --- | --- | --- |
| 225061_at | 55466 | DNAJA4 | 3,40E-07 | 9,9531E-05 | 2,08694 | 1,06139 | 2,08694 |
| 200604_s_at | 5573 | PRKAR1A | 7,54E-07 | 9,9531E-05 | 2,00818 | 1,00589 | 2,00818 |
| 221796_at | 4915 | NTRK2 | 9,54E-07 | 9,9531E-05 | 0,48714 | -1,03759 | -2,0528 |
| 218426_s_at | 54476 | RNF216 | 2,21E-06 | 0,00017287 | 1,73186 | 0,792325 | 1,73186 |
| 213004_at | 23452 | ANGPTL2 | 3,20E-06 | 0,00019996 | 1,91217 | 0,93521 | 1,91217 |
| 213001_at | 23452 | ANGPTL2 | 3,83E-06 | 0,00019996 | 2,07158 | 1,05073 | 2,07158 |
| 231430_at | 220382 | FAM181B | 6,32E-06 | 0,00028275 | 0,473997 | -1,07705 | -2,10972 |
| 205553_s_at | 8048 | CSRP3 | 7,87E-06 | 0,00030514 | 1,50594 | 0,590662 | 1,50594 |
| 236029_at | 120114 | FAT3 | 8,77E-06 | 0,00030514 | 0,506091 | -0,98253 | -1,97593 |
| 236726_at | 9628 | RGS6 | 1,31E-05 | 0,00041106 | 0,619243 | -0,691422 | -1,61487 |
| 200757_s_at | 813 | CALU | 1,68E-05 | 0,00045029 | 1,53224 | 0,615642 | 1,53224 |
| 223952_x_at | 10170 | DHRS9 | 1,73E-05 | 0,00045029 | 2,38908 | 1,25646 | 2,38908 |
| 204260_at | 1114 | CHGB | 2,51E-05 | 0,00056622 | 2,34426 | 1,22913 | 2,34426 |
| 219514_at | 23452 | ANGPTL2 | 2,53E-05 | 0,00056622 | 1,5426 | 0,625366 | 1,5426 |
| 210270_at | 9628 | RGS6 | 3,79E-05 | 0,00076568 | 0,602172 | -0,731752 | -1,66065 |
| 208370_s_at | 1827 | RCAN1 | 3,93E-05 | 0,00076568 | 2,0544 | 1,03872 | 2,0544 |
| 203333_at | 22920 | KIFAP3 | 4,19E-05 | 0,00076568 | 1,68778 | 0,755128 | 1,68778 |
| 209921_at | 23657 | SLC7A11 | 4,40E-05 | 0,00076568 | 0,417849 | -1,25894 | -2,39321 |
| 229797_at | 55283 | MCOLN3 | 4,71E-05 | 0,00077073 | 0,411948 | -1,27947 | -2,42749 |
| 224009_x_at | 10170 | DHRS9 | 5,29E-05 | 0,00077073 | 2,44365 | 1,28904 | 2,44365 |
| 228754_at | 6533 | SLC6A6 | 5,48E-05 | 0,00077073 | 2,86263 | 1,51734 | 2,86263 |
| 222919_at | 10345 | TRDN | 5,62E-05 | 0,00077073 | 0,52259 | -0,936248 | -1,91355 |
| 219775_s_at | 594855 | CPLX3 | 5,66E-05 | 0,00077073 | 0,558154 | -0,841264 | -1,79162 |
| 228376_at | 2681 | GGTA1P | 6,92E-05 | 0,00090196 | 0,618375 | -0,693446 | -1,61714 |
| 219799_s_at | 10170 | DHRS9 | 7,48E-05 | 0,00093626 | 2,16186 | 1,11227 | 2,16186 |
| 206073_at | 8292 | COLQ | 8,75E-05 | 0,00105283 | 3,27313 | 1,71067 | 3,27313 |
| 221288_at | 2845 | GPR22 | 9,17E-05 | 0,00106353 | 0,533094 | -0,907539 | -1,87584 |
| 1554334_a_at | 55466 | DNAJA4 | 0,00010442 | 0,00114243 | 1,95981 | 0,970712 | 1,95981 |
| 1552721_a_at | 2246 | FGF1 | 0,00010585 | 0,00114243 | 1,50898 | 0,593571 | 1,50898 |
| 224215_s_at | 28514 | DLL1 | 0,00011337 | 0,00118286 | 0,529776 | -0,916545 | -1,88759 |
| 243810_at | --- | --- | 0,0001387 | 0,00138064 | 3,06472 | 1,61575 | 3,06472 |
| 1557292_a_at | 55283 | MCOLN3 | 0,00014115 | 0,00138064 | 0,507095 | -0,979672 | -1,97202 |
| 231024_at | 572558 | PGM5-AS1 | 0,00016151 | 0,00144025 | 0,665655 | -0,587154 | -1,50228 |
| 239921_at | 340267 | COL28A1 | 0,00016221 | 0,00144025 | 0,580404 | -0,78487 | -1,72294 |
| 208712_at | 595 | CCND1 | 0,00016445 | 0,00144025 | 1,52438 | 0,608223 | 1,52438 |
| 241948_at | --- | --- | 0,00016565 | 0,00144025 | 1,56703 | 0,648036 | 1,56703 |
| 202780_at | 5019 | OXCT1 | 0,00018484 | 0,00156364 | 1,72946 | 0,790324 | 1,72946 |
| 214369_s_at | 10235 | RASGRP2 | 0,00019758 | 0,00162742 | 0,607337 | -0,719431 | -1,64653 |
| 239136_at | 728978 | UNC5B-AS1 | 0,00021616 | 0,00173484 | 2,17119 | 1,11849 | 2,17119 |
| 202196_s_at | 27122 | DKK3 | 0,00024189 | 0,00184749 | 1,58954 | 0,668606 | 1,58954 |
| 227065_at | 54476 | RNF216 | 0,00025691 | 0,00184749 | 1,63573 | 0,709937 | 1,63573 |
| 203827_at | 55062 | WIPI1 | 0,00026665 | 0,00184749 | 1,5483 | 0,630682 | 1,5483 |
| 204157_s_at | 23387 | SIK3 | 0,00026744 | 0,00184749 | 1,50653 | 0,591225 | 1,50653 |
| 204763_s_at | 2775 | GNAO1 | 0,00027247 | 0,00184749 | 0,642528 | -0,638168 | -1,55635 |
| 201848_s_at | 664 | BNIP3 | 0,00027784 | 0,00184749 | 1,59758 | 0,675888 | 1,59758 |
| 230408_at | --- | --- | 0,00028029 | 0,00184749 | 0,627844 | -0,671523 | -1,59275 |
| 1554018_at | 10457 | GPNMB | 0,0002829 | 0,00184749 | 1,54153 | 0,624359 | 1,54153 |
| 1569607_s_at | 84210 /// 284232 /// 391267 /// 440482 /// 441425 /// 441430 /// 728747 /// 101059935 | ANKRD20A1 /// ANKRD20A11P /// ANKRD20A2 /// ANKRD20A3 /// ANKRD20A4 /// ANKRD20A5P /// ANKRD20A9P /// LOC101059935 | 0,00028332 | 0,00184749 | 0,539281 | -0,890891 | -1,85432 |
| 203869_at | 64854 | USP46 | 0,00030771 | 0,0019536 | 1,85205 | 0,889124 | 1,85205 |
| 227819_at | 59352 | LGR6 | 0,00031208 | 0,0019536 | 0,441805 | -1,17852 | -2,26344 |
| 240339_at | --- | --- | 0,00033343 | 0,00202385 | 0,534202 | -0,904543 | -1,87195 |
| 200838_at | 1508 | CTSB | 0,00033623 | 0,00202385 | 1,65157 | 0,723842 | 1,65157 |
| 228329_at | 1600 | DAB1 | 0,00034597 | 0,00202947 | 0,646322 | -0,629674 | -1,54722 |
| 230560_at | 29091 | STXBP6 | 0,00035013 | 0,00202947 | 0,492147 | -1,02284 | -2,03191 |
| 206768_at | 6123 | RPL3L | 0,00037393 | 0,00212799 | 2,24011 | 1,16357 | 2,24011 |
| 1555869_a_at | 100507477 | LOC100507477 | 0,00043784 | 0,00240866 | 0,422623 | -1,24256 | -2,36617 |
| 205952_at | 3777 | KCNK3 | 0,00043864 | 0,00240866 | 1,89077 | 0,918973 | 1,89077 |
| 217678_at | 23657 | SLC7A11 | 0,00046452 | 0,00250678 | 0,49169 | -1,02418 | -2,0338 |
| 217999_s_at | 22822 | PHLDA1 | 0,00049152 | 0,00257962 | 1,55811 | 0,639796 | 1,55811 |
| 242600_at | 257019 | FRMD3 | 0,0004945 | 0,00257962 | 1,61671 | 0,693065 | 1,61671 |
| 204754_at | 3131 | HLF | 0,00061057 | 0,00308502 | 0,592706 | -0,754612 | -1,68718 |
| 205392_s_at | 6358 /// 6359 /// 348249 | CCL14 /// CCL15 /// CCL15-CCL14 | 0,00061423 | 0,00308502 | 0,558932 | -0,839255 | -1,78913 |
| 229893_at | 257019 | FRMD3 | 0,00062095 | 0,00308502 | 1,62395 | 0,699505 | 1,62395 |
| 239882_at | --- | OTTHUMG00000015620 /// RP11-557H15.4 | 0,00063101 | 0,00308605 | 1,73751 | 0,79702 | 1,73751 |
| 217996_at | 22822 | PHLDA1 | 0,00068183 | 0,00325156 | 3,43394 | 1,77987 | 3,43394 |
| 226733_at | 5208 | PFKFB2 | 0,00071576 | 0,00325156 | 1,73242 | 0,792788 | 1,73242 |
| 243737_at | 23439 | ATP1B4 | 0,00072461 | 0,00325156 | 2,9046 | 1,53834 | 2,9046 |
| 229831_at | 5067 | CNTN3 | 0,00072692 | 0,00325156 | 0,532879 | -0,908121 | -1,8766 |
| 226435_at | 89932 | PAPLN | 0,00072709 | 0,00325156 | 0,558389 | -0,840657 | -1,79086 |
| 205733_at | 641 | BLM | 0,00072719 | 0,00325156 | 0,576993 | -0,793374 | -1,73312 |
| 209590_at | 655 | BMP7 | 0,00075148 | 0,00330681 | 0,633309 | -0,659018 | -1,57901 |
| 205117_at | 2246 | FGF1 | 0,00076067 | 0,00330681 | 1,59555 | 0,674058 | 1,59555 |
| 203940_s_at | 22846 | VASH1 | 0,0008052 | 0,00345245 | 1,62342 | 0,699037 | 1,62342 |
| 210380_s_at | 8913 | CACNA1G | 0,0008272 | 0,00346041 | 0,583004 | -0,778423 | -1,71526 |
| 213725_x_at | 64131 | XYLT1 | 0,00082917 | 0,00346041 | 1,58439 | 0,663926 | 1,58439 |
| 217997_at | 22822 | PHLDA1 | 0,00088174 | 0,00358801 | 3,12389 | 1,64334 | 3,12389 |
| 212929_s_at | 55747 /// 253725 /// 387680 | FAM21A /// FAM21B /// FAM21C | 0,00090302 | 0,00358801 | 0,599259 | -0,738749 | -1,66873 |
| 212297_at | 79572 | ATP13A3 | 0,00090488 | 0,00358801 | 1,57168 | 0,652304 | 1,57168 |
| 215768_at | --- | --- | 0,0009132 | 0,00358801 | 0,577897 | -0,791117 | -1,73041 |
| 207468_s_at | 6425 | SFRP5 | 0,0009212 | 0,00358801 | 0,460135 | -1,11987 | -2,17327 |
| 209841_s_at | 54674 | LRRN3 | 0,00092853 | 0,00358801 | 0,475382 | -1,07284 | -2,10357 |
| 239474_at | --- | --- | 0,00097901 | 0,00370313 | 2,03125 | 1,02237 | 2,03125 |
| 227662_at | 171024 | SYNPO2 | 0,00098198 | 0,00370313 | 0,649375 | -0,622876 | -1,53994 |
| 213355_at | 10402 | ST3GAL6 | 0,00101566 | 0,00378454 | 1,51663 | 0,600868 | 1,51663 |
| 227899_at | 5212 | VIT | 0,00104112 | 0,00381281 | 0,586506 | -0,769783 | -1,70501 |
| 203870_at | 64854 | USP46 | 0,00104761 | 0,00381281 | 1,50755 | 0,592203 | 1,50755 |
| 230418_s_at | 57452 | GALNT16 | 0,0010918 | 0,00390606 | 0,491455 | -1,02487 | -2,03477 |
| 211015_s_at | 3308 | HSPA4 | 0,00109819 | 0,00390606 | 1,51544 | 0,599733 | 1,51544 |
| 227917_at | 100506990 | LOC100506990 | 0,00115088 | 0,00403631 | 0,643455 | -0,636089 | -1,55411 |
| 238780_s_at | --- | --- | 0,00117875 | 0,00403631 | 0,575641 | -0,796758 | -1,73719 |
| 228547_at | 9378 | NRXN1 | 0,00118136 | 0,00403631 | 0,574578 | -0,799425 | -1,74041 |
| 203140_at | 604 | BCL6 | 0,00118639 | 0,00403631 | 0,659369 | -0,600843 | -1,5166 |
| 224916_at | 340061 | TMEM173 | 0,00120781 | 0,00406499 | 1,52519 | 0,608988 | 1,52519 |
| 229839_at | 286133 | SCARA5 | 0,00122243 | 0,00407043 | 0,468976 | -1,09241 | -2,13231 |
| 227566_at | 50863 /// 100653217 | LOC100653217 /// NTM | 0,00124244 | 0,00409351 | 0,52215 | -0,937463 | -1,91516 |
| 201995_at | 2131 | EXT1 | 0,00129456 | 0,00417864 | 1,60672 | 0,684117 | 1,60672 |
| 203630_s_at | 10466 | COG5 | 0,00130774 | 0,00417864 | 0,531258 | -0,912515 | -1,88232 |
| 210280_at | 4359 | MPZ | 0,00131773 | 0,00417864 | 0,657375 | -0,605211 | -1,5212 |
| 202934_at | 3099 | HK2 | 0,00132168 | 0,00417864 | 1,81004 | 0,856022 | 1,81004 |
| 213256_at | 115123 | 3.Mar | 0,00133758 | 0,00418663 | 1,68204 | 0,750209 | 1,68204 |
| 215407_s_at | 23245 | ASTN2 | 0,00137504 | 0,00426126 | 0,460805 | -1,11777 | -2,17011 |
| 227845_s_at | 56961 | SHD | 0,00139679 | 0,00428623 | 0,592108 | -0,756067 | -1,68888 |
| 229302_at | 130733 | TMEM178A | 0,00148366 | 0,0044836 | 0,609262 | -0,714865 | -1,64133 |
| 200755_s_at | 813 | CALU | 0,00148976 | 0,0044836 | 1,65831 | 0,729712 | 1,65831 |
| 205112_at | 51196 | PLCE1 | 0,00151732 | 0,00452306 | 1,70221 | 0,767413 | 1,70221 |
| 215843_s_at | 7093 | TLL2 | 0,00154363 | 0,00455808 | 0,600111 | -0,736699 | -1,66636 |
| 209693_at | 23245 | ASTN2 | 0,00157534 | 0,00460824 | 0,448672 | -1,15627 | -2,2288 |
| 203349_s_at | 2119 | ETV5 | 0,0016253 | 0,00471036 | 1,79877 | 0,847012 | 1,79877 |
| 216481_at | 80852 | GRIP2 | 0,0016715 | 0,00479981 | 1,5895 | 0,668571 | 1,5895 |
| 202800_at | 6507 | SLC1A3 | 0,0016966 | 0,00480729 | 0,552615 | -0,855654 | -1,80958 |
| 209164_s_at | 1534 | CYB561 | 0,00171655 | 0,00480729 | 0,620744 | -0,68793 | -1,61097 |
| 241925_x_at | 9194 | SLC16A7 | 0,00172018 | 0,00480729 | 1,60357 | 0,681291 | 1,60357 |
| 221011_s_at | 81606 | LBH | 0,00176375 | 0,00488543 | 1,63773 | 0,711698 | 1,63773 |
| 219147_s_at | 54981 | NMRK1 | 0,00179167 | 0,0049006 | 0,661204 | -0,596833 | -1,51239 |
| 226228_at | 361 | AQP4 | 0,00180054 | 0,0049006 | 0,465903 | -1,1019 | -2,14637 |
| 222927_s_at | 594855 | CPLX3 | 0,00186654 | 0,00503644 | 0,339648 | -1,55789 | -2,94423 |
| 232773_at | --- | --- | 0,00188686 | 0,00504775 | 0,63069 | -0,664996 | -1,58556 |
| 227533_at | --- | --- | 0,0019753 | 0,00517984 | 0,65354 | -0,613653 | -1,53013 |
| 225842_at | 22822 | PHLDA1 | 0,00197973 | 0,00517984 | 2,84324 | 1,50754 | 2,84324 |
| 212713_at | 4239 | MFAP4 | 0,00198588 | 0,00517984 | 0,568757 | -0,814116 | -1,75822 |
| 227027_at | 2673 | GFPT1 | 0,00204733 | 0,00529599 | 1,57296 | 0,653478 | 1,57296 |
| 1553746_a_at | 283310 | OTOGL | 0,00209796 | 0,00536925 | 0,549476 | -0,863873 | -1,81992 |
| 209553_at | 23355 /// 100505729 | LOC100505729 /// VPS8 | 0,00210996 | 0,00536925 | 1,50682 | 0,591511 | 1,50682 |
| 234314_at | 57186 | RALGAPA2 | 0,00214567 | 0,00541609 | 0,594218 | -0,750935 | -1,68288 |
| 213645_at | 55556 | ENOSF1 | 0,00220891 | 0,00552418 | 0,555939 | -0,847001 | -1,79876 |
| 212980_at | 9736 | USP34 | 0,00222379 | 0,00552418 | 0,654515 | -0,611502 | -1,52785 |
| 204811_s_at | 9254 | CACNA2D2 | 0,00233869 | 0,00569003 | 0,588263 | -0,765466 | -1,69992 |
| 225664_at | 1303 | COL12A1 | 0,00234742 | 0,00569003 | 1,80265 | 0,850116 | 1,80265 |
| 235666_at | 8516 | ITGA8 | 0,00236013 | 0,00569003 | 0,64251 | -0,638209 | -1,5564 |
| 206801_at | 4879 | NPPB | 0,00237752 | 0,00569003 | 4,45151 | 2,15429 | 4,45151 |
| 205177_at | 7135 | TNNI1 | 0,00238145 | 0,00569003 | 0,310993 | -1,68505 | -3,21551 |
| 214099_s_at | 9659 /// 728802 /// 100996724 /// 100996761 /// 101060291 /// 101060344 /// 101060353 / | LOC100996724 /// LOC100996761 /// LOC101060291 /// LOC101060344 /// LOC101060353 /// LOC101060463 /// LOC101060582 /// LOC728802 /// PDE4DIP | 0,00242443 | 0,00574884 | 1,51805 | 0,602223 | 1,51805 |
| 228284_at | 7088 | TLE1 | 0,00247741 | 0,00579769 | 1,55201 | 0,634141 | 1,55201 |
| 243016_at | --- | --- | 0,00249264 | 0,00579769 | 0,579981 | -0,785923 | -1,7242 |
| 208096_s_at | 81578 | COL21A1 | 0,0025006 | 0,00579769 | 1,56876 | 0,649623 | 1,56876 |
| 227821_at | 163175 | LGI4 | 0,00259621 | 0,00591858 | 0,651849 | -0,61739 | -1,5341 |
| 209914_s_at | 9378 | NRXN1 | 0,00259843 | 0,00591858 | 0,588849 | -0,76403 | -1,69823 |
| 220994_s_at | 29091 | STXBP6 | 0,00260947 | 0,00591858 | 0,565485 | -0,82244 | -1,76839 |
| 213358_at | 23255 | SOGA2 | 0,00264396 | 0,00595367 | 1,88685 | 0,91598 | 1,88685 |
| 218935_at | 30845 | EHD3 | 0,0027266 | 0,0060959 | 1,67118 | 0,740868 | 1,67118 |
| 1560164_at | --- | OTTHUMG00000015496 /// RP11-532N4.2 | 0,00279541 | 0,00616531 | 2,02166 | 1,01554 | 2,02166 |
| 213524_s_at | 50486 | G0S2 | 0,00279704 | 0,00616531 | 0,617671 | -0,695089 | -1,61898 |
| 219566_at | 79156 | PLEKHF1 | 0,00284421 | 0,00622544 | 0,651665 | -0,617798 | -1,53453 |
| 209596_at | 25878 | MXRA5 | 0,00288577 | 0,00627254 | 2,32619 | 1,21797 | 2,32619 |
| 239093_at | 112817 | HOGA1 | 0,0030168 | 0,00647852 | 1,87919 | 0,910107 | 1,87919 |
| 206042_x_at | 6638 /// 8926 /// 91380 /// 347746 | PAR-SN /// SNORD107 /// SNRPN /// SNURF | 0,00305886 | 0,00647852 | 1,51814 | 0,602303 | 1,51814 |
| 211538_s_at | 3306 | HSPA2 | 0,00307397 | 0,00647852 | 1,73513 | 0,79504 | 1,73513 |
| 205433_at | 590 | BCHE | 0,00309784 | 0,00647852 | 0,567493 | -0,817325 | -1,76214 |
| 205910_s_at | 1056 | CEL | 0,0031028 | 0,00647852 | 0,501089 | -0,99686 | -1,99565 |
| 212912_at | 6196 | RPS6KA2 | 0,00313218 | 0,00647852 | 1,52381 | 0,607682 | 1,52381 |
| 230645_at | 257019 | FRMD3 | 0,00313992 | 0,00647852 | 1,59103 | 0,66996 | 1,59103 |
| 236304_at | --- | --- | 0,00314612 | 0,00647852 | 2,37454 | 1,24765 | 2,37454 |
| 219389_at | 55061 | SUSD4 | 0,00320885 | 0,00656451 | 0,600648 | -0,735408 | -1,66487 |
| 205478_at | 5502 | PPP1R1A | 0,00339748 | 0,00690527 | 0,504277 | -0,987711 | -1,98304 |
| 209840_s_at | 54674 | LRRN3 | 0,00342179 | 0,00690981 | 0,500854 | -0,997539 | -1,99659 |
| 218425_at | 54476 | RNF216 | 0,00358629 | 0,00719557 | 1,71249 | 0,776099 | 1,71249 |
| 240689_at | 283677 | C15orf60 | 0,00373641 | 0,00742884 | 0,664964 | -0,588652 | -1,50384 |
| 1560025_at | 100996667 | LOC100996667 | 0,00375002 | 0,00742884 | 0,545442 | -0,874502 | -1,83337 |
| 204143_s_at | 55556 | ENOSF1 | 0,00378877 | 0,0074584 | 0,619798 | -0,690129 | -1,61343 |
| 244655_at | 100507311 | LOC100507311 | 0,00384243 | 0,00747363 | 0,652322 | -0,616344 | -1,53299 |
| 1559419_at | 783 | CACNB2 | 0,00386091 | 0,00747363 | 0,581327 | -0,782579 | -1,7202 |
| 203629_s_at | 10466 | COG5 | 0,00386814 | 0,00747363 | 0,58042 | -0,78483 | -1,72289 |
| 200605_s_at | 5573 | PRKAR1A | 0,00394777 | 0,00758069 | 1,55589 | 0,63774 | 1,55589 |
| 222020_s_at | 50863 /// 100653217 | LOC100653217 /// NTM | 0,00399533 | 0,00762523 | 0,476519 | -1,06939 | -2,09855 |
| 222784_at | 64093 | SMOC1 | 0,00406158 | 0,00770469 | 0,663866 | -0,591035 | -1,50633 |
| 241968_at | 80167 | C4orf29 | 0,00432642 | 0,00815765 | 1,61346 | 0,690157 | 1,61346 |
| 226856_at | 389125 /// 100526772 | MUSTN1 /// TMEM110-MUSTN1 | 0,00442222 | 0,00828835 | 0,592675 | -0,754686 | -1,68726 |
| 215184_at | 23604 | DAPK2 | 0,00458556 | 0,00849338 | 0,597519 | -0,742944 | -1,67359 |
| 213456_at | 25928 | SOSTDC1 | 0,00461171 | 0,00849338 | 0,624722 | -0,678714 | -1,60071 |
| 238029_s_at | 151473 | SLC16A14 | 0,00461302 | 0,00849338 | 0,619576 | -0,690647 | -1,61401 |
| 202998_s_at | 4017 | LOXL2 | 0,00466396 | 0,00853696 | 1,51577 | 0,60005 | 1,51577 |
| 203798_s_at | 7447 | VSNL1 | 0,0047793 | 0,00869721 | 1,61624 | 0,692644 | 1,61624 |
| 218330_s_at | 89797 | NAV2 | 0,00502494 | 0,00903754 | 1,64901 | 0,721602 | 1,64901 |
| 209614_at | 125 | ADH1B | 0,00502844 | 0,00903754 | 0,575873 | -0,796178 | -1,73649 |
| 230577_at | 100507008 | LINC00844 | 0,00505294 | 0,00903754 | 0,492294 | -1,02241 | -2,03131 |
| 204537_s_at | 2564 /// 407009 /// 574412 | GABRE /// MIR224 /// MIR452 | 0,00510067 | 0,00907108 | 0,663194 | -0,592498 | -1,50786 |
| 214265_at | 8516 | ITGA8 | 0,00513572 | 0,00908181 | 0,558393 | -0,840647 | -1,79085 |
| 232628_at | --- | --- | 0,00516579 | 0,00908366 | 0,63566 | -0,653674 | -1,57317 |
| 1569975_at | --- | --- | 0,00520006 | 0,00909284 | 0,647703 | -0,626596 | -1,54392 |
| 228194_s_at | 114815 | SORCS1 | 0,00523289 | 0,00909941 | 0,651487 | -0,618193 | -1,53495 |
| 231048_at | --- | --- | 0,00536964 | 0,00928562 | 0,584144 | -0,775604 | -1,71191 |
| 228783_at | 11149 | BVES | 0,00544984 | 0,00937253 | 1,64291 | 0,716254 | 1,64291 |
| 214357_at | 92346 | C1orf105 | 0,00551785 | 0,00943763 | 0,509937 | -0,971609 | -1,96103 |
| 220116_at | 3781 | KCNN2 | 0,00566827 | 0,00959279 | 0,607589 | -0,718832 | -1,64585 |
| 213135_at | 7074 | TIAM1 | 0,00566986 | 0,00959279 | 0,633056 | -0,659595 | -1,57964 |
| 231781_s_at | 79442 | LRRC2 | 0,00577605 | 0,00971991 | 1,61089 | 0,687858 | 1,61089 |
| 231040_at | --- | --- | 0,00584654 | 0,00978592 | 0,658186 | -0,603432 | -1,51933 |
| 206806_at | 9162 | DGKI | 0,00597288 | 0,00994421 | 1,72245 | 0,784461 | 1,72245 |
| 230384_at | 200539 | ANKRD23 | 0,00607251 | 0,00997026 | 0,644744 | -0,633202 | -1,551 |
| 219949_at | 79442 | LRRC2 | 0,00607329 | 0,00997026 | 1,71975 | 0,782195 | 1,71975 |
| 1558444_at | --- | --- | 0,00608409 | 0,00997026 | 0,649544 | -0,6225 | -1,53954 |
| 204755_x_at | 3131 | HLF | 0,00616583 | 0,0099737 | 0,656281 | -0,607615 | -1,52374 |
| 1559420_x_at | 783 | CACNB2 | 0,0061776 | 0,0099737 | 0,634804 | -0,655617 | -1,57529 |
| 210198_s_at | 5354 | PLP1 | 0,00618178 | 0,0099737 | 0,550864 | -0,860233 | -1,81533 |
| 224823_at | 4638 | MYLK | 0,00631669 | 0,0101391 | 0,548654 | -0,86603 | -1,82264 |
| 230509_at | 79856 | SNX22 | 0,00635718 | 0,01015203 | 0,653834 | -0,613004 | -1,52944 |
| 226086_at | 57586 | SYT13 | 0,00647011 | 0,01022501 | 0,48471 | -1,04481 | -2,06309 |
| 240395_at | 100128727 | LOC100128727 | 0,00649274 | 0,01022501 | 1,98427 | 0,988612 | 1,98427 |
| 243189_at | 4899 | NRF1 | 0,00653105 | 0,01022501 | 0,600222 | -0,736432 | -1,66605 |
| 239963_at | --- | --- | 0,00653355 | 0,01022501 | 1,50514 | 0,589897 | 1,50514 |
| 210147_at | 419 | ART3 | 0,00657082 | 0,01023217 | 0,650502 | -0,620374 | -1,53727 |
| 207961_x_at | 4629 | MYH11 | 0,00661477 | 0,01024962 | 0,661637 | -0,595888 | -1,5114 |
| 201952_at | 214 | ALCAM | 0,00679473 | 0,0104766 | 0,548024 | -0,867689 | -1,82474 |
| 1564358_at | --- | --- | 0,00698114 | 0,01071126 | 0,613426 | -0,705038 | -1,63019 |
| 221584_s_at | 3778 | KCNMA1 | 0,0070584 | 0,01077697 | 0,656166 | -0,607868 | -1,52401 |
| 203766_s_at | 25802 | LMOD1 | 0,00714082 | 0,01080907 | 0,620345 | -0,688858 | -1,61201 |
| 204591_at | 10752 | CHL1 | 0,00714849 | 0,01080907 | 0,647232 | -0,627646 | -1,54504 |
| 239348_at | --- | --- | 0,00722564 | 0,0108732 | 0,621077 | -0,687155 | -1,61011 |
| 205945_at | 3570 | IL6R | 0,00731606 | 0,01095659 | 0,650946 | -0,619391 | -1,53623 |
| 223500_at | 10815 | CPLX1 | 0,00738196 | 0,01100264 | 1,76365 | 0,818561 | 1,76365 |
| 228108_at | 151742 | PPM1L | 0,00760464 | 0,01128082 | 0,655488 | -0,60936 | -1,52558 |
| 1564469_at | 56203 | LMOD3 | 0,0077942 | 0,01150747 | 1,53679 | 0,61992 | 1,53679 |
| 232257_s_at | --- | --- | 0,00783815 | 0,01151803 | 1,54681 | 0,629299 | 1,54681 |
| 205382_s_at | 1675 | CFD | 0,0079371 | 0,01158093 | 0,477409 | -1,0667 | -2,09464 |
| 218967_s_at | 9317 | PTER | 0,00795495 | 0,01158093 | 0,624928 | -0,678238 | -1,60018 |
| 207344_at | 10566 | AKAP3 | 0,00815531 | 0,01181765 | 0,501357 | -0,996089 | -1,99459 |
| 204682_at | 4053 | LTBP2 | 0,00843472 | 0,01216621 | 1,91034 | 0,933828 | 1,91034 |
| 235849_at | 286133 | SCARA5 | 0,00867949 | 0,01238483 | 0,583041 | -0,77833 | -1,71514 |
| 201506_at | 7045 | TGFBI | 0,00869084 | 0,01238483 | 1,51789 | 0,602064 | 1,51789 |
| 219865_at | 29092 | LINC00339 | 0,00870499 | 0,01238483 | 1,51069 | 0,595206 | 1,51069 |
| 202274_at | 72 | ACTG2 | 0,00892643 | 0,01264241 | 0,440595 | -1,18248 | -2,26966 |
| 229580_at | --- | OTTHUMG00000175814 /// RP11-13L2.4 | 0,00898146 | 0,01266305 | 0,554346 | -0,851142 | -1,80393 |
| 202454_s_at | 2065 | ERBB3 | 0,0092029 | 0,01288878 | 0,65971 | -0,600096 | -1,51582 |
| 232568_at | 158295 | MGC24103 | 0,00922392 | 0,01288878 | 0,633148 | -0,659385 | -1,57941 |
| 1557149_at | --- | OTTHUMG00000019290 /// RP11-432J24.5 | 0,00931929 | 0,01296417 | 1,56236 | 0,643731 | 1,56236 |
| 201497_x_at | 4629 | MYH11 | 0,00943213 | 0,01306308 | 0,636993 | -0,650651 | -1,56988 |
| 223690_at | 4053 | LTBP2 | 0,00961793 | 0,01326173 | 1,86816 | 0,901617 | 1,86816 |
| 230417_at | 57452 | GALNT16 | 0,00972803 | 0,0133537 | 0,581642 | -0,781796 | -1,71927 |
| 1554789_a_at | 8622 | PDE8B | 0,00976996 | 0,0133537 | 1,54191 | 0,624723 | 1,54191 |
| 1553970_s_at | 1056 | CEL | 0,00992142 | 0,01350176 | 0,535505 | -0,901027 | -1,8674 |
| 1559003_a_at | 126661 | CCDC163P | 0,00998243 | 0,01352598 | 0,592696 | -0,754636 | -1,68721 |
| 209670_at | 28755 | TRAC | 0,0101373 | 0,01367662 | 0,532342 | -0,909575 | -1,87849 |
| 204337_at | 5999 | RGS4 | 0,0104224 | 0,0139606 | 1,8579 | 0,893671 | 1,8579 |
| 218678_at | 10763 | NES | 0,010437 | 0,0139606 | 1,58276 | 0,662447 | 1,58276 |
| 226444_at | --- | --- | 0,0105361 | 0,01403319 | 0,657559 | -0,604808 | -1,52078 |
| 232206_at | 54986 | ULK4 | 0,0108913 | 0,01444482 | 0,661873 | -0,595374 | -1,51086 |
| 229160_at | 139221 | MUM1L1 | 0,0109611 | 0,01447605 | 0,585575 | -0,772074 | -1,70772 |
| 205782_at | 2252 | FGF7 | 0,0110891 | 0,01457289 | 0,597806 | -0,74225 | -1,67278 |
| 202722_s_at | 2673 | GFPT1 | 0,0111309 | 0,01457289 | 1,56152 | 0,642952 | 1,56152 |
| 214912_at | --- | --- | 0,0111741 | 0,01457289 | 0,650158 | -0,621139 | -1,53809 |
| 235129_at | 5502 | PPP1R1A | 0,011921 | 0,01548246 | 0,565366 | -0,822742 | -1,76876 |
| 233691_at | --- | --- | 0,011975 | 0,01548833 | 1,63609 | 0,71025 | 1,63609 |
| 242052_at | --- | --- | 0,0123067 | 0,01585184 | 1,51067 | 0,595188 | 1,51067 |
| 213992_at | 1288 | COL4A6 | 0,0124623 | 0,01593564 | 0,653208 | -0,614385 | -1,53091 |
| 208767_s_at | 55353 | LAPTM4B | 0,0124972 | 0,01593564 | 1,57289 | 0,653422 | 1,57289 |
| 218345_at | 55365 | TMEM176A | 0,0125245 | 0,01593564 | 0,638613 | -0,646987 | -1,56589 |
| 202555_s_at | 4638 | MYLK | 0,0126115 | 0,01598137 | 0,574299 | -0,800126 | -1,74125 |
| 226615_at | 9213 | XPR1 | 0,0132348 | 0,0167036 | 1,53498 | 0,618223 | 1,53498 |
| 205713_s_at | 1311 | COMP | 0,0133323 | 0,01672447 | 2,12171 | 1,08522 | 2,12171 |
| 203348_s_at | 2119 | ETV5 | 0,0133582 | 0,01672447 | 1,5618 | 0,643212 | 1,5618 |
| 223836_at | 83888 | FGFBP2 | 0,0138179 | 0,0172032 | 0,633921 | -0,657625 | -1,57748 |
| 206204_at | 2888 | GRB14 | 0,0138505 | 0,0172032 | 0,645565 | -0,631366 | -1,54903 |
| 209763_at | 91851 | CHRDL1 | 0,0140686 | 0,01734969 | 0,53741 | -0,895904 | -1,86078 |
| 244029_at | 100131043 | LOC100131043 | 0,0140793 | 0,01734969 | 0,605741 | -0,723228 | -1,65087 |
| 240971_x_at | --- | --- | 0,0147569 | 0,01811337 | 0,648389 | -0,625068 | -1,54228 |
| 202718_at | 3485 | IGFBP2 | 0,0150289 | 0,01837518 | 2,55988 | 1,35608 | 2,55988 |
| 243918_at | --- | --- | 0,0151384 | 0,01843704 | 1,50255 | 0,587411 | 1,50255 |
| 1556842_at | 286087 | LOC286087 | 0,0152306 | 0,01847743 | 0,517878 | -0,949315 | -1,93096 |
| 206324_s_at | 23604 | DAPK2 | 0,0163988 | 0,01981785 | 0,660901 | -0,597493 | -1,51309 |
| 218872_at | 54997 | TESC | 0,0170929 | 0,02057722 | 0,640264 | -0,643262 | -1,56186 |
| 205554_s_at | 1776 | DNASE1L3 | 0,0172937 | 0,0207375 | 0,567226 | -0,818006 | -1,76297 |
| 214433_s_at | 8991 | SELENBP1 | 0,017383 | 0,0207375 | 0,623054 | -0,68257 | -1,605 |
| 205316_at | 6565 | SLC15A2 | 0,0174248 | 0,0207375 | 0,658952 | -0,601755 | -1,51756 |
| 206186_at | 4356 | MPP3 | 0,0177988 | 0,02110237 | 0,637627 | -0,649215 | -1,56831 |
| 205923_at | 5649 | RELN | 0,0182325 | 0,02153499 | 2,2898 | 1,19522 | 2,2898 |
| 223822_at | 55061 | SUSD4 | 0,0186886 | 0,02199072 | 0,588789 | -0,764177 | -1,6984 |
| 221795_at | 4915 | NTRK2 | 0,0192479 | 0,02253472 | 0,603349 | -0,728935 | -1,65742 |
| 223467_at | 51655 | RASD1 | 0,0192949 | 0,02253472 | 0,483792 | -1,04754 | -2,067 |
| 204776_at | 7060 | THBS4 | 0,0193874 | 0,02255857 | 1,76123 | 0,816581 | 1,76123 |
| 215506_s_at | 9077 | DIRAS3 | 0,0197536 | 0,02289954 | 2,05749 | 1,04088 | 2,05749 |
| 209755_at | 23057 | NMNAT2 | 0,0199133 | 0,02299949 | 1,57428 | 0,654689 | 1,57428 |
| 202310_s_at | 1277 | COL1A1 | 0,0203632 | 0,02343265 | 1,85353 | 0,890278 | 1,85353 |
| 207332_s_at | 7037 | TFRC | 0,0213195 | 0,02444324 | 1,57285 | 0,653382 | 1,57285 |
| 220484_at | 55283 | MCOLN3 | 0,0222422 | 0,02540806 | 0,642691 | -0,637803 | -1,55596 |
| 236034_at | 285 | ANGPT2 | 0,0223247 | 0,02540957 | 1,54998 | 0,632249 | 1,54998 |
| 227189_at | 57699 | CPNE5 | 0,0226592 | 0,02569685 | 0,580814 | -0,783852 | -1,72172 |
| 217028_at | 7852 | CXCR4 | 0,0230516 | 0,02604748 | 1,59114 | 0,670064 | 1,59114 |
| 209612_s_at | 125 | ADH1B | 0,0234538 | 0,02640662 | 0,5669 | -0,818834 | -1,76398 |
| 209613_s_at | 125 | ADH1B | 0,023925 | 0,02684059 | 0,555911 | -0,847074 | -1,79885 |
| 227870_at | 57722 | IGDCC4 | 0,0241588 | 0,02700609 | 0,660692 | -0,59795 | -1,51356 |
| 219935_at | 11096 | ADAMTS5 | 0,0251686 | 0,02803478 | 0,665329 | -0,58786 | -1,50302 |
| 202994_s_at | 2192 | FBLN1 | 0,0254764 | 0,028277 | 0,644174 | -0,634477 | -1,55237 |
| 220532_s_at | 28959 | TMEM176B | 0,0256387 | 0,02835658 | 0,590299 | -0,760482 | -1,69406 |
| 228640_at | 5099 | PCDH7 | 0,0263073 | 0,02894196 | 0,642528 | -0,638169 | -1,55635 |
| 204753_s_at | 3131 | HLF | 0,0263529 | 0,02894196 | 0,653745 | -0,613199 | -1,52965 |
| 202311_s_at | 1277 | COL1A1 | 0,0266185 | 0,02913144 | 1,54663 | 0,629126 | 1,54663 |
| 204121_at | 10912 | GADD45G | 0,026925 | 0,0293642 | 0,582514 | -0,779635 | -1,7167 |
| 202404_s_at | 1278 | COL1A2 | 0,0280964 | 0,03053532 | 1,69755 | 0,763452 | 1,69755 |
| 201645_at | 3371 | TNC | 0,0284644 | 0,03082823 | 1,6874 | 0,754801 | 1,6874 |
| 208017_s_at | 4168 | MCF2 | 0,0288907 | 0,03118203 | 1,55833 | 0,640003 | 1,55833 |
| 1556579_s_at | 285313 | IGSF10 | 0,0292848 | 0,03139754 | 0,588566 | -0,764724 | -1,69904 |
| 205493_s_at | 10570 | DPYSL4 | 0,029291 | 0,03139754 | 1,76929 | 0,82317 | 1,76929 |
| 203980_at | 2167 | FABP4 | 0,0298063 | 0,03184086 | 1,75735 | 0,813405 | 1,75735 |
| 209496_at | 5919 | RARRES2 | 0,0306129 | 0,03259128 | 0,604005 | -0,727369 | -1,65562 |
| 211597_s_at | 84525 | HOPX | 0,0312594 | 0,03309611 | 0,466285 | -1,10072 | -2,14461 |
| 1556499_s_at | 1277 | COL1A1 | 0,0313345 | 0,03309611 | 1,70436 | 0,769229 | 1,70436 |
| 219087_at | 54829 | ASPN | 0,0314043 | 0,03309611 | 1,63721 | 0,711238 | 1,63721 |
| 207010_at | 2560 | GABRB1 | 0,032264 | 0,03388803 | 0,65848 | -0,602788 | -1,51865 |
| 226213_at | 2065 | ERBB3 | 0,0330358 | 0,03458263 | 0,665975 | -0,58646 | -1,50156 |
| 214524_at | 2691 | GHRH | 0,0349138 | 0,03642673 | 0,58598 | -0,771076 | -1,70654 |
| 229052_at | 51239 /// 200539 | ANKRD23 /// ANKRD39 | 0,0363117 | 0,03775934 | 0,546698 | -0,871183 | -1,82916 |
| 205048_s_at | 5723 | PSPH | 0,0365713 | 0,03790337 | 0,42583 | -1,23165 | -2,34835 |
| 1554679_a_at | 55353 | LAPTM4B | 0,0368227 | 0,03798059 | 1,72252 | 0,784521 | 1,72252 |
| 204762_s_at | 2775 | GNAO1 | 0,0368885 | 0,03798059 | 0,620327 | -0,688899 | -1,61205 |
| 203549_s_at | 4023 | LPL | 0,0403266 | 0,04138435 | 1,7447 | 0,802978 | 1,7447 |
| 213201_s_at | 7138 | TNNT1 | 0,0409564 | 0,04189331 | 1,61275 | 0,689519 | 1,61275 |
| 1565162_s_at | 4257 | MGST1 | 0,0412389 | 0,04204487 | 0,626689 | -0,674178 | -1,59569 |
| 215076_s_at | 1281 | COL3A1 | 0,043702 | 0,04433133 | 1,64662 | 0,71951 | 1,64662 |
| 242751_at | --- | --- | 0,0437648 | 0,04433133 | 0,665651 | -0,587162 | -1,50229 |
| 234082_at | 100505874 | LOC100505874 | 0,044134 | 0,0445611 | 0,628002 | -0,67116 | -1,59235 |
| 1554663_a_at | 4926 | NUMA1 | 0,0445206 | 0,04480691 | 0,620172 | -0,68926 | -1,61246 |
| 203548_s_at | 4023 | LPL | 0,0451418 | 0,04528649 | 1,85612 | 0,892286 | 1,85612 |
| 230670_at | 285313 | IGSF10 | 0,0460486 | 0,0460486 | 0,593192 | -0,753429 | -1,68579 |
