# Supplementary material for: Molecular Signatures of Human Chronic Atrial Fibrillation in Primary Mitral Regurgitation
Source: Cardiovasc Ther. 2021 Oct 15;2021:5516185. doi: 10.1155/2021/5516185 (PMC8538404; doi:10.1155/2021/5516185)
Supplement: Supplementary 9 — Supplementary Table 8: differentially expressed genes in LAA tissues of AFib vs. SR (fold change > 1.5; P < 0.05 and q < 0.05). [file 5516185.f9.docx]

**Supplementary Table 8:** Differentially expressed genes in LAA tissues of AFib vs SR (Fold Change > 1.5; p<0.05 and q<0.05).

| **Probeset ID** | **Entrez Gene** | **Gene Symbol** | **p-value** | **q value** | **MeanRatio(AF/SR)** | **MeanDiff(AF-SR)** | **FoldChange(AF/SR)** |
| --- | --- | --- | --- | --- | --- | --- | --- |
| 230417_at | 57452 | GALNT16 | 3,43E-06 | 0,000834 | 0,518731 | -0,946943 | -1,92778 |
| 209617_s_at | 1501 | CTNND2 | 6,65E-06 | 0,000834 | 1,51321 | 0,597609 | 1,51321 |
| 230418_s_at | 57452 | GALNT16 | 1,22E-05 | 0,00099793 | 0,491386 | -1,02507 | -2,03506 |
| 230384_at | 200539 | ANKRD23 | 1,77E-05 | 0,00099793 | 0,566632 | -0,819516 | -1,76481 |
| 1555869_a_at | 100507477 | LOC100507477 | 2,03E-05 | 0,00099793 | 0,36425 | -1,457 | -2,74536 |
| 229797_at | 55283 | MCOLN3 | 2,42E-05 | 0,00099793 | 0,318578 | -1,65028 | -3,13895 |
| 230623_x_at | 57646 | USP28 | 2,78E-05 | 0,00099793 | 0,652268 | -0,616464 | -1,53311 |
| 213004_at | 23452 | ANGPTL2 | 7,08E-05 | 0,00160889 | 2,00397 | 1,00286 | 2,00397 |
| 231430_at | 220382 | FAM181B | 7,41E-05 | 0,00160889 | 0,59564 | -0,747488 | -1,67887 |
| 207344_at | 10566 | AKAP3 | 7,41E-05 | 0,00160889 | 0,357277 | -1,48489 | -2,79895 |
| 240395_at | 100128727 | LOC100128727 | 8,19E-05 | 0,00160889 | 2,24144 | 1,16443 | 2,24144 |
| 1553746_a_at | 283310 | OTOGL | 8,31E-05 | 0,00160889 | 0,546736 | -0,871084 | -1,82904 |
| 215843_s_at | 7093 | TLL2 | 9,07E-05 | 0,00160889 | 0,496879 | -1,00903 | -2,01256 |
| 243730_at | --- | --- | 9,45E-05 | 0,00160889 | 0,62478 | -0,678581 | -1,60056 |
| 206638_at | 3357 | HTR2B | 9,61E-05 | 0,00160889 | 1,59959 | 0,677701 | 1,59959 |
| 209921_at | 23657 | SLC7A11 | 0,00013485 | 0,00211544 | 0,50111 | -0,996801 | -1,99557 |
| 230560_at | 29091 | STXBP6 | 0,00016523 | 0,00243963 | 0,502432 | -0,993 | -1,99032 |
| 214369_s_at | 10235 | RASGRP2 | 0,0002312 | 0,00322391 | 0,642711 | -0,637757 | -1,55591 |
| 204260_at | 1114 | CHGB | 0,00026698 | 0,00352695 | 2,38555 | 1,25432 | 2,38555 |
| 213456_at | 25928 | SOSTDC1 | 0,00028982 | 0,0036372 | 0,633678 | -0,658177 | -1,57809 |
| 219338_s_at | 54839 | LRRC49 | 0,00035896 | 0,00429045 | 0,449915 | -1,15228 | -2,22264 |
| 205970_at | 4504 | MT3 | 0,00041883 | 0,00477852 | 0,637271 | -0,650021 | -1,56919 |
| 201995_at | 2131 | EXT1 | 0,00052153 | 0,00544357 | 1,67168 | 0,741297 | 1,67168 |
| 214357_at | 92346 | C1orf105 | 0,00053327 | 0,00544357 | 0,422658 | -1,24244 | -2,36598 |
| 229052_at | 51239 /// 200539 | ANKRD23 /// ANKRD39 | 0,00054219 | 0,00544357 | 0,446649 | -1,16279 | -2,2389 |
| 222919_at | 10345 | TRDN | 0,00057155 | 0,00551768 | 0,437696 | -1,192 | -2,28469 |
| 231040_at | --- | --- | 0,00060751 | 0,0055685 | 0,631523 | -0,663093 | -1,58347 |
| 220484_at | 55283 | MCOLN3 | 0,00062119 | 0,0055685 | 0,558517 | -0,840327 | -1,79046 |
| 227065_at | 54476 | RNF216 | 0,00068425 | 0,00592227 | 1,52956 | 0,613114 | 1,52956 |
| 227819_at | 59352 | LGR6 | 0,00075592 | 0,00632451 | 0,56816 | -0,81563 | -1,76007 |
| 219142_at | 65997 | RASL11B | 0,00079287 | 0,00640509 | 1,6635 | 0,734226 | 1,6635 |
| 206806_at | 9162 | DGKI | 0,00081659 | 0,00640509 | 1,69273 | 0,759351 | 1,69273 |
| 209590_at | 655 | BMP7 | 0,00086685 | 0,00659331 | 0,650094 | -0,621281 | -1,53824 |
| 1554789_a_at | 8622 | PDE8B | 0,00094113 | 0,00679925 | 2,02794 | 1,02001 | 2,02794 |
| 230577_at | 100507008 | LINC00844 | 0,0009481 | 0,00679925 | 0,397044 | -1,33263 | -2,51861 |
| 230509_at | 79856 | SNX22 | 0,00106585 | 0,00727832 | 0,5761 | -0,795608 | -1,73581 |
| 202718_at | 3485 | IGFBP2 | 0,0010729 | 0,00727832 | 2,64036 | 1,40073 | 2,64036 |
| 227401_at | 53342 | IL17D | 0,00127393 | 0,00789168 | 1,65871 | 0,730065 | 1,65871 |
| 213228_at | 8622 | PDE8B | 0,00127843 | 0,00789168 | 2,10489 | 1,07375 | 2,10489 |
| 205478_at | 5502 | PPP1R1A | 0,00130552 | 0,00789168 | 0,557323 | -0,843415 | -1,79429 |
| 209875_s_at | 6696 | SPP1 | 0,00130972 | 0,00789168 | 2,71885 | 1,443 | 2,71885 |
| 213761_at | 56890 | MDM1 | 0,00132052 | 0,00789168 | 0,615382 | -0,700447 | -1,62501 |
| 213001_at | 23452 | ANGPTL2 | 0,00144695 | 0,00844615 | 2,11725 | 1,08219 | 2,11725 |
| 229302_at | 130733 | TMEM178A | 0,00148138 | 0,0084506 | 0,605214 | -0,724484 | -1,65231 |
| 231048_at | --- | --- | 0,00155599 | 0,00867897 | 0,566644 | -0,819486 | -1,76478 |
| 243810_at | --- | --- | 0,001633 | 0,0089105 | 2,50176 | 1,32294 | 2,50176 |
| 219789_at | 4883 | NPR3 | 0,00173093 | 0,0092439 | 1,80607 | 0,852851 | 1,80607 |
| 240339_at | --- | --- | 0,00177975 | 0,00930661 | 0,595071 | -0,748865 | -1,68047 |
| 239474_at | --- | --- | 0,00182634 | 0,00935533 | 1,79968 | 0,847743 | 1,79968 |
| 236184_at | --- | --- | 0,00194018 | 0,0097397 | 1,6309 | 0,70567 | 1,6309 |
| 212587_s_at | 5788 | PTPRC | 0,00210393 | 0,0102737 | 1,90827 | 0,932267 | 1,90827 |
| 235649_at | 11095 | ADAMTS8 | 0,00215679 | 0,0102737 | 0,625984 | -0,675803 | -1,59749 |
| 209841_s_at | 54674 | LRRN3 | 0,00219315 | 0,0102737 | 0,449383 | -1,15398 | -2,22528 |
| 226065_at | 144165 | PRICKLE1 | 0,00223863 | 0,0102737 | 1,7029 | 0,767992 | 1,7029 |
| 230508_at | 27122 | DKK3 | 0,00225394 | 0,0102737 | 1,70648 | 0,771027 | 1,70648 |
| 208322_s_at | 6482 | ST3GAL1 | 0,00229214 | 0,0102737 | 1,5144 | 0,598747 | 1,5144 |
| 1560164_at | --- | OTTHUMG00000015496 /// RP11-532N4.2 | 0,00263908 | 0,01162121 | 2,00158 | 1,00114 | 2,00158 |
| 239882_at | --- | OTTHUMG00000015620 /// RP11-557H15.4 | 0,00272124 | 0,01163623 | 1,65591 | 0,727623 | 1,65591 |
| 228754_at | 6533 | SLC6A6 | 0,00276701 | 0,01163623 | 2,01386 | 1,00996 | 2,01386 |
| 229831_at | 5067 | CNTN3 | 0,00278157 | 0,01163623 | 0,500623 | -0,998205 | -1,99751 |
| 235759_at | --- | --- | 0,00294619 | 0,01212285 | 0,6191 | -0,691755 | -1,61525 |
| 206768_at | 6123 | RPL3L | 0,00303151 | 0,01217779 | 1,6351 | 0,709378 | 1,6351 |
| 228108_at | 151742 | PPM1L | 0,00311545 | 0,01217779 | 0,558661 | -0,839956 | -1,79 |
| 227984_at | 64788 | LMF1 | 0,00314832 | 0,01217779 | 1,6765 | 0,745457 | 1,6765 |
| 223952_x_at | 10170 | DHRS9 | 0,00315361 | 0,01217779 | 1,98352 | 0,988065 | 1,98352 |
| 211143_x_at | 3164 | NR4A1 | 0,00326838 | 0,01230531 | 0,653672 | -0,613361 | -1,52982 |
| 1553970_s_at | 1056 | CEL | 0,00328886 | 0,01230531 | 0,585894 | -0,771289 | -1,70679 |
| 218425_at | 54476 | RNF216 | 0,00333371 | 0,01230531 | 1,72455 | 0,786221 | 1,72455 |
| 223438_s_at | 5465 | PPARA | 0,00356516 | 0,01296892 | 1,60899 | 0,686155 | 1,60899 |
| 220994_s_at | 29091 | STXBP6 | 0,00368328 | 0,01320719 | 0,641759 | -0,639897 | -1,55822 |
| 202728_s_at | 4052 | LTBP1 | 0,0037454 | 0,01324078 | 2,02261 | 1,01622 | 2,02261 |
| 213967_at | 138046 | RALYL | 0,00388639 | 0,01354839 | 0,661757 | -0,595626 | -1,51113 |
| 231384_at | 2903 | GRIN2A | 0,00402931 | 0,0138542 | 0,655645 | -0,609014 | -1,52522 |
| 223280_x_at | 64231 | MS4A6A | 0,00422855 | 0,01434278 | 1,53567 | 0,618873 | 1,53567 |
| 209127_s_at | 9733 | SART3 | 0,00443346 | 0,01483731 | 1,60422 | 0,681876 | 1,60422 |
| 205910_s_at | 1056 | CEL | 0,00461773 | 0,01492153 | 0,617772 | -0,694854 | -1,61872 |
| 206115_at | 1960 | EGR3 | 0,00462904 | 0,01492153 | 0,557864 | -0,842014 | -1,79255 |
| 205952_at | 3777 | KCNK3 | 0,00463697 | 0,01492153 | 1,71398 | 0,777348 | 1,71398 |
| 203629_s_at | 10466 | COG5 | 0,00484783 | 0,0154026 | 0,625613 | -0,676658 | -1,59843 |
| 219865_at | 29092 | LINC00339 | 0,00493254 | 0,01547584 | 1,54312 | 0,625854 | 1,54312 |
| 205713_s_at | 1311 | COMP | 0,00505162 | 0,01565379 | 2,63687 | 1,39883 | 2,63687 |
| 213272_s_at | 57146 | TMEM159 | 0,00536577 | 0,01642449 | 1,61487 | 0,69142 | 1,61487 |
| 222927_s_at | 594855 | CPLX3 | 0,00553962 | 0,01675234 | 0,504016 | -0,988457 | -1,98406 |
| 243737_at | 23439 | ATP1B4 | 0,00566168 | 0,01691764 | 2,87443 | 1,52328 | 2,87443 |
| 208370_s_at | 1827 | RCAN1 | 0,00574937 | 0,01697755 | 1,66274 | 0,733566 | 1,66274 |
| 223822_at | 55061 | SUSD4 | 0,0060104 | 0,01716829 | 0,611275 | -0,710106 | -1,63592 |
| 209774_x_at | 2920 | CXCL2 | 0,00602878 | 0,01716829 | 0,379902 | -1,3963 | -2,63226 |
| 202340_x_at | 3164 | NR4A1 | 0,00607018 | 0,01716829 | 0,553981 | -0,852091 | -1,80512 |
| 201694_s_at | 1958 | EGR1 | 0,00608756 | 0,01716829 | 0,404021 | -1,3075 | -2,47512 |
| 208998_at | 7351 | UCP2 | 0,0064304 | 0,01793367 | 1,63669 | 0,71078 | 1,63669 |
| 207468_s_at | 6425 | SFRP5 | 0,00661311 | 0,0179634 | 0,586441 | -0,769941 | -1,7052 |
| 235129_at | 5502 | PPP1R1A | 0,00665311 | 0,0179634 | 0,576902 | -0,793602 | -1,7334 |
| 202393_s_at | 7071 | KLF10 | 0,00665576 | 0,0179634 | 0,607812 | -0,718302 | -1,64525 |
| 202436_s_at | 1545 | CYP1B1 | 0,00679375 | 0,01802257 | 2,17537 | 1,12126 | 2,17537 |
| 202672_s_at | 467 | ATF3 | 0,00682129 | 0,01802257 | 0,33097 | -1,59523 | -3,02143 |
| 242286_at | 2903 | GRIN2A | 0,00695894 | 0,01809114 | 0,558066 | -0,841493 | -1,7919 |
| 202709_at | 2331 | FMOD | 0,00701458 | 0,01809114 | 1,75087 | 0,80807 | 1,75087 |
| 217763_s_at | 11031 | RAB31 | 0,00712487 | 0,01809114 | 1,57416 | 0,654584 | 1,57416 |
| 1557292_a_at | 55283 | MCOLN3 | 0,00713555 | 0,01809114 | 0,620762 | -0,687889 | -1,61092 |
| 226228_at | 361 | AQP4 | 0,00731875 | 0,01820845 | 0,664933 | -0,588719 | -1,50391 |
| 221288_at | 2845 | GPR22 | 0,007329 | 0,01820845 | 0,601595 | -0,733134 | -1,66225 |
| 202435_s_at | 1545 | CYP1B1 | 0,00739945 | 0,01820845 | 1,80635 | 0,85308 | 1,80635 |
| 242271_at | 115019 | SLC26A9 | 0,00754516 | 0,01838109 | 0,620508 | -0,688477 | -1,61158 |
| 206186_at | 4356 | MPP3 | 0,00761607 | 0,01838109 | 0,594053 | -0,751336 | -1,68335 |
| 1555958_at | 55118 | CRTAC1 | 0,00770847 | 0,01842691 | 0,660637 | -0,59807 | -1,51369 |
| 213661_at | 25891 | PAMR1 | 0,00803221 | 0,01859218 | 1,58032 | 0,660214 | 1,58032 |
| 201944_at | 3074 | HEXB | 0,00806053 | 0,01859218 | 1,59819 | 0,676437 | 1,59819 |
| 214087_s_at | 4604 | MYBPC1 | 0,00807163 | 0,01859218 | 0,581454 | -0,782262 | -1,71983 |
| 205493_s_at | 10570 | DPYSL4 | 0,00814443 | 0,01859218 | 2,03778 | 1,027 | 2,03778 |
| 208146_s_at | 54504 | CPVL | 0,00817944 | 0,01859218 | 1,70362 | 0,768605 | 1,70362 |
| 201464_x_at | 3725 | JUN | 0,00822204 | 0,01859218 | 0,632574 | -0,660693 | -1,58084 |
| 223377_x_at | 1154 | CISH | 0,00830096 | 0,01860304 | 1,69136 | 0,758187 | 1,69136 |
| 201911_s_at | 10160 /// 101060526 | FARP1 /// FARP1-IT1 | 0,0084846 | 0,01884632 | 1,53518 | 0,618404 | 1,53518 |
| 204591_at | 10752 | CHL1 | 0,00856101 | 0,01884924 | 0,635415 | -0,654228 | -1,57377 |
| 209693_at | 23245 | ASTN2 | 0,00895587 | 0,01941519 | 0,665291 | -0,587943 | -1,5031 |
| 204446_s_at | 240 | ALOX5 | 0,00899052 | 0,01941519 | 1,59393 | 0,672587 | 1,59393 |
| 229139_at | 56704 | JPH1 | 0,0091111 | 0,01941519 | 0,665812 | -0,586813 | -1,50193 |
| 218935_at | 30845 | EHD3 | 0,0091747 | 0,01941519 | 1,5274 | 0,611074 | 1,5274 |
| 206073_at | 8292 | COLQ | 0,00920481 | 0,01941519 | 2,0818 | 1,05783 | 2,0818 |
| 204121_at | 10912 | GADD45G | 0,00931287 | 0,01947942 | 0,550354 | -0,861569 | -1,81701 |
| 219825_at | 56603 | CYP26B1 | 0,0095901 | 0,01989351 | 1,67626 | 0,745244 | 1,67626 |
| 221011_s_at | 81606 | LBH | 0,0102376 | 0,0210626 | 1,75435 | 0,810937 | 1,75435 |
| 205433_at | 590 | BCHE | 0,0105245 | 0,02147683 | 0,487602 | -1,03622 | -2,05085 |
| 230419_at | 400618 | FLJ37644 | 0,0107336 | 0,02172688 | 1,5117 | 0,59617 | 1,5117 |
| 209959_at | 8013 | NR4A3 | 0,0108216 | 0,02172977 | 0,465117 | -1,10433 | -2,14999 |
| 204017_at | 11015 | KDELR3 | 0,0110515 | 0,02173947 | 1,57256 | 0,653114 | 1,57256 |
| 210664_s_at | 7035 | TFPI | 0,0112058 | 0,02173947 | 1,64142 | 0,714946 | 1,64142 |
| 226856_at | 389125 /// 100526772 | MUSTN1 /// TMEM110-MUSTN1 | 0,0112749 | 0,02173947 | 0,614071 | -0,703522 | -1,62848 |
| 223189_x_at | 55904 | KMT2E | 0,0112899 | 0,02173947 | 1,57086 | 0,651554 | 1,57086 |
| 219106_s_at | 10324 | KLHL41 | 0,0112906 | 0,02173947 | 0,60738 | -0,719329 | -1,64642 |
| 224009_x_at | 10170 | DHRS9 | 0,0113461 | 0,02173947 | 1,99326 | 0,995127 | 1,99326 |
| 219775_s_at | 594855 | CPLX3 | 0,0116017 | 0,02206081 | 0,648933 | -0,623858 | -1,54099 |
| 218170_at | 51015 | ISOC1 | 0,0120081 | 0,02263252 | 0,621286 | -0,68667 | -1,60956 |
| 238625_at | 199920 | C1orf168 | 0,0120827 | 0,02263252 | 0,614361 | -0,702841 | -1,62771 |
| 1566509_s_at | 26268 | FBXO9 | 0,0121962 | 0,0226759 | 1,58455 | 0,664073 | 1,58455 |
| 219799_s_at | 10170 | DHRS9 | 0,0125497 | 0,02316158 | 1,73916 | 0,798391 | 1,73916 |
| 209183_s_at | 11067 | C10orf10 | 0,0131891 | 0,02360142 | 1,73384 | 0,793973 | 1,73384 |
| 223200_s_at | 55341 | LSG1 | 0,0132121 | 0,02360142 | 1,58712 | 0,66641 | 1,58712 |
| 238751_at | --- | --- | 0,0132335 | 0,02360142 | 1,64299 | 0,71632 | 1,64299 |
| 202803_s_at | 3689 | ITGB2 | 0,013241 | 0,02360142 | 1,50227 | 0,587142 | 1,50227 |
| 210479_s_at | 6095 | RORA | 0,0133146 | 0,02360142 | 1,77542 | 0,828162 | 1,77542 |
| 221008_s_at | 64850 | ETNPPL | 0,0133522 | 0,02360142 | 0,527111 | -0,923822 | -1,89713 |
| 202323_s_at | 64746 | ACBD3 | 0,0135193 | 0,02372968 | 1,73508 | 0,795002 | 1,73508 |
| 202935_s_at | 6662 | SOX9 | 0,0137014 | 0,0238823 | 1,60585 | 0,68334 | 1,60585 |
| 214730_s_at | 2734 | GLG1 | 0,0140235 | 0,02427516 | 1,64251 | 0,715899 | 1,64251 |
| 205499_at | 27286 | SRPX2 | 0,0142742 | 0,02441974 | 1,58916 | 0,668268 | 1,58916 |
| 204051_s_at | 6424 | SFRP4 | 0,0143016 | 0,02441974 | 1,87645 | 0,908006 | 1,87645 |
| 201141_at | 10457 | GPNMB | 0,0147402 | 0,02498141 | 1,65619 | 0,727866 | 1,65619 |
| 201721_s_at | 7805 | LAPTM5 | 0,0148296 | 0,02498141 | 1,89932 | 0,925479 | 1,89932 |
| 211769_x_at | 10955 | SERINC3 | 0,0150998 | 0,025267 | 1,70268 | 0,767804 | 1,70268 |
| 232206_at | 54986 | ULK4 | 0,0152892 | 0,02535861 | 0,634826 | -0,655568 | -1,57524 |
| 224367_at | 84707 | BEX2 | 0,0155182 | 0,02535861 | 0,634021 | -0,657397 | -1,57723 |
| 204622_x_at | 4929 | NR4A2 | 0,0155523 | 0,02535861 | 0,414483 | -1,27061 | -2,41264 |
| 204384_at | 2801 | GOLGA2 | 0,0156132 | 0,02535861 | 1,67914 | 0,747719 | 1,67914 |
| 204783_at | 4291 | MLF1 | 0,0156597 | 0,02535861 | 0,660492 | -0,598388 | -1,51402 |
| 201693_s_at | 1958 | EGR1 | 0,0157895 | 0,02538489 | 0,483168 | -1,0494 | -2,06967 |
| 1554018_at | 10457 | GPNMB | 0,0158782 | 0,02538489 | 1,55006 | 0,632328 | 1,55006 |
| 201914_s_at | 11231 | SEC63 | 0,0159884 | 0,02539929 | 1,56812 | 0,649038 | 1,56812 |
| 211948_x_at | 23215 | PRRC2C | 0,0161619 | 0,02542128 | 1,50456 | 0,589342 | 1,50456 |
| 222162_s_at | 9510 | ADAMTS1 | 0,0162048 | 0,02542128 | 0,483528 | -1,04833 | -2,06813 |
| 203630_s_at | 10466 | COG5 | 0,0163791 | 0,02553512 | 0,585442 | -0,772402 | -1,70811 |
| 222486_s_at | 9510 | ADAMTS1 | 0,0167897 | 0,02601367 | 0,627051 | -0,673346 | -1,59477 |
| 244447_at | --- | --- | 0,0174479 | 0,02686763 | 0,627426 | -0,672483 | -1,59381 |
| 201531_at | 7538 | ZFP36 | 0,0179149 | 0,02741726 | 0,598058 | -0,741643 | -1,67208 |
| 205374_at | 6588 | SLN | 0,0180233 | 0,02741726 | 0,517835 | -0,949437 | -1,93112 |
| 226210_s_at | 55384 | MEG3 | 0,0187807 | 0,02839732 | 1,68221 | 0,750357 | 1,68221 |
| 204326_x_at | 4501 | MT1X | 0,0189826 | 0,02853073 | 0,596805 | -0,744669 | -1,67559 |
| 221563_at | 11221 | DUSP10 | 0,0196095 | 0,02929753 | 0,628716 | -0,669519 | -1,59054 |
| 204803_s_at | 6236 | RRAD | 0,0206168 | 0,03061388 | 1,57385 | 0,654301 | 1,57385 |
| 219054_at | 4883 | NPR3 | 0,0207345 | 0,03061388 | 1,71728 | 0,780125 | 1,71728 |
| 204122_at | 7305 | TYROBP | 0,0216198 | 0,03160411 | 1,53953 | 0,622493 | 1,53953 |
| 205048_s_at | 5723 | PSPH | 0,021657 | 0,03160411 | 0,388806 | -1,36288 | -2,57197 |
| 225557_at | 64651 | CSRNP1 | 0,0223266 | 0,03209275 | 0,611276 | -0,710103 | -1,63592 |
| 213714_at | 783 | CACNB2 | 0,0223862 | 0,03209275 | 0,636028 | -0,652839 | -1,57226 |
| 213992_at | 1288 | COL4A6 | 0,0224947 | 0,03209275 | 0,651139 | -0,618963 | -1,53577 |
| 216248_s_at | 4929 | NR4A2 | 0,0226117 | 0,03209275 | 0,452342 | -1,14451 | -2,21072 |
| 223818_s_at | 51773 | RSF1 | 0,0226387 | 0,03209275 | 1,50642 | 0,591123 | 1,50642 |
| 226069_at | 144165 | PRICKLE1 | 0,022759 | 0,03209275 | 1,52851 | 0,612124 | 1,52851 |
| 212979_s_at | 9747 /// 100294033 | FAM115A /// LOC100294033 | 0,0233796 | 0,03278368 | 1,52032 | 0,604371 | 1,52032 |
| 212078_s_at | 4297 | KMT2A | 0,0236837 | 0,0330256 | 1,55504 | 0,636955 | 1,55504 |
| 205171_at | 5775 | PTPN4 | 0,0243902 | 0,03382287 | 1,5196 | 0,60369 | 1,5196 |
| 1552712_a_at | 23057 | NMNAT2 | 0,0245282 | 0,03382735 | 1,67567 | 0,744737 | 1,67567 |
| 215646_s_at | 1462 | VCAN | 0,0247204 | 0,03390612 | 1,55815 | 0,639836 | 1,55815 |
| 228067_at | 343990 | KIAA1211L | 0,0252566 | 0,0344533 | 0,654931 | -0,610585 | -1,52688 |
| 210426_x_at | 6095 | RORA | 0,0254953 | 0,03459092 | 1,65638 | 0,728035 | 1,65638 |
| 203869_at | 64854 | USP46 | 0,0261891 | 0,03531154 | 1,64915 | 0,721723 | 1,64915 |
| 1554447_at | 554203 | JPX | 0,0263078 | 0,03531154 | 1,72405 | 0,785798 | 1,72405 |
| 205177_at | 7135 | TNNI1 | 0,0274011 | 0,03658338 | 0,536257 | -0,899003 | -1,86478 |
| 222287_at | 10345 | TRDN | 0,0280089 | 0,037197 | 0,573935 | -0,801041 | -1,74236 |
| 210147_at | 419 | ART3 | 0,0288836 | 0,03815676 | 0,62135 | -0,686521 | -1,6094 |
| 213358_at | 23255 | SOGA2 | 0,0290421 | 0,03816527 | 1,56992 | 0,650687 | 1,56992 |
| 230630_at | 205 /// 100507855 | AK4 /// LOC100507855 | 0,0293036 | 0,03830835 | 1,62302 | 0,698679 | 1,62302 |
| 204223_at | 5549 | PRELP | 0,0296795 | 0,03859873 | 1,56892 | 0,649769 | 1,56892 |
| 201473_at | 3726 | JUNB | 0,0304441 | 0,03938902 | 0,608662 | -0,716287 | -1,64295 |
| 205439_at | 2953 | GSTT2 | 0,0307647 | 0,03959969 | 1,69518 | 0,761435 | 1,69518 |
| 222834_s_at | 55970 | GNG12 | 0,031057 | 0,03977197 | 1,59879 | 0,676983 | 1,59879 |
| 236352_at | 245806 | VGLL2 | 0,0317833 | 0,04031672 | 1,55337 | 0,635399 | 1,55337 |
| 219558_at | 79572 | ATP13A3 | 0,0318647 | 0,04031672 | 1,76531 | 0,819924 | 1,76531 |
| 221473_x_at | 10955 | SERINC3 | 0,0322237 | 0,04031672 | 1,50017 | 0,585131 | 1,50017 |
| 228335_at | 5010 | CLDN11 | 0,0322419 | 0,04031672 | 1,52579 | 0,609555 | 1,52579 |
| 220556_at | 23439 | ATP1B4 | 0,0322855 | 0,04031672 | 1,53919 | 0,622171 | 1,53919 |
| 213385_at | 1124 | CHN2 | 0,0325023 | 0,04038652 | 1,77868 | 0,830807 | 1,77868 |
| 205357_s_at | 185 | AGTR1 | 0,0329437 | 0,04062718 | 0,596244 | -0,746026 | -1,67717 |
| 201072_s_at | 6599 | SMARCC1 | 0,0330197 | 0,04062718 | 1,52116 | 0,605168 | 1,52116 |
| 209670_at | 28755 | TRAC | 0,033678 | 0,04104837 | 0,544523 | -0,876935 | -1,83647 |
| 228948_at | 2043 | EPHA4 | 0,0336891 | 0,04104837 | 1,50379 | 0,588599 | 1,50379 |
| 214038_at | 6355 | CCL8 | 0,0339693 | 0,04110004 | 0,653981 | -0,612679 | -1,5291 |
| 206315_at | 9244 | CRLF1 | 0,034059 | 0,04110004 | 1,53101 | 0,614486 | 1,53101 |
| 202934_at | 3099 | HK2 | 0,0347525 | 0,04162523 | 1,50791 | 0,592551 | 1,50791 |
| 202905_x_at | 4683 | NBN | 0,034828 | 0,04162523 | 1,65425 | 0,726179 | 1,65425 |
| 209024_s_at | 10492 | SYNCRIP | 0,0351464 | 0,04162523 | 1,617 | 0,693318 | 1,617 |
| 204682_at | 4053 | LTBP2 | 0,035315 | 0,04162523 | 1,67311 | 0,742534 | 1,67311 |
| 1559419_at | 783 | CACNB2 | 0,0353234 | 0,04162523 | 0,622919 | -0,682883 | -1,60534 |
| 238013_at | 59339 | PLEKHA2 | 0,0355944 | 0,04174857 | 1,67773 | 0,746511 | 1,67773 |
| 205923_at | 5649 | RELN | 0,0358286 | 0,04180002 | 2,10576 | 1,07434 | 2,10576 |
| 222616_s_at | 10600 | USP16 | 0,0361524 | 0,04180002 | 1,52881 | 0,612405 | 1,52881 |
| 209257_s_at | 9126 | SMC3 | 0,0362441 | 0,04180002 | 1,81335 | 0,858655 | 1,81335 |
| 208629_s_at | 3030 | HADHA | 0,0363044 | 0,04180002 | 1,54368 | 0,626373 | 1,54368 |
| 213645_at | 55556 | ENOSF1 | 0,0368841 | 0,04218345 | 0,634881 | -0,655443 | -1,5751 |
| 205809_s_at | 8976 | WASL | 0,0370295 | 0,04218345 | 1,67357 | 0,742933 | 1,67357 |
| 206801_at | 4879 | NPPB | 0,0371416 | 0,04218345 | 2,67924 | 1,42182 | 2,67924 |
| 239185_at | 10350 | ABCA9 | 0,0375931 | 0,04250391 | 1,56102 | 0,64249 | 1,56102 |
| 204621_s_at | 4929 | NR4A2 | 0,0379081 | 0,04252681 | 0,539715 | -0,889729 | -1,85283 |
| 223500_at | 10815 | CPLX1 | 0,0380854 | 0,04252681 | 1,58106 | 0,660892 | 1,58106 |
| 201466_s_at | 3725 | JUN | 0,0382473 | 0,04252681 | 0,62059 | -0,688288 | -1,61137 |
| 202992_at | 730 | C7 | 0,0383392 | 0,04252681 | 1,56163 | 0,643056 | 1,56163 |
| 202124_s_at | 66008 | TRAK2 | 0,0384605 | 0,04252681 | 1,50162 | 0,586515 | 1,50162 |
| 201792_at | 165 | AEBP1 | 0,0391377 | 0,0430858 | 1,5799 | 0,659831 | 1,5799 |
| 219932_at | 28965 | SLC27A6 | 0,0395099 | 0,04317874 | 0,545245 | -0,875023 | -1,83404 |
| 202949_s_at | 2274 | FHL2 | 0,0395786 | 0,04317874 | 1,99877 | 0,999114 | 1,99877 |
| 200951_s_at | 894 | CCND2 | 0,0397382 | 0,04317874 | 1,63853 | 0,7124 | 1,63853 |
| 221933_at | 57502 | NLGN4X | 0,040026 | 0,04330399 | 1,56116 | 0,642622 | 1,56116 |
| 208850_s_at | 7070 | THY1 | 0,0409198 | 0,0436122 | 1,60155 | 0,679472 | 1,60155 |
| 219269_at | 79618 | HMBOX1 | 0,0409639 | 0,0436122 | 1,59154 | 0,670425 | 1,59154 |
| 221841_s_at | 9314 | KLF4 | 0,0410997 | 0,0436122 | 0,497638 | -1,00683 | -2,00949 |
| 229115_at | 1778 | DYNC1H1 | 0,0411183 | 0,0436122 | 1,6653 | 0,735781 | 1,6653 |
| 201593_s_at | 55854 | ZC3H15 | 0,0413496 | 0,0436122 | 1,52927 | 0,612839 | 1,52927 |
| 205381_at | 10234 | LRRC17 | 0,0413534 | 0,0436122 | 1,52256 | 0,606503 | 1,52256 |
| 203896_s_at | 5332 | PLCB4 | 0,0418121 | 0,04387229 | 1,52821 | 0,61184 | 1,52821 |
| 200998_s_at | 10970 | CKAP4 | 0,0419496 | 0,04387229 | 1,63077 | 0,705558 | 1,63077 |
| 226211_at | 55384 | MEG3 | 0,0437138 | 0,04552765 | 1,61332 | 0,690032 | 1,61332 |
| 211726_s_at | 2327 | FMO2 | 0,043955 | 0,04558969 | 1,73405 | 0,794145 | 1,73405 |
| 204931_at | 6943 | TCF21 | 0,0442722 | 0,04563869 | 1,50599 | 0,590711 | 1,50599 |
| 215506_s_at | 9077 | DIRAS3 | 0,0443659 | 0,04563869 | 1,7652 | 0,819828 | 1,7652 |
| 241457_at | --- | --- | 0,0451303 | 0,04623553 | 1,50965 | 0,594214 | 1,50965 |
| 201645_at | 3371 | TNC | 0,0458106 | 0,04661964 | 1,93695 | 0,953787 | 1,93695 |
| 212190_at | 5270 | SERPINE2 | 0,0458767 | 0,04661964 | 1,87934 | 0,910228 | 1,87934 |
| 209460_at | 18 | ABAT | 0,0465435 | 0,04710653 | 1,60735 | 0,684686 | 1,60735 |
| 229346_at | 10763 | NES | 0,0469753 | 0,04735261 | 1,70468 | 0,769504 | 1,70468 |
| 212107_s_at | 1660 | DHX9 | 0,0477567 | 0,04794773 | 1,53334 | 0,616675 | 1,53334 |
| 221731_x_at | 1462 | VCAN | 0,0488473 | 0,0488473 | 1,76909 | 0,823006 | 1,76909 |
