# Supplementary material for: Molecular Signatures of Human Chronic Atrial Fibrillation in Primary Mitral Regurgitation
Source: Cardiovasc Ther. 2021 Oct 15;2021:5516185. doi: 10.1155/2021/5516185 (PMC8538404; doi:10.1155/2021/5516185)
Supplement: Supplementary 10 — Supplementary Table 9: comparison of QPCR gene expression values between AFib vs. SR groups. [file 5516185.f10.docx]

**Supplementary Table 9:** Comparison of QPCR gene expression values between AFib vs. SR groups.

| Genes | Groups | | *P value* | FDR |
| --- | --- | --- | --- | --- |
|  | **SR  (*n*=8)** | **AFib (*n*=8)** |  |  |
| NPPB | 0.7 (0.3-2.2) | 37.6 (4.4-67.4) | 0.002 | 0.017 |
| ANGPTL2 | 0.7 (0.3-1.9) | 7.0 (5.2-22.3) | 0.002 | 0.017 |
| COLQ | 0.8 (0.4-2.4) | 13.9 (7.9-114.2) | 0.003 | 0.017 |
| IGFBP2 | 1.1 (0.3-1.5) | 4.3 (1.9-6.3) | 0.010 | 0.043 |
| BMP7 | 1.0 (0.4-1.5) | 0.3 (0.1-0.5) | 0.021 | 0.071 |
| COMP | 0.5 (0.2-4.7) | 13.3 (1.4-409.0) | 0.028 | 0.072 |
| DNAJA4 | 0.8 (0.6-1.2) | 3.5 (2.6-11.0) | 0.038 | 0.072 |
| DHRS9 | 0.7 (0.5-1.1) | 4.1 (2.3-23.8) | 0.038 | 0.072 |
| CHGB | 0.8 (0.5-1.4) | 5.9 (1.7-32.3) | 0.038 | 0.072 |
| ATP1B4 | 0.6 (0.5-1.6) | 1.4 (0.7-14.1) | 0.195 | 0.332 |
| EXT1 | 0.9 (0.7-1.1) | 2.2 (0.4-5.5) | 0.279 | 0.395 |
| TRDN | 1.1 (0.8-1.7) | 1.3 (1.1-2.5) | 0.279 | 0.395 |
| MCOLN3 | 0.6 (0.4-5.2) | 0.4 (0.1-1.4) | 0.328 | 0.429 |
| CACNB2 | 0.3 (0.1-15.4) | 2.0 (0.6-2.5) | 0.382 | 0.464 |
| AKAP3 | 1.1 (0.6-1.4) | 1.2 (0.5-10.9) | 0.645 | 0.731 |
| RELN | 0.6 (0.3-3.6) | 3.1 (0.3-7.6) | 0.798 | 0.848 |
| TNNI1 | 0.8 (0.3-1.3) | 1.2 (0.2-5.7) | 0.999 | 0.999 |

Values are expressed as median (1^st^-3^rd^ quartiles). p values are adjusted using Benjamini Hochberg procedure.

**Statistical Analysis of QPCRs**

Mann-Whitney U test was applied to compare the gene expression values between study and control groups. To control for the multiple testing, *p* values are adjusted using the Benjamini-Hochberg procedure. The analysis was conducted using TURCOSA (Turcosa Analytics Ltd Co, Turkey, [www.turcosa.com.tr](http://www.turcosa.com.tr)) statistical software. False discovery rates of less than 10% were considered as statistically significant.
