# Supplementary material for: Molecular Signatures of Human Chronic Atrial Fibrillation in Primary Mitral Regurgitation
Source: Cardiovasc Ther. 2021 Oct 15;2021:5516185. doi: 10.1155/2021/5516185 (PMC8538404; doi:10.1155/2021/5516185)
Supplement: Supplementary 11 — Supplementary Table 10: bioinformatics roadmap. [file 5516185.f11.docx]

**The following steps were followed to choose the genes to validate our hypothesis:**

1. List 1: Right SR vs Right AF upregulated genes list = 97 genes

2. List 2: Left SR vs Left AF upregulated genes list = 126 genes

3. List 3: Right+Left SR vs Right+Left AF upregulated genes list = 88 genes

4. Table 3 of the manuscript: The top 15 most upregulated gene list (Right+Left SR vs Right+Left AF)

5. List 1 ∩ List 2 ∩ List 3 ∩ Table 3 = 14 upregulated genes

| NPPB | RELN |
| --- | --- |
| COLQ | RPL3L |
| ATP1B4 | COMP |
| SLC6A6 | ANGPTL2 |
| IGFBP2 | RCAN1 |
| DHRS9 | DPYSL4 |
| CHGB | TNC |

6. List 4: Right SR vs Right AF downregulated genes list = 83 genes

7. List 5: Left SR vs Left AF downregulated genes list = 135 genes

8. List 6: Right+Left SR vs Right+Left AF downregulated genes list = 90 genes

9. Table 3 of the manuscript: The top 15 most downregulated gene list (Right+Left SR vs Right+Left AF)

10. List 4 ∩ List 5 ∩ List 6 ∩ Table 3 = 14 downregulated genes

| MCOLN3 | CEL |
| --- | --- |
| CPLX3 | GPR22 |
| TNNI1 | GADD45G |
| AKAP3 | CACNB2 |
| TRDN | RASGRP2 |
| ASTN2 | BMP7 |
| AQP4 | COL4A6 |

11. Due to budget limitations it was not possible to validate these 28 genes by QPCR. Thus, out of these 28 genes:

a. the genes that were upregulated or downregulated (FC≥2 or FC≤-2) =15 genes

| **Gene** | **Fold Change** |
| --- | --- |
| CPLX3 | -2,43 |
| MCOLN3 | -2,81 |
| TNNI1 | -2,43 |
| TRDN | -2,12 |
| AKAP3 | -2,39 |
| ANGPTL2 | 2,09 |
| ATP1B4 | 2,81 |
| CHGB | 2,37 |
| COLQ | 2,64 |
| COMP | 2,47 |
| DHRS9 | 2,24 |
| IGFBP2 | 2,61 |
| NPPB | 3,46 |
| SLC6A6 | 2,40 |
| RELN | 2,19 |

b. Those that were also up/downregulated in GSE2240 dataset= 19 genes

| ANGPTL2 | MCOLN3 | RCAN1 |
| --- | --- | --- |
| ATP1B4 | TNNI1 | RPL3L |
| CHGB | TRDN | TNC |
| COLQ | AQP4 | BMP7 |
| COMP | ASTN2 | CACNB2 |
| DHRS9 | DPYSL4 |  |
| NPPB | GPR22 |  |

c. Those that appeared in Pubmed search with the following keywords, arrythmia, autophagy and/or cardiac= 20 genes

| ANGPTL2 | MCOLN3 | RCAN1 |
| --- | --- | --- |
| ATP1B4 | TNNI1 | RPL3L |
| CHGB | TRDN | TNC |
| COLQ | AQP4 | BMP7 |
| COMP | IGFBP2 | CACNB2 |
| DHRS9 | RELN | GADD45G |
| NPPB | GPR22 |  |

So out of these 28 genes 16 genes could have been further evaluated with QRT-PCR within the limits of our budget (Supplementary Table 9)
